# Immunogenicity phase II study evaluating booster capacity of nonadjuvanted AKS-452 SARS-Cov-2 RBD Fc vaccine

**DOI:** 10.1038/s41541-024-00830-2

**Published:** 2024-02-21

**Authors:** David G. Alleva, Eline A. Feitsma, Yester F. Janssen, Hendrikus H. Boersma, Thomas M. Lancaster, Thillainaygam Sathiyaseelan, Sylaja Murikipudi, Andrea R. Delpero, Melanie M. Scully, Ramya Ragupathy, Sravya Kotha, Jeffrey R. Haworth, Nishit J. Shah, Vidhya Rao, Shashikant Nagre, Shannon E. Ronca, Freedom M. Green, Stephen A. Shaw, Ari Aminetzah, Schelto Kruijff, Maarten Brom, Gooitzen M. van Dam, Todd C. Zion

**Affiliations:** 1 Akston Biosciences Corporation, 100 Cummings Center, Suite 454C, Beverly, MA 01915 USA; 2 Department of Surgery, University Medical Center Groningen (UMCG), Hanzeplein 1, 9700 RB Groningen, The Netherlands; 3 Department of Nuclear Medicine and Molecular Imaging, UMCG, Groningen, The Netherlands; 4 Department of Clinical Pharmacy and Pharmacology, UMCG, Groningen, The Netherlands; 5 Department of Pediatrics, Division of Tropical Medicine, Baylor College of Medicine and Texas Children’s Hospital, Baylor, College of Medicine, 1102 Bates Ave, 300.15, Houston, TX 77030 USA; 6 TRACER BV, Aarhusweg 2-1/2-2, 9723 JJ Groningen, The Netherlands

**Keywords:** Conjugate vaccines, Conjugate vaccines

## Abstract

AKS-452, a subunit vaccine comprising an Fc fusion of the ancestral wild-type (WT) SARS-CoV-2 virus spike protein receptor binding domain (SP/RBD), was evaluated without adjuvant in a single cohort, non-randomized, open-labelled phase II study (NCT05124483) at a single site in The Netherlands for safety and immunogenicity. A single 90 µg subcutaneous booster dose of AKS-452 was administered to 71 adults previously primed with a registered mRNA- or adenovirus-based vaccine and evaluated for 273 days. All AEs were mild and no SAEs were attributable to AKS-452. While all subjects showed pre-existing SP/RBD binding and ACE2-inhibitory IgG titers, 60–68% responded to AKS-452 via ≥2-fold increase from days 28 to 90 and progressively decreased back to baseline by day 180 (days 28 and 90 mean fold-increases, 14.7 ± 6.3 and 8.0 ± 2.2). Similar response kinetics against RBD mutant proteins (including omicrons) were observed but with slightly reduced titers relative to WT. There was an expected strong inverse correlation between day-0 titers and the fold-increase in titers at day 28. AKS-452 enhanced neutralization potency against live virus, consistent with IgG titers. Nucleocapsid protein (Np) titers suggested infection occurred in 66% (46 of 70) of subjects, in which only 20 reported mild symptomatic COVID-19. These favorable safety and immunogenicity profiles support booster evaluation in a planned phase III universal booster study of this room-temperature stable vaccine that can be rapidly and inexpensively manufactured to serve vaccination at a global scale without the need of a complex distribution or cold chain.

## Introduction

The current regulatory-approved vaccines that utilize the whole spike protein (SP) antigen of the ancestral wild-type (WT) respiratory syndrome coronavirus 2 (SARS-CoV-2) virus have demonstrated transient protection from coronavirus disease 2019 (COVID-19) defined by reductions in hospitalizations and deaths due to infection^1^. However, as mutations in the SARS-CoV-2 genome generate new variants with enhanced transmission (e.g., those of the omicron lineage) or virulence capacities, these WT/SP-based vaccines have become less effective due to mismatched antigenicity to the infectious variant^2^ or other immunological reasons such as immune imprinting related to first exposure to WT antigens (i.e., *via* infection or vaccinations)^3–6^. While additional doses of such original vaccines were implemented to “boost” titers, the resulting effectiveness against the newer omicron variants was modest^2,7^, thus leaving an unmet need to boost neutralizing titers, at least for those at high-risk of complications with COVID-19. Although mRNA-based vaccines had been redesigned with omicron SP variants in hopes of inducing the appropriate variant-specific response^8^, they were not more effective at enhancing immunogenicity against omicron variants relative to the original vaccines^9–11^. As of October 2023, these bivalent mRNA vaccines have been voided by regulatory authorities and updated with an approved monovalent XBB.1.5 SP antigenic component meant to broaden vaccine-induced immunity providing increased protection against current SARS-CoV-2 XBB-sublineage variants that have accounted for >99% of sequenced SARS-CoV-2 specimens in the United States since September 2, 2023^12^. Despite these efforts to update the antigenic SP components of mRNA vaccines, there remains a significant hesitancy by the public to receive such boosters in part because of local and systemic reactogenicity^13^ attributed predominantly to immunostimulatory constituents of the lipid nanoparticle component of mRNA vaccines^14^. Note that the novel adjuvanted protein subunit COVID-19 vaccine, NVX-CoV2373 (developed by Novavax, Inc.), was recently approved by regulatory authorities in October 2023^12^ which showed significantly less reactogenic side effects than the mRNA vaccines^15^.

Considering the reduced reactogenicity profile of the subunit vaccine format in addition to the need for an effective booster vaccination, we are developing a temperature-stable recombinant subunit vaccine, AKS-452, comprised of the WT SARS-CoV-2 SP/RBD and the human IgG1 Fc region^16^ that has recently demonstrated excellent safety and immunogenicity profiles in phase I/II trials^17,18^. Here, AKS-452, in the absence of adjuvant (intended to reduce reactogenicity), was evaluated at a single subcutaneous dose of 90 µg for booster immunogenicity and safety in a non-randomized, single-group assignment, single center, open-label study (*Anti-COVID19 VaccinaTion AKS-452 BOOSTER, ACT-BOOSTER* Study; NCT05124483; EudraCT: 2021-005509-28) with 71 healthy adults (18 to 64 years) who had completed a full-dosing regimen of a regulatory-approved vaccine. This study investigated the proportion of subjects who respond to the booster dose *via* elevation of protective neutralizing antibody titers, whether a bias exists in booster responsiveness with respect to pre-existing titers, and the degree of cross-reactive protective neutralizing responses against omicron variants.

## Results

### Subject demographics and safety assessment

**Fig. 1:**
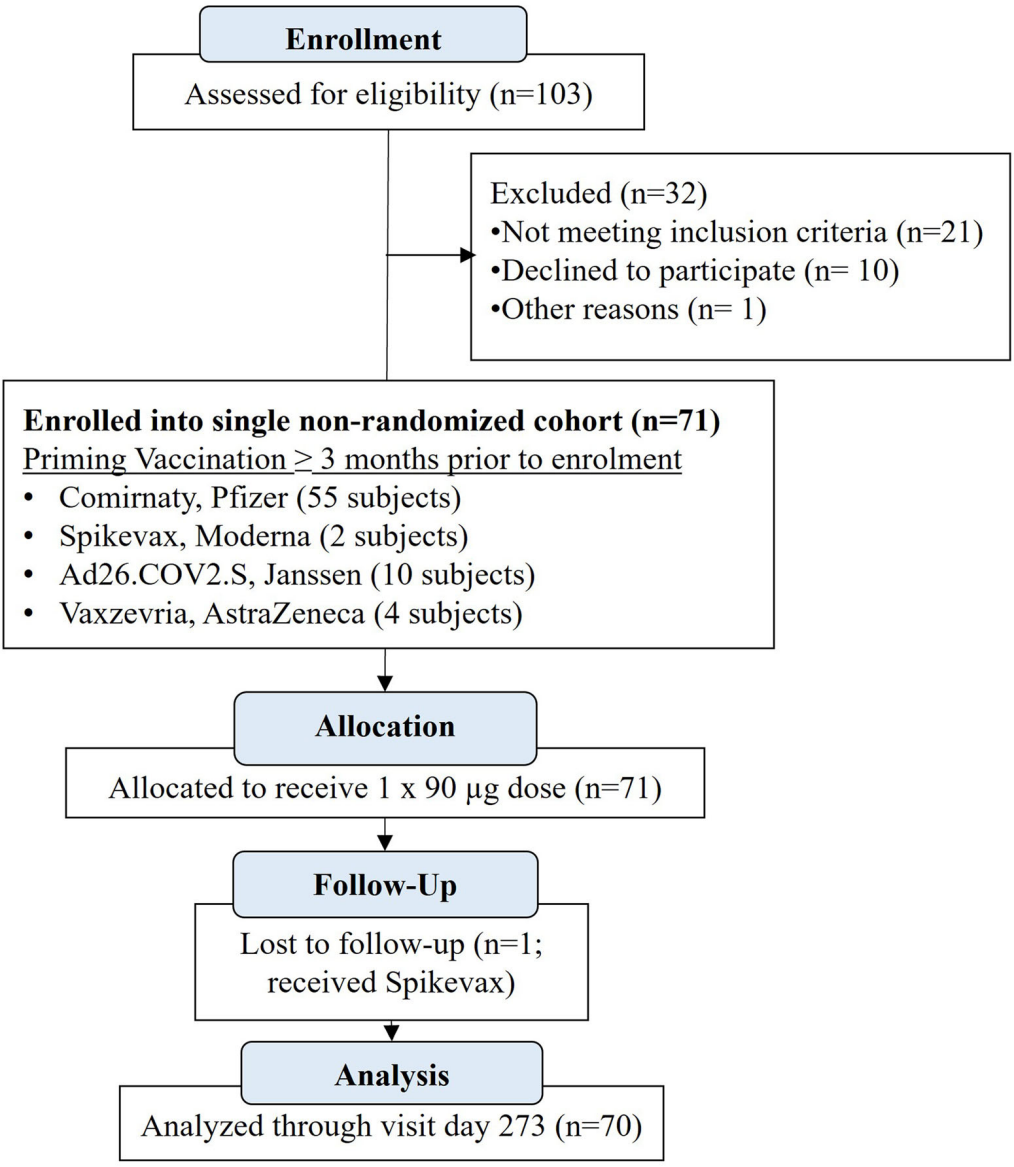
ACT-BOOSTER clinical study design. 71 healthy subjects 18–64 years of age were enrolled in this ACT-Booster study who had completed dosing regimens of a regulatory-approved/-authorized vaccination at least 3 months prior to enrollment (median days, 300 days; range, 113–399 days). Each subject received a single s.c. booster dose of 90 µg of non-adjuvanted AKS-452 and assessed for immunogenicity and safety on days 28, 56, 90, 180, and 273. One subject was lost to follow-up prior to the day-56 visit and therefore was excluded from immunogenicity analyses.

This phase II ACT-BOOSTER study (NCT05124483; EudraCT: 2021-005509-28/ABR 79397) was designed to include 600 healthy volunteer subjects comprised of 4 cohorts of 150 subjects/cohort who had already received a full dosing regimen of one of the following four registered vaccines, respectively; i.e., Pfizer ([Comirnaty], Moderna [Spikevax], Janssen [Ad26.COV2.S], AstraZeneca [Vaxzevria]). While approximately 1000 registrations were received, most were disqualified from participating mainly due to already having received a booster vaccination (via questionnaire during pre-screening). Therefore, only 103 subjects were accepted for screening in which 71 healthy volunteers qualified for and were enrolled in this study, the majority of which had been vaccinated with the Pfizer vaccine (Comirnaty); 55 subjects received Comirnaty, 2 received Spikevax, 4 received Ad26.COV2.S, and 10 received Vaxzevria. Through a protocol amendment (see *Research Protocol, #901452-CT-21-001,* in *Supplementary Material*), a single cohort was formed of the 71-subject aggregate irrespective of previous vaccine type (Fig. 1) comprised of 27 men (38%) and 44 women (62%) with a mean ± s.e.m. age of 30.7 ± 2.8 years (see Table 1 for demographics). None of the 71 subjects reported an adverse event (AE) ≥ grade 3 or a serious adverse event (SAE) at any time during the 273-day study. In fact, all AEs were Grade 1 (mild) (Table 2). There were 47 local AEs and 8 general (i.e., systemic) AEs all related to vaccine dosing reported by 50 subjects post-dose that were collected in a solicited manner, in which all AEs but two appeared within 7 days of dosing and all but 6 resolved within 7 days (Table 2). All individual AEs throughout the study (i.e., from days 0 to 273), including those not related to AKS-452 dosing, are listed in Supplementary Table 1. Twenty subjects acquired COVID-19, all with mild symptoms in which SARS-CoV-2 infection was confirmed by the patient via home testing with a rapid SP-antigen test. While such tests were not specific for a particular viral variant, these infections occurred from 8 June 2022 to 21 November 2022 during the Omicron BA.2/BA.4/5 infection wave that was first announced in April/June 2022^19,20^. These subjects continued with all follow-up visits to the end of the study on day 273. One subject (previously vaccinated with Spikevax) dropped out after the day-28 visit due to strong reactions to blood sampling, thus a total of 70 subjects completed the study per protocol. No irregularities were observed in laboratory analyses in any subject during the entire 273-day trial (Supplementary Table 2).

**Table 1:** Subject characteristics^a^

Prior vaccination	Pfizer	Moderna	AstraZeneca	Janssen	Total
No. of subjects	55	2	4	10	71
Duration from Last Vax dose to AKS-452 dose (days; mean ± SEM)	279 ± 7	308, 321	354 ± 13	324 ± 10	291 ± 12
Sex [N (%)]					
Male	20 (36%)	1 (50%)	2 (50%)	4 (40%)	27 (38%)
Female	35 (64%)	1 (50%)	2 (50%)	6 (60%)	44 (62%)
Age (years)					
Median[range]	29.5	N/A	53	26.9	30.7
	[18–57]	[26, 50]	[24–64]	[19–47]	[18–64]
Race/Ethnicity - N (%)					
Caucasian	53 (96%)	2 (100%)	4 (100%)	10 (100%)	69 (97%)
Other	2 (4%)	0	0	0	2 (3%)
Body Mass Index					
Median[range]	23.5	N/A	22.7	24.2	23.8
	[19.1–33.1]	[26.5, 29.2]	[19.8–29.9]	[20.1–26.4]	[19.1–33.13]
Allergies - N (%)					
Allergy^b^	7 (foods, meds, animals, pollen)	0	1 (food/animals)	1 (food)	9 (12.7%)
Symptomatic COVID-19 prior to enrollment^c^					
N (%)	46 (84%)	0	4 (100%)	9 (90%)	59 (83%)

^a^None of the 71 subjects enrolled had diabetes, cardiovascular, pulmonary, hematologic, rheumatologic, endocrine, autoimmune, or renal diseases, or chronic viral or bacterial infection.

^b^Allergies to foods (nuts/peanut, oranges, pineapple), medications (nitrofurantoin, amoxicillin), grasses/pollen, and/or animals (cat, dog, dust mite); note that no subject was actively on allergy medication during the trial.

^c^The duration between end of symptoms and the AKS-452 dose was 290.7 ± 12.5 (mean ± SEM, days); median day (range, days), 299.5 (113.5–399.4)

**Table 2:** Adverse events

Symptoms^a^	Number of Events^b^	
Local (related to AKS-452 dose)		
Injection site pain	1	2%
Injection site reaction (both redness and swelling)	30 + 2*	68%
Injection site swelling	3	6%
Injection site redness	6 + 1*	15%
Painful arm	3 + 1*	9%
TOTAL	47	
General (related to AKS-452 dose)		
Fatigue	1 + 1* + 1^c^	38%
Muscle ache	1	13%
General malaise	1	13%
Common cold	1	13%
Headache	1	13%
Papular rash	1^c^*	13%
TOTAL	8	

^a^All AEs were collected in a solicited manner during days 0 to 273 after receiving the AKS-452 dose. All AEs were deemed “mild” and all were “Possibly, Probably, or Definitely” Related to Vaccination with AKS-452 except the occurrence of COVID-19. Note that 50 of the 71 total subjects showed AEs related to AKS-452 dosing. There were no Serious Adverse Events (SAE’s) reported in the study.

^b^AEs appeared within 7 days of AKS-452 dose and resolved within 7 days of appearance unless designated with “*“ in which case the AE resolved >11 and < 52 days.

^c^AEs appeared between 12 and 15 days of AKS-452 dose.

### Humoral immunogenicity

**Fig. 2:**
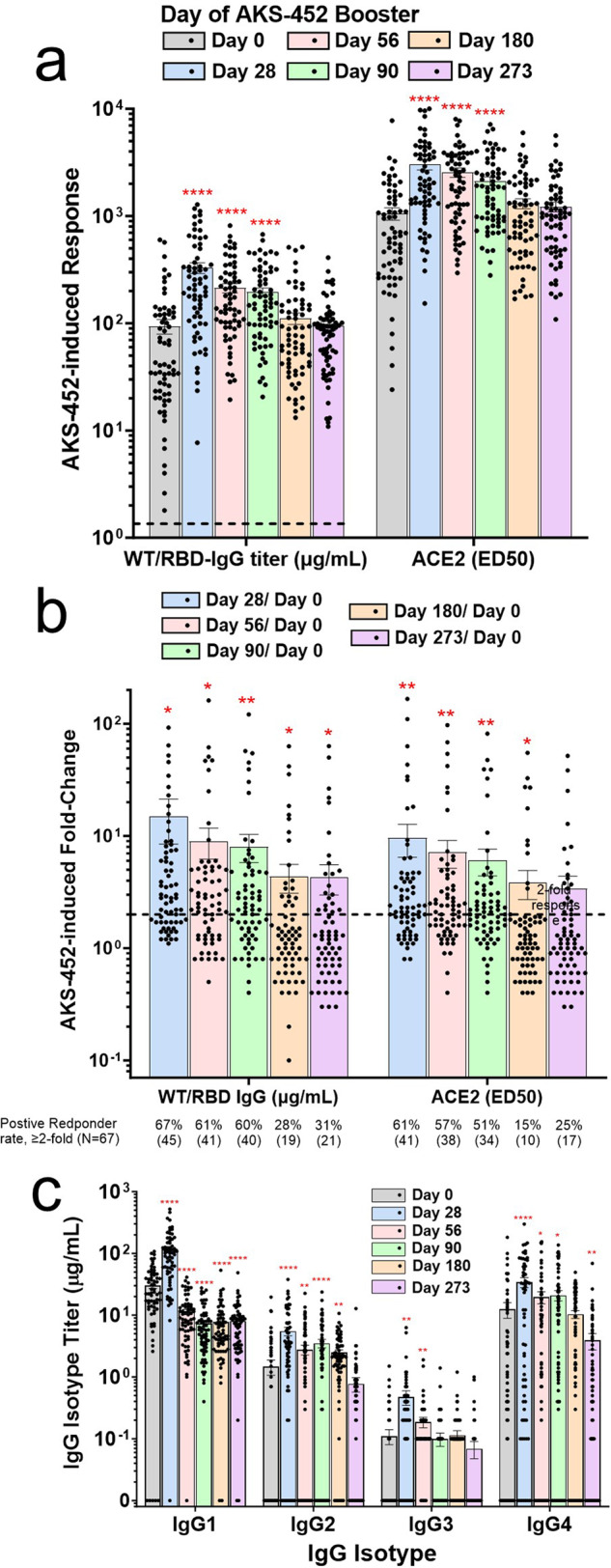
AKS-452 immunogenicity: WT-SP/RBD IgG titers. Serum samples were obtained from 67 subjects on days 0, 28, 56, 90, 180, and 273 after receiving a 90 µg s.c. dose of AKS-452 administered ≥3 months after completion of regulatory–approved vaccinations with Comirnaty (N = 52), Spikevax (N = 1), Ad26.COV2.S (N = 10), or Vaxzevria (N = 4) and assessed for anti-WT-SP/RBD IgG binding titers (via ELISA) and inhibitory potency (ED50) (via ACE2-RBD ELISA) (**a**) and presented as fold-change per subject (**b**). Seroconversion was defined as >1.44 µg/mL IgG (dotted line in panel a; Note that all subjects had titers above the cut-off on day 0 prior to receiving the AKS-452 booster dose). A *positive responder* was defined as having at least a 2-fold increase in titer from day 0 (**b**). Anti-WT-SP/RBD IgG isotype titers were assessed via ELISAs (**c**). Statistical comparisons between mean values of each post-dose day to those prior to dosing (day 0) were performed using the *t* Test equal variance, one-tailed; *, p < 0.05; **, p < 0.01; ***; p < 0.001, ****; p < 0.0001. The mean ± stand error of the mean (s.e.m) are shown in each panel.

Immunogenicity was assessed in 70 subjects, of which three subjects acquired COVID-19 prior to the day 28 visit. Because the day-28 visit defines the clinical endpoint specified in the study protocol, immunogenicity data of the remaining 67 subjects were treated separately from those of the three COVID-19 subjects (Supplementary Fig. 1) to avoid any effects of infection on the activity of AKS-452 during this critical 28-day period. Anti-WT/RBD IgG titers were assessed in response to the single dose of AKS-452 in which geometric mean titers significantly increased on days 28, 56, and 90, and declined back to baseline by days 180 and 273 (Fig. 2a). All 67 subjects had pre-existing anti-WT/RBD IgG titers above the positive cut-off of 1.44 µg/mL on day 0 prior to receiving AKS-452 (median, 41.3; range, 1.8–599.2; Fig. 2a). Therefore, the study protocol immunogenicity endpoint criterion was the *Enhanced Immune Response* to AKS-452 vaccination defined as ≥2-fold baseline SP/RBD IgG titer at day 28 per subject (i.e., a subject with ≥ 2-fold response was scored as a positive responder). Forty five of the 67 subjects were scored as positive responders at day 28 yielding a 67% response rate that was generally maintained out to day 90, after which a substantial decline by day 180 was apparent although still showed a positive response of 31% at day 273 (Fig. 2b). The geometric means ± s.e.m. of the IgG fold-increase on days 28, 56, 90, 180, and 273 were 14.9 ± 6.4, 9.0 ± 2.8, 8.1 ± 2.2, 4.3 ± 1.2, and 4.3 ± 1.3, respectively (Fig. 2b). Similar findings were reflected in inhibition potency of angiotensin converting enzyme-2 (ACE2)-WT/RBD binding with respect to mean ED50 values (Fig. 2a) and the fold-increase response (Fig. 2b). In addition, AKS-452 booster dosing enhanced all IgG isotypes at day 28 of which the effector T helper 1 (Th1)-type IgG1 and IgG3 isotype titers tended to decline more rapidly than Th2-asssociated IgG2 and IgG4 titers (Fig. 2c).

**Fig. 3:**
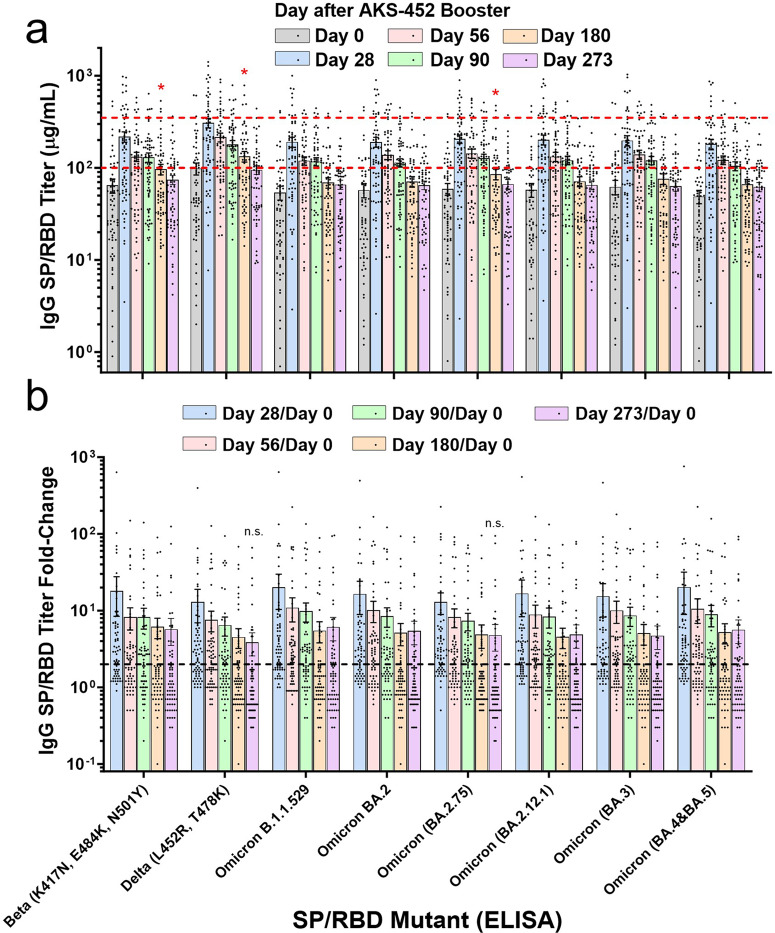
AKS-452 immunogenicity: mutant SP/RBD IgG titers. Serum samples were obtained from 67 subjects on days 0, 28, 56, 90, 180, and 273 after receiving a 90 µg s.c. dose of AKS-452 ≥ 3 months after completing regimens of regulatory–approved vaccines and assessed for IgG binding titers against different mutant SP/RBD antigens via ELISA (**a**, dotted red lines are references of day 0 and day 28 WT means) and presented as fold-change per subject (**b**; dotted black line denotes a 2-fold change). **a** All day-28, -56, and -90 mean values (p < 0.0001) and some day-180 values (*, p < 0.05) were significantly different from respective day-0 mean values (*t* Test equal variance, one-tailed). **b** All geometric mean values of the fold-change response on day 28, 56, 90, 180, and 273 were significantly greater than 2.0 (p < 0.05), except those designated not significant (n.s.). The mean ± s.e.m. are shown in each panel.

All subjects also had pre-existing IgG titers (i.e., day 0) specific to Beta, Delta, and six Omicron variant SP/RBD antigens in which geometric means of titers significantly increased on days 28, 56, and 90 after receiving a single dose of AKS-452 that peaked at day 28 and progressively decreased back to baseline by day 180 (Fig. 3a). Mean titers of the six omicron variants were slightly less than those of WT on days 0 and 28 (see dotted red lines of WT reference; Fig. 3a) although the fold-change in responses to AKS-452 booster at days 28, 56, 90, 180, and 273 relative to the respective day 0 values were similar among variants (Fig. 3b). This kinetic pattern of the variants was similar to that of WT (see Fig. 2b).

**Fig. 4:**
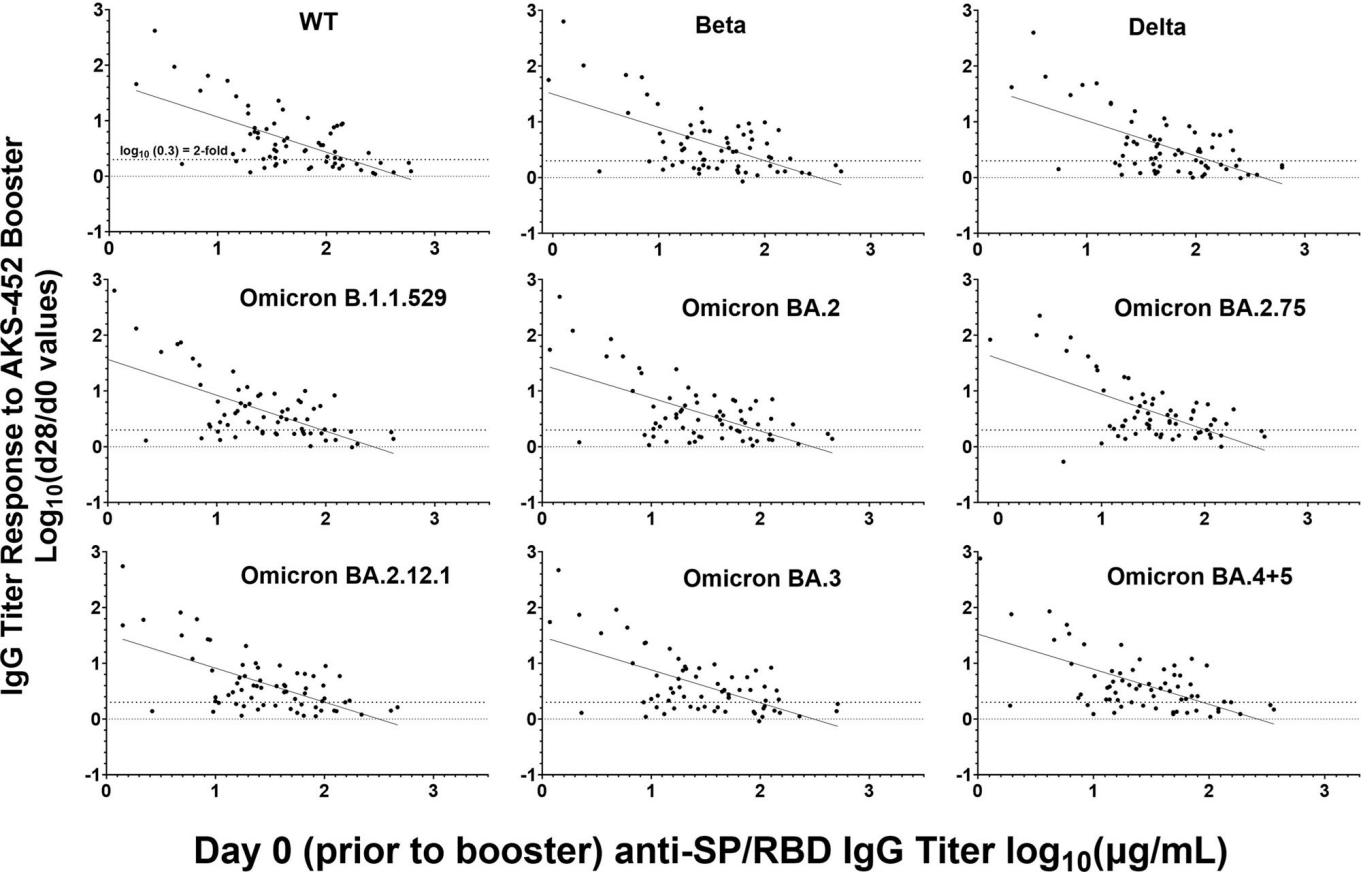
Negative correlation between WT and mutant SP/RBD IgG titers at day 0 vs. the fold-change in titer at day 28. Serum samples were obtained from 67 subjects on days 0 and 28 after receiving the AKS-452 booster dose (90 µg, s.c.) and assessed for IgG binding titers against different mutant SP/RBD antigens via ELISA (x axis) vs. the fold-change in titer per subject at day 28 (y axis). The dotted lines delineate no response (i.e., y = 0) and a 2-fold response (y = 0.3), respectively. For each variant, a bivariate normal distribution fit to X and Y variables was conducted with Log_10_(day-0 titers) vs. Log_10_(day-28/day-0 Ratios), respectively. Correlations within each variant data set were significantly different from 0 (p < 0.0001) with a range of correlation coefficients of −0.599 to −0.647.

There was a significant negative correlation in the ability of a subject to respond to AKS-452 relative to their pre-existing RBD titer that was apparent by plotting pre-booster titers (*i.e*., day 0) against the respective fold-increase at day 28 (Fig. 4). In fact, analysis of such negative correlation demonstrated that there were significant differences in the rate of positive responsiveness (i.e., > 2-fold-increase) of subjects who comprised the lower 87% of day-0 titers vs. those with the upper 13% of day-0 titers (Table 3; see last column for cut-off titers). Positive response rates of all 67 subjects against different RBD mutants ranged from 60 to 71%, which was slightly less than those of the lower 87% day 0-titer subset (N = 58) that ranged from 64–78%. However, the positive response range of the upper 13% subset was only 11–44%, demonstrating that those with high pre-existing titers are less likely to respond to an AKS-452 booster dose than those with lower titers. In addition to the response rates, the lower 87% day-0 titer subset showed a range of mean fold-increase of 18.7 to 30.2, whereas that of the upper 13% was only 2.6 to 5.5. Of even greater significance, the range of maximum fold-increase of the lower 87% was 420 to 759 which was substantially greater than that of the upper 13% that was 2.6 to 9.0, further demonstrating that greater responsiveness to the AKS-452 booster was associated with those subjects having lower pre-existing titers. These results provide a basis for which the cut-off titer values that delineate the lower 87% from the upper 13% (Table 3) could be used in future studies as an acceptance criterion for whether a subject requires a booster.

**Table 3:** Positive-responder rates of mutant SP/RBD IgG titers at day 28 of AKS-452 vaccination

Positive responders^a^ within											
	100% of subjects (N = 67) ^b^		lower 87% of subjects ranked by day-0 titers (N = 58) ^b^				upper 13% of subjects ranked by day-0 titers (N = 9) ^b^				87%/13% day-0 cut-off titer (ug/mL) ^c^
SP/RBD variant	N	% of N = 67	N	% of N = 58	mean SI (SEM)	Max SI	N	% of N = 9	mean SI (SEM)	Max SI	
WT (ACRO)	45	67%	44	76%	21.9 (9.7)	420	1	11%	2.6 (n/a)	2.6	150
Delta (L452R, T478K)	40	60%	37	64%	22.1 (10.7)	397	3	33%	3.9 (1.4)	6.7	190
Beta (K417N, E484K, N501Y)	42	63%	39	67%	29.7 (16.3)	636	3	33%	3.8 (1.6)	7.1	113
Omicron B.1.1.529	45	67%	43	74%	30.2 (15.0)	637	3	33%	4.1 (2.1)	8.4	99
Omicron BA.2	44	66%	41	71%	25.9 (12.3)	494	3	33%	4.2 (1.4)	7.0	99
Omicron (BA.2.75)	46	69%	44	76%	18.7 (6.0)	225	4	44%	3.3 (0.5)	4.7	115
Omicron (BA.2.12.1)	46	69%	42	72%	25.4 (13.2)	553	4	44%	4.7 (1.7)	5.9	100
Omicron (BA.3)	44	66%	40	69%	24.5 (11.7)	464	4	44%	4.0 (1.5)	8.3	112
Omicron (BA.4&BA.5)	47	70%	45	78%	29.3 (16.9)	759	3	33%	4.2 (2.4)	9.0	95

^a^A “positive responder” subject was defined as having > 2-fold change in anti-SP/RBD IgG titer from day-0 to day-28 after a single 90 µg AKS-452 vaccination dose.

^b^Day-0 mean IgG titers from all 67 subjects per mutant SP/RBD ELISA were rank ordered and grouped by the lower 87% (i.e., 58 subjects) and the upper 13% (i.e., 9 subjects) and positive responder rates, mean ± SEM of the fold-change in titer (stimulation index, SI), and the Maximum SI of the positive responders were determined.

^c^The IgG titer of the subject defining the cut-off between the lower 87% and the upper 13% of the rank ordered titers.

### Neutralization potency of live SARS-CoV-2 viral variants via the Plaque Reduction Neutralization Test (PRNT)

**Fig. 5:**
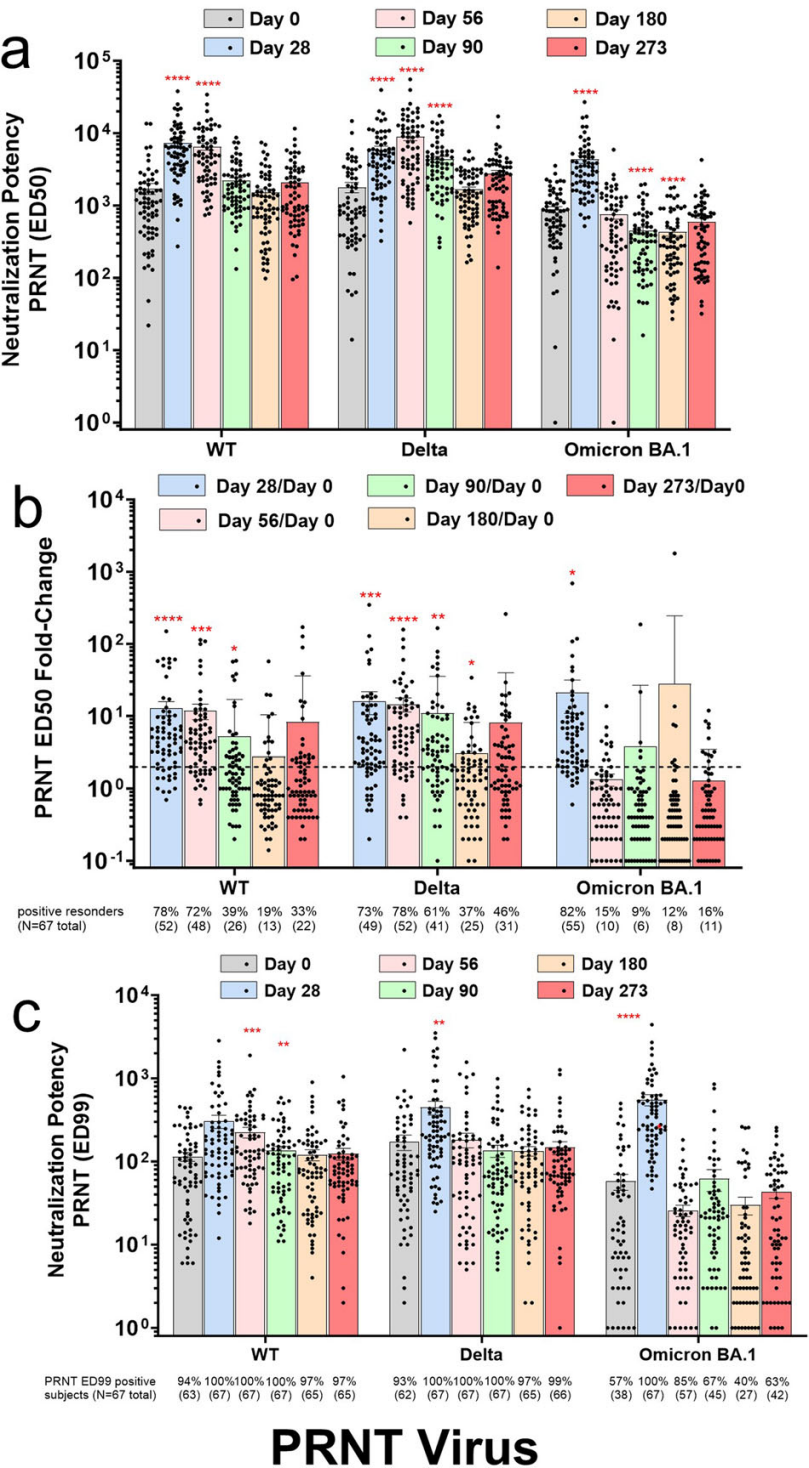
AKS-452-induced immune serum neutralization of live WT, Delta, and Omicron BA.1 virus PRNT. Serum samples were obtained from 67 subjects on days 0, 28, 56, 90, 180, and 273 after receiving a 90 µg s.c. dose of AKS-452 ≥ 3 months after completing regimens of regulatory–approved vaccines. Serial dilutions of sera were assessed for % neutralization of the WT, Delta, and Omicron BA.1 live virus strains to infect live VERO E6 cells via the PRNT. The effective dilution 50% (ED50) (**a**) and 99% (ED99) (**c**) values were determined for each sample using non-linear regression log(agonist) vs. response analysis (i.e., represented as 1/dilution) and the group mean ± s.e.m. are presented. *, p < 0.05; **, p < 0.01; ***, p < 0.001; ****, P < 0.0001; geometric mean values were significantly different from the respective day-0 values (*t* test, equal variance, one-tailed). The fold-change of ED50 values on days 28, 56, 90, and 180 relative to the respective day-0 values for each sample were determined in which a positive responder sample was defined as ≥2-fold increase (dotted line) and presented as the number (and % of total) positive (**b**; significantly greater than 2-fold; *, p < 0.05; **, p < 0.01; ***; p < 0.001, ****; p < 0.0001.). In addition to the regression-generated ED99 value, a sample was scored in a binary manner for whether it achieved 100% viral neutralization at ≥1:40 dilution (**c**).

The AKS-452 booster dose also enhanced serum potency (ED50) to neutralize the live SARS-CoV-2 viral strains, WT, Delta, and Omicron BA.1, from infecting live cells *via* the PRNT (Fig. 5a). The kinetic profile was similar to those obtained for IgG titers in that neutralization ED50 geometric means were significantly enhanced by day 28 after the AKS-452 booster dose among the three viral variants in which those of WT and Delta were maintained longer than those of Omicron BA.1. Note that mean ED50 responses to Omicron BA.1 significantly decreased below baseline by days 90 and 180 (Fig. 5a). On the basis of individual subject responses, the mean ED50 response values on days 28, 56, 90, 180, and 273 relative to day-0 values showed a kinetic pattern expected from the mean titer pattern (Fig. 5b). Subjects showing a ≥ 2-fold increase in ED50 from baseline values were scored as positive responders from which responder rates were derived (i.e., % positive responders). Responder rates on days 28 and 56 for WT and Delta were between 72–78% that progressively decreased to 33 and 46% by day 273, respectively, whereas those of Omicron BA.1 were slightly greater than those of WT and Delta on day 28 (i.e., 82%), but abruptly decreased to 9 to 16% during days 56, 90, 180, and 273.

**Fig. 6:**
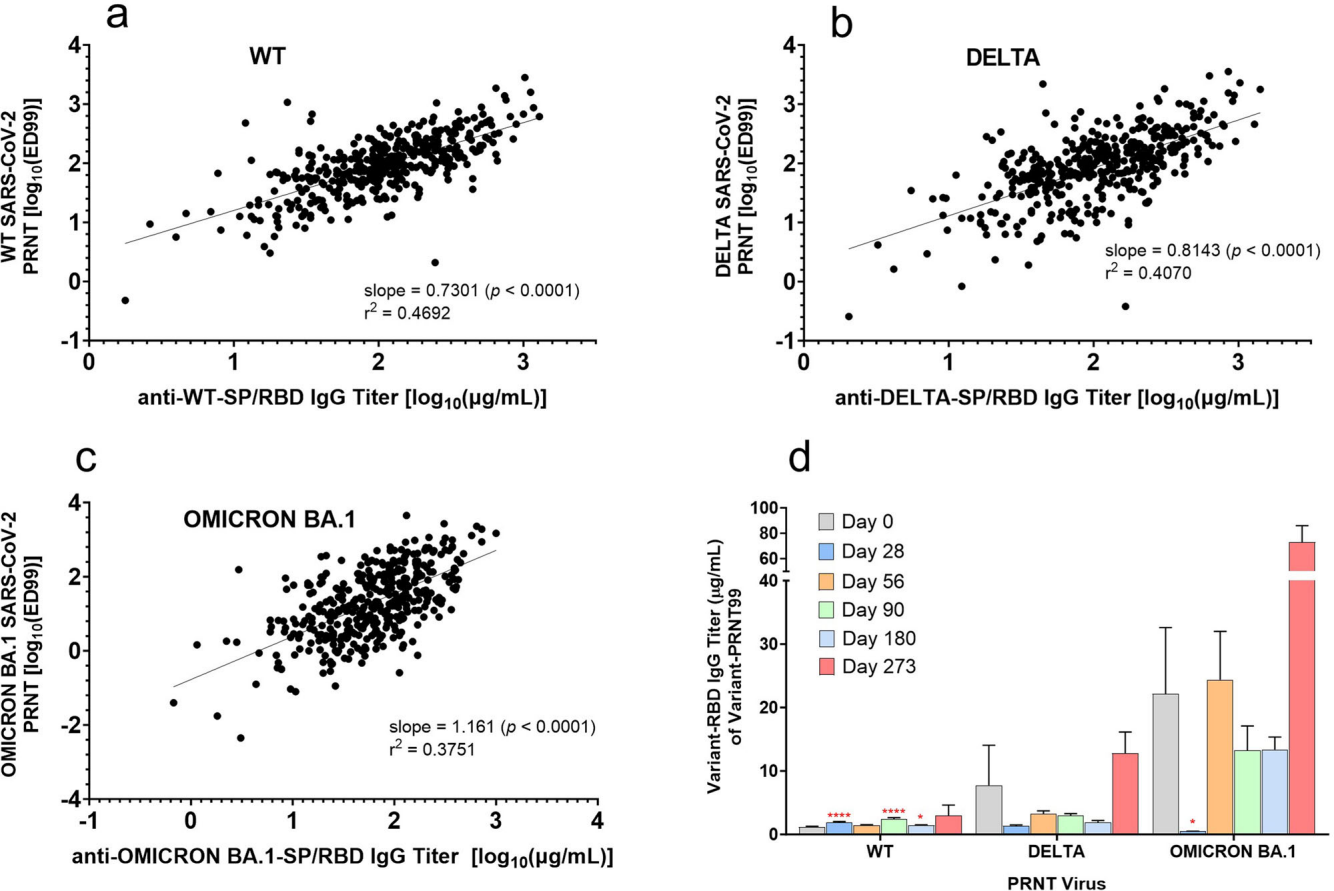
Relationship between anti-SP/RBD IgG titer (ELISA) and PRNT neutralization potency (ED99) among WT, Delta, and Omicron BA.1 variants. Serum samples were obtained from 67 subjects on days 0, 28, 56, 90, 180, and 273 after receiving a 90 µg s.c. dose of AKS-452 ≥ 3 months after completing regimens of regulatory–approved vaccines. Anti-SP/RBD titers and the ED99 values were determined for WT (**a**), Delta (**b**), and Omicron BA.1 (**c**) variants and linear regression analyses were performed on log_10_ values. For each variant, a bivariate normal distribution fit to *x* and *y* variables was conducted with log_10_(IgG titer) vs. log_10_(ED99), respectively, in which correlations within each variant data set were significantly different from 0 (p values of slope) with correlation coefficients for WT, Delta, and Omicron of 0.730, 0.644, and 0.610, respectively. **d** The concentration of anti-SP/RBD IgG (ELISA) at which 99% neutralization occurred (PRNT ED99) was determined as the “specific potency of neutralization” ([IgG µg/mL]/ED99; note that decreasing values correlate with increasing potency). *, p < 0.05; ****, p < 0.0001; geometric mean values (± s.e.m.) were significantly different from the respective day-0 values (*t* test, equal variance, one-tailed).

Although the ED50 is an accurate measure of neutralization *potency*, it does not reflect the *extent* of neutralization. Therefore, we determined whether a sample achieved 100% neutralization at the highest concentration of serum used in the PRNT (i.e., 1:40 dilution) via binary scoring (i.e., positive score was 100% neutralization at ≥1:40 dilution) (Fig. 5c). In addition, the precise dilution at which 100% neutralization occurred was calculated as the ED99 value via non-linear regression analysis (Fig. 5c). Strikingly, while the 100% neutralization rate against WT and Delta viruses was 93 to 100% from baseline to day 273, that of Omicron BA.1 showed a dramatic increase from 57% at baseline to 100% at day 28 (Fig. 5c). The kinetic pattern of ED99 mean values reflected those of the 100% neutralization responder rates. In addition, the ED99 mean kinetic changes also reflected those of the variant IgG SP/RBD binding titers showing a positive correlation in changes between the two metrics (Fig. 6a–c). This positive correlation enabled the determination of the “specific neutralization potency” of each sample, which is the concentration (in µg/mL) of the variant-specific IgG of the dilution at which 99–100% of cells were protected from the respective variant infection (*i.e*., the ED99; Fig. 6d). This specific neutralization potency analysis (i.e., [IgG µg/mL]/ED99) demonstrated that anti-omicron BA.1 IgG specific potency was much less than those of WT and Delta variants on day 0 (i.e., higher µg/mL values denote lower potency; Fig. 6d), but dramatically increased by day 28 in response to boosting with AKS-452 (Fig. 6d). However, this enhanced specific potency of omicron-specific titers was transient and decreased to baseline levels by day 56.

### Anti-Np IgG titer changes as a correlate to SARS-CoV-2 infection

**Fig. 7:**
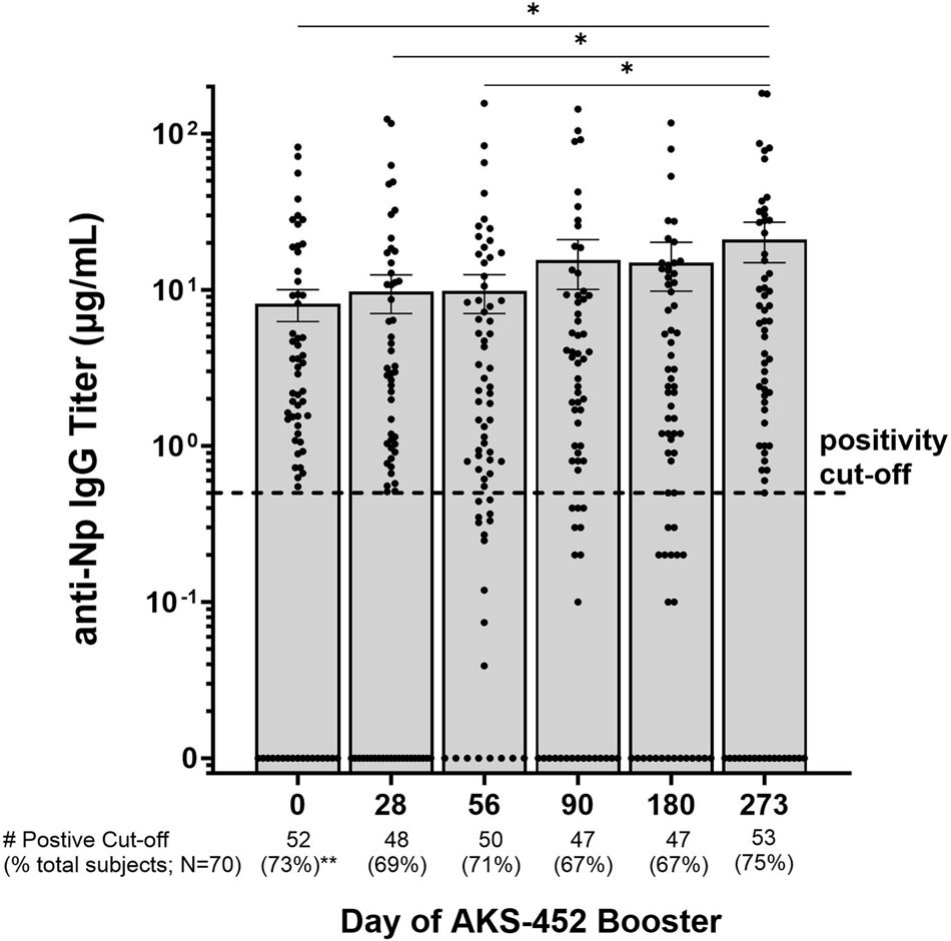
Nucleocapsid Protein (Np) IgG titers. Serum samples were obtained from 70 subjects on days 0, 28, 56, 90, 180, and 273 after receiving a 90 µg s.c. dose of AKS-452 administered ≥3 months after completion of regulatory–approved vaccinations and assessed for anti-Np IgG binding titers via ELISA of which a positive cut-off was 0.5 µg/mL, dotted line (geometric mean ± s.e.m.). *, p < 0.05; geometric mean values were significantly different from the respective day-0 values (t test, equal variance, one-tailed). ** N = 71 subjects (includes 1 dropout subject).

A positive titer for anti-Np IgG is considered a marker of prior exposure to SARS-CoV-2 infection^21–24^. In this study, 73% (52/71) of subjects showed positive anti-Np IgG titers at baseline (day 0; positivity cut-off = 0.5 µg/mL; Fig. 7) and 83% (59/71) of subjects reported having COVID-19 prior to this study (see Table 1). The association of anti-Np positivity with COVID-19 prior to this study was evident in that 52 of the 59 subjects (88%) who reported prior COVID-19 were also positive for anti-Np IgG titers on day 0, and 10 of the 12 subjects (83%) who did not report prior COVID-19 were negative for anti-Np IgG. The percentage of subjects who had positive anti-Np IgG titers throughout the 273-day study fluctuated between 64 and 75% (Fig. 7). Importantly, 46 of the 70 total subjects that completed the trial showed a significant increase in anti-Np IgG titers during a specific interval of the entire 273-day trial (i.e., a spike in titer >2-fold), an event that presumably reflects an infection during that interval (Supplementary Table 3 and Supplementary Fig. 2). Of these 46 subjects showing positive anti-Np IgG titers, only 20 reported COVID-19 symptoms (all mild) (Supplementary Table 3 and Supplementary Fig. 2). Therefore, recent infection measured by anti-Np titers correlates with recent symptomatic disease, and that such infection is measured exclusively from symptoms because 26 of the 46 anti-Np sero-enhanced subjects (i.e., infected) did not show symptoms (Supplementary Table 3). Moreover, AKS-452-induced anti-RBD titers appear to associate with reduced symptomatic disease incidence, but not infection, because the proportion of subjects reporting COVID-19 symptomatic disease prior to enrollment was 83% (59 COVID + /71 total subjects; see Table 1) which was reduced to 29% (20 COVID + /70 total subjects; see Supplementary Table 3) after AKS-452 dosing. Specifically, the ratio of subjects with symptomatic COVID-19 versus the total infected (anti-Np positivity) prior to AKS-452 enrollment was 1.2 (i.e., 59 COVID + /50 Np + ) which was dramatically reduced by 2.3-fold to 0.43 after AKS-452 dosing (i.e., 20 COVID + /46 Np+ spike). Moreover, changes in anti-Np IgG titers did not correlate with changes in anti-RBD IgG titers after AKS-452 dosing demonstrating RBD-specificity of AKS-452 vaccination (Supplementary Table 3, Supplementary Figs. 1, 2), which suggests that SARS-CoV-2 infection did not confound the immunogenicity of AKS-452 vaccination.

## Discussion

Safety assessment of a AKS-452 booster vaccination in this ACT-BOOSTER phase II clinical study showed limited side-effects in which no SAEs were attributable to vaccine dosing, and only a few mild AEs were associated with dosing that were comparable to or less than those of other registered COVID-19 vaccines^25^. No relevant laboratory assessment abnormalities were observed. Accordingly, a similar safety profile was evident in the 180-day predecessor phase I/II trials^17,18^. Note that while the Montanide™ adjuvant strongly enhances IgG titers in COVID-19-immunologically naïve subjects^17,18^, it is also known to strongly contribute to local injection site AEs^26^, in which the non-adjuvanted AKS-452 booster approach in immunologically-primed (i.e., priorly vaccinated) subjects avoids any untoward adjuvant effects.

The immunogenicity profile of the AKS-452 booster dose demonstrated a strong enhancement of IgG titers that correlated with ACE2-SP/RBD inhibition titers and live-virus neutralization potency, all features that are consistent with immunogenicity profiles of the previous AKS-452 phase I/II trials^17,18^. Indeed, the non-adjuvanted AKS-452 in this study potently enhanced preexisting anti-SP/RBD IgG titers, which is consistent with our preclinical results demonstrating that a previously-infected non-human primate strongly responded to non-adjuvanted AKS-452^16^, and with results from phase I/II demonstrating a non-adjuvanted AKS-452 dose administered 180 days after a single adjuvanted priming dose to five subjects induced a robust response 28 days later (i.e., day 208) in all five subjects^17^.

This ACT-BOOSTER study was the first opportunity to demonstrate that such AKS-452-derived titers strongly neutralize selected omicron variants *via* the PRNT. While the mean values of SP/RBD IgG titers and PRNT potencies (ED50 and ED99 values) of omicron variants were <2-fold below those of WT during the 273-day duration, the mean magnitude of AKS-452-induced responses from baseline (i.e., fold-increase) were similar. Furthermore, although omicron SP/RBD interacts with the human ACE2 target via different molecular interactions than that of WT [29, 30], omicron SP/RBD-specific IgG are not required for neutralization because the WT SP/RBD antigen of AKS-452 clearly induced cross-reactive neutralizing IgG titers against omicron, albeit in a transient manner relative to those against WT and Delta. This selective and transient neutralizing response against omicron is reflected in the strong enhancement of the *specific neutralization potency* against omicron (neutralization ED99 per µg IgG) relative to those against WT and Delta variants (see Fig. 6). While additional investigations are required to uncover how AKS-452 induces this omicron-specific neutralization potency, it may be reflected in conditions of immune imprinting caused by prior vaccination with WT whole SP antigen or infection in which a de novo induction of an anti-omicron SP/RBD cross-reactive population of B cells could be transiently induced by AKS-452, as demonstrated with other vaccine conditions^4,6,27^. That is, existing antigenically related specific memory B cells can robustly outcompete the generation of new cross-reactive focused B cell responses within germinal centers of lymph nodes leading to a transient response (*i.e*., immune imprinting)^5^. It may be that the few relevant epitopes contained in the RBD of AKS-452 relative to the many irrelevant epitopes contained in the entire SP antigen of other vaccines could lead to a more focused immune response that avoids interference from the other irrelevant responses. Indeed, neutralizing titers against the WT and omicron BA5 strains were strongly induced *via* a WT SP/RBD-based vaccine^28^, and it will be helpful to know the immunogenicity outcomes of an Alum-formulated omicron-specific SP/RBD-Fc antigen vaccine^29,30^.

While this study was not designed to demonstrate efficacy towards infection or disease symptoms, pre-enrollment and post-dosing anti-Np titer infection parameters along with symptomatic COVID-19 incidence shed light on the possibility of efficacy of the non-adjuvanted AKS-452 booster dose. Importantly, assessments of anti-SP/RBD binding IgG titers and neutralization potency were demonstrated to be strong correlates of protection against virus-positive symptomatic COVID-19 disease onset and severity *via* different statistical causal inference frameworks used to analyze data from the mRNA-1273 COVE Trial (i.e., Moderna vaccine)^31^ and following multiple booster doses of the BNT162b2 vaccine (i.e., Pfizer vaccine)^32^. Nevertheless, efficacy of AKS-452 must be addressed in future placebo-controlled studies with larger cohort sizes.

This study was limited in that it did not contain a negative control placebo group required for setting the relevant baseline values at each interval after AKS-452 dosing so that the true immunogenicity of AKS-452 could be accurately assessed at each visit throughout the 273-day study. That is, AKS-452-induced titers that subsided to day-0 “baseline” values at days 90, 180, or 273 could be much greater relative to declining control values that would have existed in the absence of AKS-452 boosting. Another limitation was that the effect of the prior specific vaccination type on the response to AKS-45 booster could not be evaluated because of the disparate sizes of the different vaccination groups. Another limitation of the study was that definitive modulation of the immune response phenotype was not performed via antigen-specific cellular cytokine production, but rather was inferred via IgG isotype profile. Nevertheless, the IgG isotype profile induced by the AKS-452 booster was slightly different from that of the adjuvanted formulation of AKS-452 used previously^17,18^ in that the non-adjuvanted booster enhanced all IgG isotypes (Th1- and Th2-associated) at day 28 whereas the adjuvanted formulation stimulated a dominant Th1-biased response (i.e., IgG1 and IgG3). Whether this skewed response is due to adjuvant and/or naïve^17,18^ vs. primed (vaccinated/infected) subjects remains to be determined. This study was also limited by the lack of opportunity to evaluate neutralizing titers against more recent strains of SARS-CoV-2, such as XBB.1.5, BA.2.86, and EG.5, that will be explored in future trials.

In summary, results of this phase II ACT-BOOSTER study support that the AKS-452 vaccine without adjuvant could potentially be administered in multiple booster doses to enhance the protective anti-SP/RBD antigenic immune response with higher affinity neutralizing antibodies against infective variants of SARS CoV-2. Importantly, a major advantage of using AKS-452 in the absence of adjuvant allows for a less costly and more streamlined formulation process. Furthermore, each 2,000 L GMP manufactured batch yields approximately 50 million 90 µg doses of AKS-452, a capacity that improves on the costs per dose relative to viral, nucleic acid, and full-length recombinant SP subunit-based vaccines.

## Methods

### Vaccine components

AKS-452 is a recombinant fusion protein comprising SP/RBD and an Fc fragment containing a portion of the hinge region, in which the full CH2 and CH3 domains of the human IgG1 Fc fragment are connected via a covalent peptide linker sequence, all encoded by a single nucleic acid molecule expressed in CHO-K1 cells as previously described^16,18^ (drug substance #MDS0006, 3290 µg/ml; Akston Biosciences, Beverly, MA; see detailed methods published elsewhere^17^). The expression yield was 0.75 g/L for material used in this study and has since been optimized to approximately >2.00 g/L, compared to less than 0.1 g/L for non-Fc modified full-length SP produced in the same expression system. This drug substance was manufactured into sterile drug product at ICON plc (Groningen, Netherlands) in vials containing 1 mL of AKS-452 at 3,147 µg/mL (#TGR20644/ AKS452X/10JUN21). The batch was >98% pure with respect to molecular aggregates *via* SEC-HPLC and fragments *via* capillary electrophoresis-sodium dodecyl sulfate (CE-SDS) analysis (see detailed methods published elsewhere^17^ and Supplementary Table 4 for drug substance production and characterization details). AKS-452 drug substance and drug product were released for clinical use from ICON (Groningen, The Netherlands) after passing established criteria (Supplementary Tables 4 and 5, respectively) and were stored at −80 °C. This sterile aqueous solution of AKS-452 was diluted by 5-fold in a sterile saline solution and administered to subjects within 24 h of reconstitution and within 4 h of being drawn into syringes (details of manufacturing, stability, and clinical formulations are published elsewhere^17^). Data from stability studies support storage at −80 °C, 2–8 °C, and 25 °C for at least twelve months (see Supplementary Table 6).

### Study design and procedures

Subjects were recruited in March-April 2022 during the Omicron variant wave of the COVID-19 pandemic that began in December 2021 (i.e., Omicron BA.1 variant along with the Delta variant became prevalent in January 2022, Omicron BA.2 was prevalent by April 2022, and Omicrons BA.4 and BA.5 became prevalent in June 2022^19^). The first subject was enrolled on 21 April 2022 and the last subject’s final visit occurred on 16 March 2023. Main exclusion criteria were receipt of previous “booster vaccination” and use of corticosteroids (excluding topical preparations for cutaneous or nasal use) or other immunosuppressive drugs within 30 days prior to the AKS-452 vaccination dose. The main inclusion criteria were that each subject be a healthy adult (18 to 85 years) and must have completed a full-dosing regimen (but not a booster dose) of one of four regulatory-approved vaccines ≥3 months of enrollment [two doses of Comirnaty (Pfizer-BioNTech, New York, NY), two doses of Spikevax (Moderna, Cambridge, MA), one dose of Ad26.COV2.S (Janssen, Raritan, NJ), or two doses of Vaxzevria (AstraZeneca, Gaithersburg, MD)^33–35^]. Testing of SARS-CoV-2 infection was actively performed in each subject prior to AKS-452 dosing during screening *via* an EUA-approved PCR test (*AlinityM* SARS-CoV-2 Assay, Abbott Molecular, Inc.; positive result of infection was cycle number ≤ 36). After enrollment during the study, each subject was prompted to take a rapid antigen test at home to confirm suspected COVID-19-related symptoms, after which any positive test result was reported as symptomatic COVID-19.

Enrolled subjects received a single 90 µg dose in 500 μL of AKS-452 *via* subcutaneous injection in the deltoid region of the upper arm. Each subject was observed during a 15 min post-vaccination period before being released. Safety reviews and immunogenicity assessments were scheduled for study days 0, 28, 56, 90 180, and 273, at which times blood samples were obtained for preparations of serum and stored frozen until analysis. An informed consent form was signed voluntarily before any study-related procedure was performed. Subjects were given an emergency call card at the day 0 visit prior to vaccination and instructed to report, in an unsolicited manner, every change in health or well-being after vaccination given on the day 1 visit. After the opportunity to report and discuss unsolicited reactions at this day 1 visit, subjects were given a symptom questionnaire and diary card after which they were instructed to report (*i.e*., solicited) any AEs and SAEs at any time during the trial. During all follow-up appointments, subjects reported any symptoms in an open unsolicited manner followed by a solicited symptom questionnaire discussion. (See details of the clinical study procedures in the *Research Protocol, # 901452-CT-21-001* (see *Supplementary Material*).

### Trial oversight

The trial was reviewed and approved by the Central Committee on Research involving Humans (CCMO) in The Hague, together with a review by the Ministry of Health, Welfare and Sport (VWS). Local feasibility was assessed and approved by the UMCG Institutional Review Board. For release of the sponsor-initiated trial at the clinical site, the UMCG was the site that conducted the study and TRACER BV (The Netherlands) was the contract research organization that managed and was responsible for the entire clinical project on behalf of the sponsor, Akston Biosciences Corporation (USA). The decision to submit the manuscript for publication was made by all authors who vouch for the accuracy and completeness of the reported data and for the fidelity of trial operations to the protocol. No one who is not an author contributed to the preparation of the manuscript.

### Primary and secondary endpoints

The primary endpoint was the determination of the percentage of patients that (i) achieve an SP/RBD-specific IgG titer greater than the validated positive cut-off value of 1.44 µg/mL at day 28 after AKS-452 administration if the pre-dose baseline (day 0) value was less than the cut-off, or (ii) achieve at least a 2-fold increase in titer relative to baseline if the baseline value ≥ 1.44 µg/mL (i.e., *Enhanced Immune Response*). The secondary endpoint was safety evaluation for local and systemic AEs after administration of AKS-452 at each pre-defined scheduled follow-up visit (days 28, 56, 90, 180, and 273). Research objectives included IgG titers against mutant SP/RBD of different SARS-CoV-2 variant strains (*via* enzyme-linked immunosorbent assays, ELISA) and the neutralization of such live virus variants (*via* PRNT), in addition to serum IgG isotyping (via ELISA). AEs and SAEs were graded by a numerical scoring system defined by the *NCI Common Terminology Criteria for Adverse Events* (NCI CTCAE; version number V4.03; i.e., Grade 1, Mild; Grade 2, Moderate; Grade 3, Severe or medically significant but not immediately life threatening; Grade 4, Life threatening consequences; Grade 5, Death related to the adverse event).

### Laboratory analyses

A quantitative anti-SP/RBD IgG titer ELISA (developed at Akston Biosciences, Beverly, MA; see detailed methods published elsewhere^17^) was used to assess titers at baseline (day 0) and on days 28, 56, 90, 180, and 273 post-vaccination in which the cut-off value defining seropositivity was a titer ≥1.44 µg/mL (derived by 4.5 standard deviations above the mean obtained using sera from 80 COVID-19-naïve subjects; *data not shown*). The capacity of anti-SP/RBD antibodies (and associated IgG isotypes) to bind a series of SP/RBD mutant proteins from known SARS-CoV-2 variants and to bind Np of the WT virus was assessed via ELISAs (see detailed methods published elsewhere^17^; the cut-off for positivity of the anti-Np IgG titer was 0.5 µg/mL that reflects prior exposure to SARS-CoV-2). The potency of serum to inhibit binding of recombinant SP/RBD to recombinant ACE2 was expressed as an effective dilution 50% (ED50) value (developed at Akston Biosciences, Beverly, MA) as previously described^16^. A PRNT was performed with VERO E6 cells to define the ED50 and effective dilution 99% (ED99) values via serial dilution of serum starting at 40-fold dilution; the three SARS-CoV-2 viral strains used were the ancestral Washington wild-type (WT) [variant USA-WA1/2020; World Reference Center for Emerging Viruses and Arboviruses, University of Texas Medical Branch, TX, USA; GenBank accession no. MN985325.1], Delta [Lineage B.1.617.2, GenBank accession no. OL442162.1], and Omicron BA.1 [Lineage BA.1.1.529; GenBank accession no. (OP388404.1)]. All ED50 and ED99 values were calculated using the *non-linear regression log(agonist) vs. response* analysis, GraphPad Prism, version 9.0.

### Statistical analysis

As described in the *Research Protocol* (see *Supplementary Material*), statistical derivation of the dosing regimen and number of subjects for this study were based on safety and immunogenicity outcomes of the phase I/II study that included a total of 112 COVID-19-naïve adult subjects who received one or two doses of AKS-452 formulated in the oil-in-water adjuvant, Montanide™ ISA 720, at dose levels 22.5, 45, or 90 µg^17^. Seroconversion rates were typically 100% within each dosing cohort in that study in which the 90 µg dose consistently yielded the highest IgG titers. Safety outcomes from that study demonstrated no AEs > grade 3 and no SAEs due to the vaccine. Considering these outcomes of the phase I/II study, the highest dose level of 90 µg was used in this study to evaluate the non-adjuvanted formulation of AKS-452 as a booster vaccination in subjects already vaccinated with other regulatory-approved vaccines. We also considered an outcome from the phase I/II study that demonstrated a “non-adjuvanted” AKS-452 dose administered 180 days after a single adjuvanted priming dose to five subjects induced a robust response 28 days later (i.e., day 208) in all five subjects, demonstrating the potential of a single non-adjuvanted dose. Although phase I/II immunogenicity data was used to derive statistical power for the 600-subject design of this ACT-BOOSTER study (as described in the study protocol), the amended redesigning of this study to a single cohort of 71 subjects nullified the predetermined statistical plan. Continuous variables were tested for normal distribution and if non-normally distributed, data were log10-transformed to obtain a normal distribution. Immunogenicity data were log-transformed (except for PRNT data) before performing one-way analysis of variance (ANOVA) comparisons of least squares means, as appropriate, using JMP for Windows™ Version 17.1 (JMP Statistical Discovery LLC). Specific statistical analyses and results are described in each figure legend. As a per-protocol criterion, any subject reporting COVID-19 symptoms (and confirmed SARS-CoV-2 infection via rapid antigen testing performed by the subject at home) was allowed to continue in the study.

### Reporting summary

Further information on research design is available in the Nature Research Reporting Summary linked to this article.

### Supplementary information

Supplementary FData

Research Protocol

REPORTING SUMMARY

## Data Availability

The authors declare that the data supporting the findings of this study are available within the paper and its supplementary information files.
