# Supplementary material for: Immunogenicity phase II study evaluating booster capacity of nonadjuvanted AKS-452 SARS-Cov-2 RBD Fc vaccine
Source: NPJ Vaccines. 2024 Feb 21;9:40. doi: 10.1038/s41541-024-00830-2 (PMC10881471; doi:10.1038/s41541-024-00830-2)
Supplement: Supplementary file 1 — Supplementary FData [file 41541_2024_830_MOESM1_ESM.pdf]

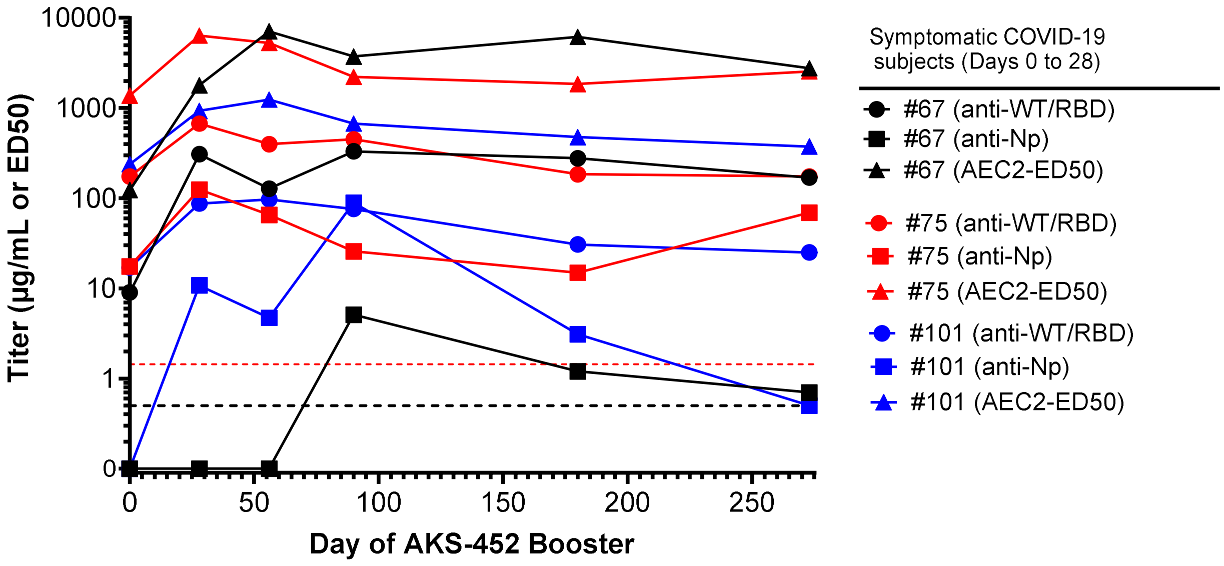

Supplementary Figure 1. Longitudinal Immunogenicity of subjects #67, #75, and #101. Serum samples were obtained from the 3 subjects #67, #75, and #101 (who were COVID-19 symptomatic after day 0 but before the day 28 visit) on days 0, 28, 56, 90, 180, and 273 after receiving a 90 µg s.c. dose of AKS-452 administered ≥ 3 months after completion of regulatory–approved vaccinations and assessed for anti-WT/RBD IgG (µg/mL), anti-Np IgG (µg/mL), and ACE2-WT/RBD binding inhibition (ED50) titers via ELISA. Positive cut-off for anti-RBD IgG was 1.44 µg/mL (dotted redline) and that for anti-Np IgG was 0.5 µg/mL (dotted black line).

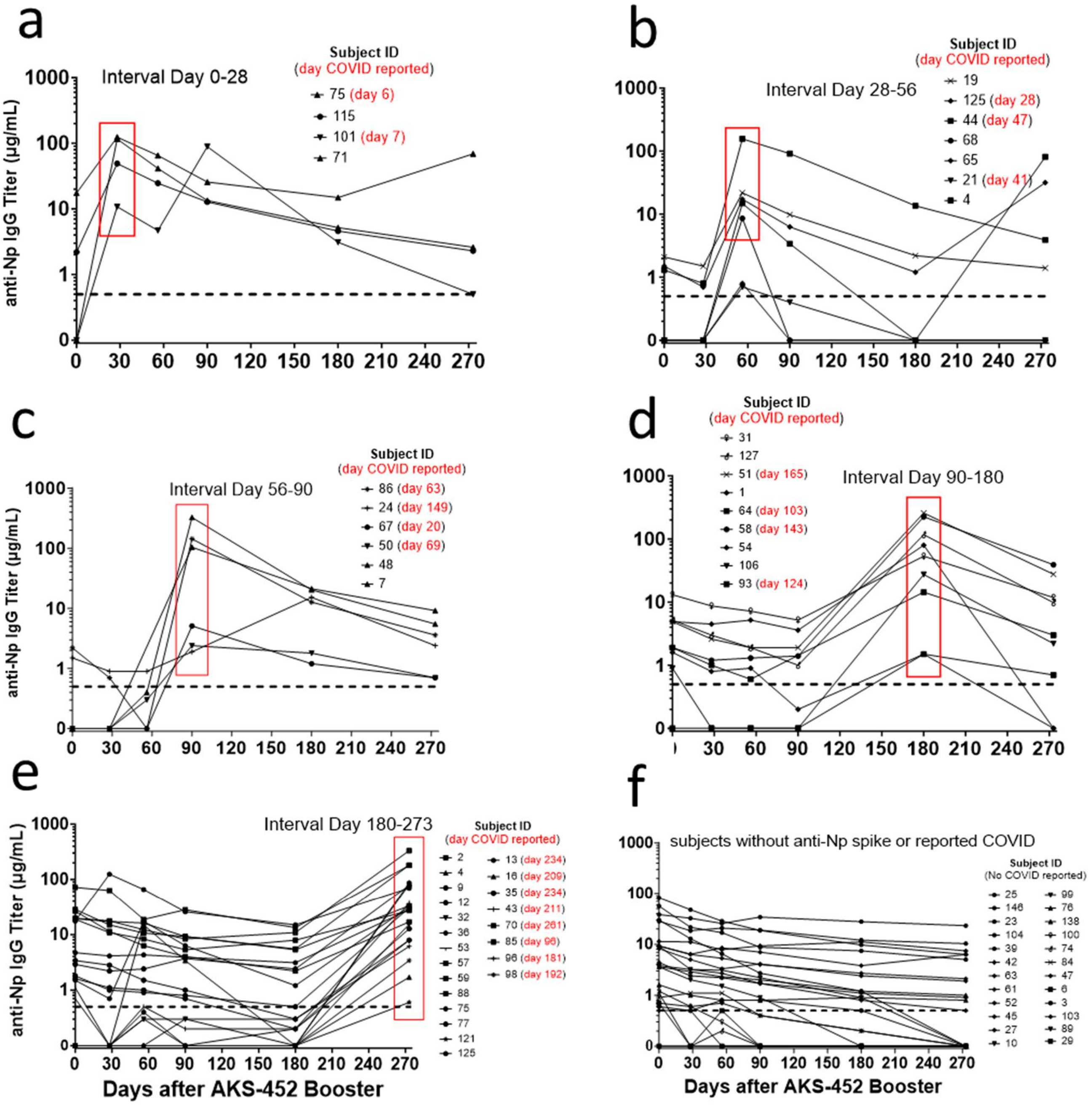

Supplementary Figure 2. Longitudinal Nucleocapsid Protein (Np) IgG titers. Serum samples were obtained from 70 subjects on days 0, 28, 56, 90, 180, and 273 after receiving a 90 µg s.c. dose of AKS-452 administered ≥ 3 months after completion of regulatory–approved vaccinations and assessed for anti-Np IgG binding titers via ELISA of which a positive cut-off was 0.5 µg/mL, dotted line in each panel. Subjects that showed a >2-fold spike increase in anti-Np titers (a-e) are shown per interval during which the spike occurred; i.e., between days 0-28 (a), days 28-56 (b), days 56-90 (c), days 90-180 (d), and days 180-273 (e). Those subjects that reported symptomatic COVID-19 (and confirmed with home rapid antigen test) are denoted via red text in respective panels. Those subjects that had no anti-Np spike or reported symptomatic COVID-19 are represented in panel f.

Supplementary Table 1: Individual AEs of All Subjects in ACT-BOOSTER AKS-452 phase II study

Description of AE	AKS-452 dose	AE Onset	AE End	AE Duration	Day of AE Onset after AKS-452 dose
**AEs Related to AKS-452 dosing**					
**General Symptoms**					
Common cold	5/12/2022	5/14/2022	5/18/2022	4	2
Fatigue	5/25/2022	5/26/2022	6/1/2022	6	0
Fatigue	5/25/2022	5/26/2022	6/6/2022	11	1
Fatigue	5/23/2022	6/8/2022			15
General malaise	5/30/2022	6/1/2022	6/8/2022	7	1
Headache	5/25/2022	5/28/2022	5/31/2022	3	2
Muscle ache	5/25/2022	5/26/2022	5/30/2022	4	1
Papular rash	5/25/2022	6/7/2022	11/16/2022	162	12
**Local Symptoms**					
Injection site pain	5/12/2022	5/13/2022	5/17/2022	4	1
Injection site reaction	5/23/2022	5/24/2022	5/26/2022	2	0
Injection site reaction	6/1/2022	6/1/2022	6/4/2022	3	0
Injection site reaction	6/8/2022	6/9/2022	6/12/2022	3	0
Injection site reaction	5/23/2022	5/24/2022	5/27/2022	3	0
Injection site reaction	5/12/2022	5/12/2022	5/16/2022	4	0
Injection site reaction	5/25/2022	5/26/2022	5/30/2022	4	0
Injection site reaction	6/8/2022	6/9/2022	6/13/2022	4	0
Injection site reaction	6/15/2022	6/16/2022	6/22/2022	6	0
Injection site reaction	5/23/2022	5/24/2022	5/30/2022	6	0
Injection site reaction	5/25/2022	5/25/2022	6/1/2022	7	0
Injection site reaction	5/25/2022	5/25/2022	6/1/2022	7	0
Injection site reaction	5/12/2022	5/12/2022	5/26/2022	14	0
Injection site reaction	5/23/2022	5/24/2022	5/25/2022	1	1
Injection site reaction	5/25/2022	5/26/2022	5/28/2022	2	1
Injection site reaction	6/13/2022	6/14/2022	6/17/2022	3	1
Injection site reaction	5/25/2022	5/26/2022	5/29/2022	3	1
Injection site reaction	5/25/2022	5/26/2022	5/29/2022	3	1
Injection site reaction	6/1/2022	6/2/2022	6/7/2022	5	1
Injection site reaction	6/15/2022	6/16/2022	6/22/2022	6	1
Injection site reaction	5/30/2022	5/31/2022	6/7/2022	7	1
Injection site reaction	5/30/2022	6/1/2022	6/4/2022	3	2
Injection site reaction	5/25/2022	5/27/2022	5/30/2022	3	2
Injection site reaction	5/30/2022	6/1/2022	6/4/2022	3	2
Injection site reaction	5/25/2022	5/27/2022	5/31/2022	4	2
Injection site reaction	6/13/2022	6/15/2022	6/22/2022	7	2
Injection site reaction	6/1/2022	6/4/2022	6/5/2022	1	3
Injection site reaction	5/23/2022	5/26/2022	5/30/2022	4	3

Injection site reaction	5/30/2022	6/2/2022	6/6/2022	4	3
Injection site reaction	6/7/2022	6/10/2022	6/15/2022	5	3
Injection site reaction	6/7/2022	6/10/2022	6/15/2022	5	3
Injection site reaction	5/25/2022	5/28/2022	6/7/2022	10	3
Injection site reaction	5/12/2022	5/17/2022	5/19/2022	2	5
Injection site redness	5/23/2022	5/24/2022	5/27/2022	3	0
Injection site redness	6/13/2022	6/14/2022	6/20/2022	6	0
Injection site redness	5/12/2022	5/13/2022	5/20/2022	7	1
Injection site redness	5/23/2022	5/24/2022	7/2/2022	39	1
Injection site redness	5/23/2022	5/25/2022	5/30/2022	5	2
Injection site redness	5/23/2022	5/26/2022	5/28/2022	2	3
Injection site redness	6/1/2022	6/9/2022	6/13/2022	4	8
Injection site swelling	6/8/2022	6/8/2022	6/11/2022	3	0
Injection site swelling	5/23/2022	5/24/2022	5/27/2022	3	0
Injection site swelling	6/15/2022	6/15/2022	6/20/2022	5	0
Painful arm	5/25/2022	5/25/2022	5/26/2022	1	0
Painful arm	5/25/2022	5/25/2022	5/27/2022	2	0
Painful arm	5/25/2022	5/26/2022	6/1/2022	6	0
Painful arm	6/8/2022	6/12/2022	8/3/2022	52	4

**AEs Unrelated to AKS-452 dosing**					
COVID-19 infection	6/15/2022	6/21/2022	6/26/2022	5	6
COVID-19 infection	6/1/2022	6/8/2022	6/13/2022	5	6
COVID-19 infection	6/1/2022	6/21/2022	6/27/2022	6	20
COVID-19 infection	6/7/2022	7/5/2022	7/12/2022	7	28
COVID-19 infection	5/25/2022	7/5/2022	7/12/2022	7	40
COVID-19 infection	5/25/2022	7/11/2022	7/18/2022	7	47
COVID-19 infection	5/25/2022	7/27/2022	7/31/2022	4	63
COVID-19 infection	6/13/2022	9/17/2022	9/22/2022	5	95
COVID-19 infection	6/1/2022	9/12/2022	9/22/2022	10	103
COVID-19 infection	5/30/2022	10/1/2022	10/22/2022	21	123
COVID-19 infection	5/30/2022	10/20/2022	11/1/2022	12	142
COVID-19 infection	5/23/2022	10/19/2022	10/28/2022	9	149
COVID-19 infection	5/25/2022	11/6/2022	11/21/2022	15	165
COVID-19 infection	5/25/2022	11/23/2022	11/30/2022	7	181
COVID-19 infection	6/8/2022	12/17/2022	12/22/2022	5	192
COVID-19 infection	5/25/2022	12/21/2022	1/11/2023	21	209
COVID-19 infection	5/23/2022	12/20/2022	12/25/2022	5	211
COVID-19 infection	5/25/2022	1/15/2023	1/25/2023	10	234
COVID-19 infection	5/23/2022	1/13/2023	1/19/2023	6	234
COVID-19 infection	5/25/2022	2/10/2023	2/14/2023	4	261
Suspected COVID-19 (not confirmed)	5/12/2022	7/20/2022	7/22/2022	2	69
COVID-19 infection during screen, prior AKS-452	6/15/2022	5/30/2022	6/6/2022	7	-16

Abdominal pain	5/25/2022	11/11/2022	11/21/2022	10	169
Abdominal pain (cause unknown)	5/25/2022	12/28/2022	2/8/2023	42	217
Allergic reaction	5/30/2022	2/16/2023	2/17/2023	1	262
Car accident	6/8/2022	2/24/2023	3/2/2023	6	260
Chlamydia infection	6/1/2022	7/26/2022	8/6/2022	11	54
Chlamydia infection	6/8/2022	6/10/2022	6/12/2022	2	1
Chlamydia infection	6/1/2022	12/2/2022	12/16/2022	14	183
Chlamydial infection	6/7/2022	5/23/2022	5/30/2022	7	-16
Common cold	5/23/2022	6/24/2022	6/30/2022	6	31
Common cold	5/30/2022	6/10/2022	6/15/2022	5	10
Common cold	6/15/2022	11/28/2022			165
Common cold	5/23/2022	7/18/2022	7/25/2022	7	55
Common cold (tested COVID negative)	5/12/2022	6/19/2022	6/26/2022	7	38
Conjunctivitis	5/25/2022	1/18/2023	1/31/2023	13	238
Deviated nasal septum (septum correction)	5/30/2022	7/25/2022	8/15/2022	21	56
Eczema	5/25/2022	11/15/2022			173
Eczema (acute, feet)	5/23/2022	8/25/2022			93
General malaise (headache, cold)	5/23/2022	12/20/2022	12/25/2022	5	210
Headache (fall on the head)	5/30/2022	1/16/2023	1/19/2023	3	230
Headwound because of fall	5/25/2022	7/9/2022	7/18/2022	9	44
Hernia cervicalis	5/23/2022	10/1/2022			131
Laryngitis	6/1/2022	7/6/2022	7/20/2022	14	35
Laryngitis	5/30/2022	7/5/2022	7/19/2022	14	35
Otitis	5/30/2022	2/10/2023			255
Palpitations (heart; cause unknown)	6/1/2022	1/17/2023			229
Pneumonia	5/23/2022	12/1/2022	12/14/2022	13	192
Pregnancy (unplanned)	5/25/2022	10/5/2022			132
Scabies (skin itching)	6/8/2022	11/28/2022	12/5/2022	7	172
Scar-tissue Swelling (shoulder surgery)	5/25/2022	6/17/2022			22
Shoulder pain (from whiplash)	5/23/2022	6/10/2022			18
Shoulder Pain (unknown origin)	5/23/2022	11/18/2022			178
Vasovagal Syncope	5/30/2022	6/27/2022	6/27/2022	0	28
Wound from a sharp rusty object	5/23/2022	5/31/2022	6/2/2022	2	8

Supplementary Table 2: Longitudinal Laboratory Analyses of All Subjects in ACT-BOOSTER AKS-452 phase II

	Screening					Visit 1 (pre-dose)					Visit 2 (day 28)					Visit 3 (day 56)					Visit 4 (day 90)					Visit 5 (day 180)					Visit 6 (day 273)				
Test	N	Min	Max	Mean	SD	N	Min	Max	Mean	SD	N	Min	Max	Mean	SD	N	Min	Max	Mean	SD	N	Min	Max	Mean	SD	N	Min	Max	Mean	SD	N	Min	Max	Mean	SD
ESR (mm/h)	71	1	28	7	7	70	1	22	7	6	69	1	42	7	7	67	1	69	8	10	70	1	30	7	6	69	1	31	8	7	69	1	51	9	9
WBC (10^9/L)	71	3.6	12.5	6.3	1.6	71	3.6	10.3	6.4	1.7	69	3.6	10.9	6.2	1.6	67	3.4	11.4	6.4	1.7	70	3.9	13.1	6.5	1.8	69	4	10.9	6.4	1.6	69	3.8	12.1	7.0	2.0
Hb (mmol/L)	71	7.3	10.9	8.9	0.7	71	6.4	10	8.6	0.8	69	6.4	10	8.6	0.7	67	6.5	9.8	8.6	0.8	70	6.9	10.4	8.7	0.7	69	6.1	10.3	8.6	0.8	69	6	10.4	8.6	0.8
HCT (L/L)	71	0.36	0.51	0.4	0.0	71	0.33	0.48	0.4	0.0	69	0.33	0.49	0.4	0.0	67	0.33	0.48	0.4	0.0	70	0.36	0.49	0.4	0.0	69	0.32	0.5	0.4	0.0	69	0.32	0.49	0.4	0.0
MCV (fl)	71	67.9	100.4	90.1	4.0	71	67.8	101.1	89.6	4.4	69	65.6	101	90.2	4.5	67	67	101	90.4	4.5	70	66.3	103	90.0	4.8	69	67.3	102	90.6	4.8	69	66.8	102	89.8	5.1
Platelets (10^9/L)	71	161	490	271.7	57.5	71	146	461	252.7	54.8	69	158	410	256.8	54.0	67	161	464	268.6	57.0	70	175	474	264.2	58.3	69	169	396	270.6	55.2	69	156	390	278.1	56.3
Neutrofils (10^9/L)	71	1.52	9.69	3.8	1.3	69	1.35	7.49	3.8	1.4	68	1.79	6.99	3.6	1.3	67	1.21	7.55	3.7	1.4	70	1.4	8.73	3.7	1.5	69	1.62	7.57	3.7	1.4	67	1.74	9.15	4.0	1.6
Lymphocytes (10^9/L)	71	1.21	3.3	1.8	0.4	71	1.01	4.29	1.8	0.6	68	0.84	3.27	1.9	0.5	67	1.22	3.55	2.0	0.5	70	0.99	4.31	2.0	0.6	69	1.04	3.07	2.0	0.5	67	1.08	4.49	2.1	0.6
Monocytes (10^9/L)	71	0.22	0.75	0.5	0.1	71	0.19	0.97	0.5	0.1	68	0.19	1.06	0.5	0.1	67	0.25	0.89	0.5	0.1	70	0.22	0.98	0.5	0.1	69	0.2	0.86	0.5	0.1	67	0.23	1.04	0.5	0.1
Eosinophils (10^9/L)	71	0.02	0.42	0.1	0.1	71	0	0.58	0.2	0.1	68	0.02	0.66	0.2	0.1	67	0.04	0.46	0.2	0.1	70	0.01	0.5	0.2	0.1	69	0.03	0.49	0.2	0.1	67	0.02	0.49	0.2	0.1
Basophils (109/L)	71	0.01	0.09	0.0	0.0	71	0.01	0.1	0.0	0.0	68	0.01	0.09	0.0	0.0	67	0.01	0.1	0.0	0.0	70	0.01	0.09	0.0	0.0	69	0.02	0.1	0.0	0.0	67	0.01	0.12	0.1	0.0
CRP (mg/L)	71	0.3	32	2.6	5.8	71	0.3	27	2.2	4.5	70	0.3	106	2.9	12.6	67	0.3	25	2.2	4.1	70	0.3	10	1.4	1.7	70	0.3	17	1.7	2.7	69	0.3	14	2.3	3.0
Sodium (mmol/L)	71	137	143	139.9	1.4	71	137	144	139.8	1.5																									
Potassium (mmol/L)	71	3.4	5	3.9	0.3	71	3.4	4.6	3.9	0.3																									
Creatinin (µmol/L)	71	47	108	70.5	12.2	71	51	109	71.3	12.7																									
Albumin (g/L)	71	40	56	47.2	3.0	71	39	55	46.3	2.9																									
Troponin (g/L)	71	3	13	5.5	2.5	71	3	17	5.6	2.6																									
CK (U/L)	71	39	1775	179.4	240.0	71	34	4657	243.2	585.8																									
LDH (U/L)	71	101	337	190.8	35.8	71	105	545	189.4	52.1																									
ASAT (U/L)	71	13	72	25.8	9.4	71	12	261	27.2	29.5																									
ALAT (U/L)	71	10	52	22.6	9.0	71	9	113	22.3	13.9																									
ALP (U/L)	71	38	116	72.8	16.5	71	37	118	72.0	16.6																									
γGT (U/L)	71	7	57	18.2	9.3	71	7	67	18.6	9.2																									
Bilirubin (µmol/L)	71	3	29	9.7	5.4	71	3	29	8.9	5.4																									
Amylase (U/L)	71	26	174	59.8	22.4	71	22	150	57.3	20.8																									
INR	71	0.9	1.1	1.0	0.1	67	0.9	1.2	1.0	0.1																									
PT (s)	71	10.3	13.5	11.7	0.7	67	10.4	13.7	11.7	0.8																									
APTT (s)	71	22	42	30.9	3.1	67	21	36	30.4	2.8																									
Fibrinogen (g/L)	71	1.8	5	2.8	0.7	66	1.7	4.7	2.7	0.6																									
HBA1c (mmol/mol)	71	25	39	34.3	2.6																														

Supplementary Table 3: Increase in anti-Np IgG titers and onset of COVID-19

Interval Visits End/Baseline	Subject ID	Interval Baseline Np IgG titer (ug/mL)	Interval End Np IgG Titer (ug/mL)	Np IgG Interval Increase[1]	WT/RBD IgG Fold-increase[2]	Day of reported COVID infection[3]
Day 28/day 0	101	0	10.8	10.8*	5.2	7
Day 28/day 0	75	17.5	124.1	7.1	3.9	6
Day 28/day 0	115	2.2	49.2	22.6	1.8	
Day 28/day 0	71	0.0	116.4	*116.4**	8.1	
Day 28/day 0	132	3.2	30.4	9.5	7.1	
Day 56/Day 28	4	0.0	14.9	*14.9**	0.7	
Day 56/Day 28	19	1.5	21.9	14.8	1.9	
Day 56/Day 28	21	0.0	0.7	*0.7**	0.9	41
Day 56/Day 28	44	0.8	156.5	188.8	2.5	47
Day 56/Day 28	65	0.0	0.8	*0.8**	0.1	
Day 56/Day 28	68	0.0	8.6	*8.6**	0.8	
Day 56/Day 28	125	0.7	17.3	23.5	0.7	28
Day 90/Day 56	7	0.4	326.0	739.4	0.8	
Day 90/Day 56	24	0.9	1.9	2.0	0.4	149
Day 90/Day 56	48	0.0	104.3	*104.3**	1.4	
Day 90/Day 56	50	0.3	2.4	6.8	0.8	69†
Day 90/day 56	67	0	5.1	5.1*	2.6	20
Day 90/Day 56	86	0.0	143.5	*143.5**	3.5	63
Day 180/Day 90	1	3.6	79.6	22.3	0.6	
Day 180/Day 90	31	5.2	53.4	10.2	0.7	
Day 180/Day 90	51	1.9	257.5	136.4	0.9	165
Day 180/Day 90	54	0.2	1.5	6.2	0.5	
Day 180/Day 90	58	1.4	225.4	162.0	0.7	143
Day 180/Day 90	64	0.0	14.4	*14.4**	0.6	103
Day 180/Day 90	93	0.0	1.5	*1.5**	1.2	124
Day 180/Day 90	106	0.0	27.4	*27.4**	0.7	
Day 180/Day 90	127	1.0	117.3	121.3	2.7	
Day 273/Day 180	2	10.8	332.6	30.9	1.0	
Day 273/Day 180	4**	0.1	81.1	1303.5	1.5	
Day 273/Day 180	9	2.4	77.9	32.5	3.8	
Day 273/Day 180	12	0.2	12.7	79.1	0.6	
Day 273/Day 180	13	3.1	27.9	9.1	2.2	234
Day 273/Day 180	16	0.0	1.7	1.7*	1.9	209
Day 273/Day 180	32	0.0	15.4	15.4*	3.1	
Day 273/Day 180	35	0.5	30.2	60.9	0.6	234
Day 273/Day 180	36	0.0	86.6	86.6*	4.6	
Day 273/Day 180	43	0.2	37.0	235.9	0.9	211
Day 273/Day 180	53	0.2	7.9	49.4	1.3	
Day 273/Day 180	57	13.3	179.5	13.5	0.8	
Day 273/Day 180	59	2.2	16.9	7.7	1.7	
Day 273/Day 180	70	5.3	32.9	6.2	1.7	261

Day 273/Day 180	77	0.3	7.9	28.1	2.8	
Day 273/Day 180	85	7.9	27.0	3.4	1.1	96
Day 273/Day 180	88	5.5	181.3	33.0	1.1	
Day 273/Day 180	96	0.3	6.1	19.6	1.4	181
Day 273/Day 180	98	0.0	3.4	3.4*	0.9	192
Day 273/Day 180	121	0.0	0.6	0.6*	4.1	
Day 273/Day 180	125**	1.2	31.7	27.3	0.9	
Day 273/Day 180	75**	1.2	69.2	55.5	0.0	

[1] Values reflect either the fold-increase over baseline values or "0" baseline value subtracted (*)
[2] Fold-change during anti-Np IgG change interval
[3] COVID-19 symptoms confirmed via Rapid SP-Antigen Test
** Showed an anti-Np titer increase and/or COVID-19 symptomatic incident in a previous interval as well
† COVID not confirmed via test

Supplementary Table 4. AKS-452 Drug Substance Characterization (Batch #MDS0006)

Test	Result
**Strength**	3290 µg/mL (BCA)
**pH**	7.3
**Osmolality**	314 mOsm/kg
**Appearance**	Clear, colorless (non-turbid)
**Endotoxin**	< 1.60 EU/mL
**Bioburden**	< 1 CFU/mL
**Identity**	Consistent with Reference Standard
**Aggregate**	< LLOQ (< 1.1%)
**Fragment**	< LLOQ (< 1.7%)
**CHO Host Cell Protein**	8 ppm
**CHO Host Cell DNA**	< LLOQ (< 6.08 ppb)
**Residual Protein A**	0.40 ppm
**Potency (Report Results)**	731 pM (EC50 ≤ Potency Control)
**cIEF – Report pI**	8.1
**Elemental Impurities**	µg/200µL dose:
**Cd**	0.001
**Pb**	0.002
**As**	0.008
**Hg**	0.01
**Co**	None Detected
**V**	0.007
**Ni**	None Detected
**Li**	0.2
**Sb**	0.04
**Cu**	None Detected
**Glycan Profile**	
**Man5**	4.70%
**G0**	7.80%
**G0F**	26.20%
**G1**	4.10%
**G1F**	9.10%
**G1'F**	8.80%
**G2**	3.50%
**G2F**	15.20%
**Other**	20.60%

Supplementary Table 5. AKS-452 Drug Product Characterization (Batch #TGR20644/AKS452X/10JUN21)

Test	Result
**Strength**	3,147 µg/mL (BCA)
**pH**	7.4
**Osmolality**	298 mOsm/kg
**Appearance**	Complies with Clear to Moderately Opalescent, Colorless to Light Yellow Solution
**Endotoxin**	< 2.00 EU/mL
**Sterility**	No Visible Growth
**Identity**	Complies with Consistent with Reference Standard
**Aggregate**	1.5%
**Fragment**	< LLOQ (< 1.8%)
**Potency (Report Results)**	543 pM (EC50 ≤ Potency Control)
**cIEF – Report pI**	8.13
**Particulate Matter**	≥ 10µm: 387 / Container<br>≥ 25µm: 43 / Container
**Extractable Volume**	84% of Container Labeled Fill Volume

Supplementary Table 6. 6- and 12-month stability results conducted at -80°C, 5°C, and 25°C for AKS-452 Drug Product (Batch #TGR20644/AKS452X/10JUN21 formulated from drug substance Batch #MDS0006)

	Acceptance Criteria	Time 0	6 months			12 months		
			-80°C	5°C	25°C	-80°C	5°C	25°C
**BCA (µg/mL)**	2500 – 3500	3200	3120	3160	3210	3260	3090	3150
**pH**	6.8 – 7.6	7.4	7.3	7.3	7.3	7.4	7.3	7.4
**Aggregate (%)**	≤ 20%	2.1%	1.8%	1.9%	3.5%	1.8%	2.2%	4.7%
**Fragment (%)**	≤ 25%	< LLOQ (< 2.4%)	< LLOQ (< 2.5%)	< LLOQ (< 2.6%)	6.1%	Pending	Pending	Pending
**Potency (EC50, pM)**	Report EC50 (pM)	728	1040	1060	1020	871	657	1010
**Potency (≤ Potency Control)**	EC50 ≤ Potency Control	PASS	PASS	PASS	PASS	PASS	PASS	PASS
**cIEF**	Report Peak Area Weighted pI	8.13	8.13	8.05	7.62	8.16	8.07	7.43
	Visually Consistent with Time 0	N/A	PASS	PASS	PASS	PASS	PASS	FAIL
**Appearance**	Clear to moderately opalescent; colorless to light yellow solution	Clear, colorless solution	Slightly opalescent, light-yellow solution*			Clear, light-yellow solution*		

* Due to change in analysis method between Time 0- and 6-month timepoints
