# Supplementary material for: Immunogenicity phase II study evaluating booster capacity of nonadjuvanted AKS-452 SARS-Cov-2 RBD Fc vaccine
Source: NPJ Vaccines. 2024 Feb 21;9:40. doi: 10.1038/s41541-024-00830-2 (PMC10881471; doi:10.1038/s41541-024-00830-2)
Supplement: Supplementary file 2 — Research Protocol [file 41541_2024_830_MOESM2_ESM.pdf]

RESEARCH PROTOCOL

Anti-COVID19 VaccinaTion AKS-452X BOOSTER Study

(ACT-BOOSTER study)

**Protocol ID**	**901452-CT-21-001**
**Short title**	**ACT-BOOSTER Study**
**EudraCT number**	**2021-005509-28 / ABR 79397**
**Version**	**4.0**
**Date**	**January 14th, 2022**
**Coordinating investigator/project leader**	**Prof Dr G.M. van Dam, MD, PhD**<br>**TRACER Europe BV**<br>**L.J. Zielstraweg 1, 9713 GX Groningen**<br>**The Netherlands**<br>**E-mail: go@tracercro.com**<br>**Tel.: +31-6-22914614**
**Principal investigator(s) (in Dutch: hoofdonderzoeker/ uitvoerder)**	**Prof Dr S. Kruijff, MD, PhD**<br>**Department of Surgery**<br>**University Medical Center Groningen**<br>**Hanzeplein 1, 9700 RB Groningen**<br>**The Netherlands**<br>**E-mail: s.kruijff@umcg.nl**
**Sponsor (in Dutch: verrichter/opdrachtgever)**	**Akston Biosciences Corporation**<br>**100 Cummings Center, Suite 454C**<br>**Beverly, MA 01915 USA**
**Independent expert (s)**	**Prof Dr van Baarle, vaccinologist**<br>**Department of Medical Microbiology**<br>**Hanzeplein 1**<br>**9700 RB Groningen, The Netherlands**<br><br>**Dr M.W.N. Nijsten, MD, PhD, internist-intensivist**<br>**Department of Intensive Care**<br>**Hanzeplein 1, 9700 RB Groningen**<br>**The Netherlands**

	**Prof dr H.G.M. Niesters**<br>**Department of Medical Microbiology, section clinical virology**<br>**Hanzeplein 1, 9700 RB Groningen**<br>**The Netherlands**

Laboratory sites	***Akston Biosciences Corp***<br>**100 Cummings Center, Suite 454C**<br>**Beverly, MA 01915**<br>**USA**<br><br>**PRA Health Sciences**<br>**Van Swietenlaan 6, 9728 NZ Groningen**<br>**The Netherlands**<br><br>**University Medical Center Groningen.**<br>**Hanzeplein 1, 9700 RB Groningen**<br>**The Netherlands**
Pharmacy	**PRA Health Sciences**<br>**Van Swietenlaan 6, 9728 NZ Groningen**<br>**The Netherlands**<br><br>**University Medical Center Groningen**<br>**Hanzeplein 1, 9700 RB Groningen**<br>**The Netherlands**

PROTOCOL SIGNATURE SHEET

Name	Signature	Date
**Sponsor or legal representative / Head of Department:**	**Prof Dr R.A.J.O. Dierckx, Head Medical Imaging Center**<br>**Department of Nuclear Medicine and Molecular Imaging**	January 14th, 2022
**[Coordinating Investigator/Project leader/Principal Investigator]:**	**Project Leader:**<br>**Prof Dr GM van Dam, MD, PhD**	January 14th, 2022
	**Principal Investigator:**<br>**Prof Dr S. Kruijff, MD, PhD**	January 14th, 2022
	**Co-investigator:**<br>**Dr H.H. Boersma, PharmD, PhD**	January 14th, 2022

TABLE OF CONTENTS

1. INTRODUCTION AND RATIONALE 11
1.1 COVID-19 overview and the SARS-CoV-2 vaccine landscape 11
1.2 Akston's SP/RBD subunit vaccine rationale 14
1.3 Pre-clinical experience 19
1.3.1 Summary of pre-clinical immunogenicity studies 19
1.5 Clinical Experience / DSMB Report 23
2. OBJECTIVES 23
2.1 Primary Objective: 23
2.2 Secondary Objectives 23
3. STUDY DESIGN 24
4. STUDY POPULATION 30
4.1 Population (base) 30
4.2 Inclusion criteria 30
4.3 Exclusion criteria 31
4.4 Sample size calculation 32
4.5 Investigational product/treatment 35
4.6 Use of co-intervention (if applicable) 35
4.7 Escape medication (if applicable) 35
5. INVESTIGATIONAL PRODUCT 36
5.1 Name and description of investigational product(s) 36
5.2 Summary of findings from non-clinical studies 36
5.3 Summary of findings from clinical studies 36
5.4 Summary of known and potential risks and benefits 36
5.5 Description and justification of route of administration and dosage 37
5.6 Dosages, dosage modifications and method of administration 37
5.7 Preparation and labelling of Investigational Medicinal Product 37
5.8 Drug accountability 37
6. NON-INVESTIGATIONAL PRODUCT 39
6.1 Name and description of non-investigational product(s) 39
6.2 Summary of findings from non-clinical studies 39
6.3 Summary of findings from clinical studies 39
6.4 Summary of known and potential risks and benefits 39
6.5 Description and justification of route of administration and dosage 39
6.6 Dosages, dosage modifications and method of administration 39
6.7 Preparation and labelling of Non-Investigational Medicinal Product 39
6.8 Drug accountability 39
7. METHODS 40
7.1 Study parameters/endpoints 40
7.1.1 Main study parameter/endpoint 40
7.1.2 Secondary study parameters/endpoints 40
7.1.3 Other study parameters (if applicable) 40
7.2 Randomisation, blinding and treatment allocation 40
7.3 Study procedures phase I (including booster with naked AKS-452) 40
7.4 Withdrawal of individual subjects 42
7.4.1 Specific criteria for withdrawal (if applicable): 42
7.5 Replacement of individual subjects after withdrawal 42
7.6 Follow-up of subjects withdrawn from treatment 42

7.7 . Premature termination of the study 42
8. SAFETY REPORTING 44
8.1 Temporary halt for reasons of subject safety 44
8.2 AEs, SAEs and SUSARs 44
8.2.1 Adverse events (AEs) 44
8.2.2 Serious adverse events (SAEs) 45
8.2.3 Suspected unexpected serious adverse reactions (SUSARs) 45
8.3 Annual safety report 46
8.4 Follow-up of adverse events 46
8.5 Data Safety Monitoring Board (DSMB) / Safety Committee 47
9. STATISTICAL ANALYSIS 48
9.1 Analysis Populations 48
9.2 Descriptive statistics 48
9.3 Primary study parameter(s) 48
9.4 Secondary study parameter(s) 49
9.5 Other study parameters 49
9.6 Interim analysis (if applicable) 49
10. ETHICAL CONSIDERATIONS 50
10.1 Regulation statement 50
10.2 Recruitment and consent 50
10.3 Objection by minors or incapacitated subjects (if applicable) 51
10.4 Benefits and risks assessment, group relatedness 51
10.5 Compensation for injury 51
10.6 Incentives (if applicable) 51
11. ADMINISTRATIVE ASPECTS, MONITORING AND PUBLICATION 52
11.1 Handling and storage of data and documents 52
11.2 Monitoring and Quality Assurance 52
11.3 Amendments 52
11.4 Annual progress report 53
11.5 Temporary halt and (prematurely) end of study report 53
11.6 Public disclosure and publication policy 53
12. STRUCTURED RISK ANALYSIS 54
12.1 Potential issues of concern 54
12.2 Synthesis 58
13. REFERENCES 59

LIST OF ABBREVIATIONS AND RELEVANT DEFINITIONS

**ABR**	**General Assessment and Registration form (ABR form), the application form that is required for submission to the accredited Ethics Committee; in Dutch: Algemeen Beoordelings- en Registratieformulier (ABR-formulier)**
**AE**	**Adverse Event**
**AR**	**Adverse Reaction**
**CA**	**Competent Authority**
**CCMO**	**Central Committee on Research Involving Human Subjects; in Dutch: Centrale Commissie Mensgebonden Onderzoek**
**CV**	**Curriculum Vitae**
**DSMB**	**Data Safety Monitoring Board**
**EU**	**European Union**
**EudraCT**	**European drug regulatory affairs Clinical Trials**
**GCP**	**Good Clinical Practice**
**GDPR**	**General Data Protection Regulation; in Dutch: Algemene Verordening Gegevensbescherming (AVG)**
**IB**	**Investigator's Brochure**
**IC**	**Informed Consent**
**IMP**	**Investigational Medicinal Product**
**IMPD**	**Investigational Medicinal Product Dossier**
**METC**	**Medical research ethics committee (MREC); in Dutch: medisch-ethische toetsingscommissie (METC)**
**(S)AE**	**(Serious) Adverse Event**
**SPC**	**Summary of Product Characteristics; in Dutch: officiële productinformatie IB1-tekst**
**Sponsor**	**The sponsor is the party that commissions the organisation or performance of the research, for example a pharmaceutical company, academic hospital, scientific organisation or investigator. A party that provides funding for a study but does not commission it is not regarded as the sponsor, but referred to as a subsidising party.**
**SUSAR**	**Suspected Unexpected Serious Adverse Reaction**
**UAVG**	**Dutch Act on Implementation of the General Data Protection Regulation; in Dutch: Uitvoeringswet AVG**
**WMO**	**Medical Research Involving Human Subjects Act; in Dutch: Wet Medisch-wetenschappelijk Onderzoek met Mensen**

SUMMARY

Rationale: Every decade in the twenty-first century has experienced a new major coronavirus epidemic; SARS in the 2000s, MERS in the 2010s, and now (2020 and onwards) Coronavirus Disease 2019 (COVID-19) caused by the SARS-COV-2 virus. This novel COVID-19 is a severe and acute respiratory illness caused by infection with the SARS-CoV-2 virus. The first COVID-19 case was reported in Wuhan, China in December 2019 and as of October 5th, 2021 there has been approximately 236 million (M) cases world-wide to date (quantified as SARS-Cov-2 virus confirmed and unconfirmed "probable"), in which there are around 4.8 M fatal cases attributed to COVID-19 (*COVID-19 Dashboard by the Center for Systems Science and Engineering (CSSE) at Johns Hopkins University*; https://www.covidtracker.com/). Consequently, to address this pandemic crisis, there is an immediate need for solutions that can accurately quantify the level of neutralizing anti-SARS-CoV-2 antibodies (Abs) in individuals and therapeutically induce and/or amplify the level of neutralizing anti-SARS-CoV-2 Abs across the population. The expectation of the foreseeable future is that natural and vaccine-induced immunity most likely will not be long-lived [4, 7-10], and therefore a cost-effective and safe vaccine administered as frequently as every 6 months (boostering), if necessary, is required to maintain robust immunity among the population. Due to the apparent increased transmissibility of SARS-CoV-2, a global security priority is to advance and stockpile coronavirus vaccines as quickly as possible, inevitably requiring significant international funding and relaxing of regulatory paths in a responsible manner. Given the challenges of a recombinant SARS-Cov-2 Spike Protein (SP) subunit vaccine to induce a strong protective immune response in an immunologically naïve human population, the SP Ag must be modified and/or formulated with additional immune-enhancing features to overcome the activation thresholds of naïve T and B cells. Akston has implemented the following features into its COVID-19 vaccine that are major advantages over most other such vaccines in development, in which the Therapeutic Product Profile (TPP) describes details of its clinical candidate, AKS-452:

1. The use of the smaller focused antigenic portion of SP, the RBD
2. Recombinant fusion of RBD with human IgG1 Fc (SP/RBD-Fc)
3. Emulsification of SP/RBD-Fc in the water-in-oil adjuvant, Montanide ISA 720 (only primary vaccine – which is not part of the booster vaccine used in this study)

In summary, the Fc moiety on AKS-452 is designed to act as a mild adjuvant via inducing activation signaling to the antigen-presenting cell (APC) via FcγRs to enhance the duration of Ag exposure to APCs and perhaps direct Ag entry into lymph nodes locally and systemically where additional APCs reside. As a consequence, the Fc moiety is expected to create a dramatic dose-sparing potential for both the Ag such that the risk of reactogenicity (a safety concern) is dramatically reduced; i.e., too much adjuvant that over-activates many APCs and other innate immune cells can lead a systemic inflammatory reaction termed *reactogenicity*. Such reactogenicity is induced acutely after injection and is not mediated by T and B cells.

Aim: To investigate if a subcutaneous (s.c.) booster dose of 90 µg of the concentrated naked Akston AKS-452 vaccine (AKS-452X) at ≥ 3 months post initial vaccination, with any of the four registered vaccines, will boost the antibody titer and immune response in human healthy volunteers 4-6 weeks after s.c. injection.

Hypothesis: A booster dose of naked (i.e. non-adjuvanted) AKS-452 vaccine will provide an enhanced immune response after vaccination with any of the registered vaccines against COVID-19.

Primary objective: To determine the immunogenicity 4-6 weeks after subcutaneous injection of a booster dose of AKS-452X vaccine given at ≥3 months post-initial vaccination (i.e. Pfizer [Comirnaty], Moderna [Spikevax], Janssen [Ad26.COV2.S], AstraZeneca [Vaxzevria]) in human healthy volunteers.

Secondary objective: Vaccine safety and side effects after booster vaccination. Follow-up will occur for up to 9 months post-study vaccine.

Study design: Single center, open-label, safety and efficacy study on the biological activity of a SP/RBD-Fc antigen booster vaccine (AKS-452X) against COVID-19.

Study population: Healthy human volunteers, 18 - 85 years, having received a registered vaccine (i.e. Pfizer [Comirnaty], Moderna [Spikevax], Janssen [Ad26.COV2.S], AstraZeneca [Vaxzevria]).

Intervention: One booster dose-level of naked AKS-452 (90 µg) administered via s.c. route in 150 subjects per cohort in which safety parameters and neutralizing IgG titers will be reviewed after the booster dose of 90 µg s.c.. Enhanced immune response is defined as: i) seroconversion based on a true positive based on the SP/RBD IgG ELISA assay positive/negative cutoff criteria using the quantitative cut-off value defined by the assay kit batch expressed in µg/mL. The positive/negative cutoff value was established as 2.42 µg/mL from the validation analysis for the current lot of assay kits, but it should be noted that for each new lot of assay kits, Akston QC performs a re-validation of the cutoff value in order to maintain clinical agreement from lot-to-lot, ii) two times (2x) the baseline SP/RBD IgG at day 56 after a boostering, as compared to the titer at the time of screening.

Main study parameters/endpoints: Primary endpoint: The percentage of patients that i) achieve an SP/RBD-specific IgG antibody titer level of ≥ 2.42 µg/mL at the day 28 time-point post-intervention (i.e. booster vaccine) if the base-line value prior to receiving the booster vaccine was < 2.42 µg/mL or ii) where the SP/RBD-specific IgG antibody titer is at least 2x the base-line value prior to receiving the booster vaccine if the base-line value prior to receiving the booster vaccine was ≥ 2.42 µg/mL. The percentage of patients in each of the four cohorts that achieve the primary endpoint threshold at 28 days post-intervention will be calculated (n (%)). **Secondary endpoint:** Safety evaluation in the four cohorts for local and systemic adverse events after injection at each pre-defined scheduled follow-up (at 28, 56, 91, 182 and 273 days

post intervention). Patients will continue to be followed passively for additional safety events out to 9 months post-intervention.

Nature and extent of the burden and risks associated with participation, benefit and group relatedness: The burden of participating in the study will be the number of site visits and possible travelling for subjects, study investigations such as blood samples for measurement of immunogenicity, physical examination prior to inclusion / exclusion, and physical discomfort related to the subcutaneous injection of AKS-452X. The majority of AEs associated with exposure to the AKS-452X vaccine, based on the observations of a first in-human phase I/II clinical study using the same AKS-452 vaccine and adjuvant Montanide ISA-720 (*ClinicalTrials.gov: NCT04681092*) are 'injection site reaction,' and 'injection site nodule'. All registered AEs are likely to subside within days to weeks after appearance. The future benefit, in the case of a safe and sufficient immunogenicity provoking booster vaccine, is for protecting health care workers, future vulnerable and frail elderly, and patients undergoing large surgical procedures for instance oncology, transplantation etc. Moreover, providing protection in co-morbid citizens (i.e., diabetes, overweight, cardiovascular disease, etc.) and ultimately, creating another leverage to returning societies back to their previous health care system, i.e. low- and middle income countries, capacities and economic growth world-wide.

1. INTRODUCTION AND RATIONALE

1.1 COVID-19 overview and the SARS-CoV-2 vaccine landscape

Every decade in the twenty-first century has experienced a new major coronavirus epidemic; SARS in the 2000s, MERS in the 2010s, and now (in 2020) Coronavirus Disease 2019 (COVID-19) caused by the SARS-COV-2 virus. This novel COVID-19 is a severe and acute respiratory illness caused by infection with the SARS-CoV-2 virus. The first COVID-19 case was reported in Wuhan, China in December 2019 and as of October 5th 2021, there has been approximately 236 million (M) cases world-wide to date (quantified as SARS-Cov-2 virus confirmed and unconfirmed "probable"), in which there are 4.8M fatal cases attributed to COVID-19 (*COVID-19 Dashboard by the Center for Systems Science and Engineering (CSSE) at Johns Hopkins University*; https://www.covidtracker.com/). At this time, there are four specific vaccines approved for COVID-19 (i.e. Pfizer [Comirnaty], Moderna [Spikevax], Janssen [Ad26.COV2.S], AstraZeneca [Vaxzevria]). Given the insufficient natural immunity to COVID-19 among the world population, the high basic reproduction number (R0 - R naught) of at least 2.2, the high probability of asymptomatic transmission, and the high death rates estimated at 2-10 times higher than that of influenza, extreme social distancing and widespread shutdown of human activity and interactivity are the only solutions currently available to prevent the spread of infections and minimize the number of serious illnesses and fatalities (reviewed in [1]). The consensus among experts is that society cannot return to normal unless and until there is a sufficient level of immunity conferred on the population worldwide. Achieving natural herd immunity is estimated to require at least 70% of the population to have been infected [1] which would result in millions of deaths worldwide, an ethically unacceptable outcome. Furthermore, early surveillance studies using Ab testing has shown that over 20% of the population in some areas, such as New York City, have antibodies (Abs) to SARS-CoV-2 [2]. However, within that group, many in the population appeared to have borderline to low levels of IgG titers and at present public health officials are unsure of what levels or class of Abs confer neutralizing activity or long-lasting protection against the SARS-CoV-2 virus [3]. Likewise, a more recent screening of 365,000 adults in the general population of England (the *REACT2* study) identified 17,500 that were positive for SARS-Cov-2 Ab titers (i.e., 6% of the general adult population), in which the prevalence of these titer-positive subjects rapidly declined by 27% at 3 months after recovery from infection [4]. These results, combined with early reports from South Korea that some recovered COVID-19 patients have re-presented with measurable SARS-CoV-2 viral loads [5], raises the following key questions: i) what percentage of individuals infected with SARS-CoV-2 develop appreciable levels of Abs, ii) how long do these Abs to SARS-CoV-2 remain in the body after infection, and iii) for how long do they prevent reinfection, if at all? [6]. Consequently, to address this pandemic crisis, there is an immediate need for solutions that can accurately quantify the level of neutralizing anti-SARS-CoV-2 Abs in individuals and therapeutically induce and/or amplify the level of neutralizing anti-SARS-CoV-2 Abs across the population.

The expectation of the foreseeable future is that natural and vaccine-induced immunity most likely will not be long-lived [4, 7-10], and therefore a cost-effective and safe vaccine administered as frequently as every 6 months also as a booster, if necessary, is required to

maintain robust immunity among the population. Due to the apparent increased transmissibility of SARS-CoV-2, a global security priority is to advance and stockpile coronavirus vaccines as quickly as possible, inevitably requiring significant international funding and relaxing of regulatory paths in a responsible manner. Indeed, regulatory agencies appear to embrace this COVID-19 crisis with a unique urgency to allow for an abbreviated preclinical and clinical timeline. A SARS-CoV-2 vaccine could serve as a pan-coronavirus vaccine and appears scientifically feasible if modelled after those previously developed for SARS (caused by the SARS-CoV virus) because both coronaviruses bind the same host target protein, angiotensin converting enzyme 2 (ACE2), expressed in human lung epithelium, endothelial cells, and neuronal cells [11, 12], both exhibit genomes of approximately 30 kb, and SARS-CoV-2 exhibits approximately 89% nucleotide similarly to SARS-like.

Some, but not all, of these features are being implemented in over 170 SARS-CoV-2 vaccine candidates currently in development, including live viruses, nucleic acids, and recombinant protein subunits that may ultimately offer promise as preventive vaccines against COVID-19. However, each vaccine strategy has unique advantages and challenges with respect to manufacturing, safety, and efficacy that must be simultaneously managed in an optimal manner during investigational new drug (IND)-enabling studies such that registration trials can be achieved in a timely manner [14-18]. The following is a summary of the advantages and disadvantages of the current COVID-19 vaccine programs that are most advanced in clinical development.

1. <u>Live-attenuated, inactive whole virus or non-SARS-CoV-2 viral vector-based vaccines</u> represent a classic group of strategies. One viral vector-based approach, which is being deployed by Johnson & Johnson, uses Janssen's AdVac® adenoviral vector that is manufactured in a PER.C6® cell line technology to generate their lead vaccine, JNJ-78436735 [Ad26.COV2.S]. Similarly, AstraZeneca with Oxford University have developed their AZD-1222 vaccine produced using a chimpanzee adenoviral vector, ChAdOx [Vaxzevria] [19, 20]. Both are registered, but have encountered significant serious adverse events (SAEs) that have caused clinical trial pauses in the past [21]. A major advantage of whole virus vaccines is their inherent immunogenicity and ability to stimulate toll-like receptors (TLRs) including TLR 3, TLR 7/8, and TLR 9. However, live virus vaccines often require extensive additional testing to confirm their safety. This is especially an issue for coronavirus vaccines, given the findings of increased infectivity (see below: Antibody Dependent Enhancement, ADE) following immunization with live or killed whole virus SARS coronavirus vaccines [16]. In addition, it is well documented that viral vector vaccines, especially those composed of adenoviruses, induce humor immunogenicity to the vector leading to neutralization of the vaccine itself although use of chimpanzee tropic adenoviruses has temporarily overcome such concerns [22]. Another challenge with whole virus vaccines is the relatively low manufacturability throughput (and therefore COGs) due to either chicken egg-based production or cell expression systems [22].
2. <u>Nucleic acid expression vector vaccine platforms</u> for COVID-19 encode the major coronavirus target antigen (Ag), the Spike Protein (SP), that mediates the virus' infective mechanism via its binding the host receptor, ACE2. ACE2 is expressed on

lung epithelium, blood vessel endothelium, and specific neuronal cells that appears to account for the dominant clinical manifestations of COVID-19, including pulmonary, cardiovascular, and neurological complications, respectively [26]. The major biotech/pharmaceutical companies that have advanced such vaccines to phase 3 trials, include mRNA vaccines encoding the full-length SP developed by BioNTech/Pfizer, BNT162b2 [Comirnaty] [23, 24], and Moderna Therapeutics, mRNA-1273 [Spikevax] [25, 26]. Indeed, both vaccines have reported very positive phase 3 results with efficacy in protecting from symptomatic SARS-CoV-2 viral infection of >90% [27, 28]. Both companies have gained regulatory approval in the Unites States and the European Union. The concept of immunizing with RNA or DNA began with promising results in mice in 1993 showing protective immunity against influenza, but for decades, these findings have not translated to similar findings in humans. Moreover, while non-replicative, many of these RNA and DNA expression vector vaccines continue to endogenously produce the target viral Ag well after induction of the intended immune response, an aspect that could ultimately create immune tolerance to the virus which is a growing concern and may become a practical risk with such current COVID-19 vaccine. Other challenges of these nucleic acid vaccines are the low durability of the response that may require too frequent dosing, and an unfavorable COGs due to cumbersome manufacturability via chemical synthesis.

3. Recombinant subunit vaccines for both SARS coronaviruses rely on eliciting an immune response against the SP to prevent its docking with the host target protein, ACE2 [16, 29, 30]. Novavax has developed and produced immunogenic virus-like nanoparticles based on recombinant expression of SP, NVX-Cov2373, that is formulated with a saponin-based adjuvant system, Matrix-M™, while Clover Biopharmaceuticals is developing a subunit vaccine consisting of a trimerized SARS-CoV-2 SP using their patented Trimer-Tag® technology [31]. Note that the full-length SP target Ag is known to have low expression yields in cell-expression systems and when used in SARS vaccines is known to induce anti-SP IgG titers against non-neutralizing epitopes of SP that mediate increased viral infectivity (i.e., ADE) and inflammation caused by lung eosinophilia (i.e., Th2-mediated immunopotentiation, discussed below). Therefore, a consortium led by Texas Children's Hospital Center for Vaccine Development at Baylor College of Medicine has developed and tested a subunit vaccine comprised of only the receptor-binding domain (RBD) of the SARS SP [16], and when formulated with alum, this RBD-based vaccine can elicit high levels of protective immunity upon homologous virus challenge, in addition to avoiding ADE and immunopotentiation [16]. Initial findings that the SARS and SARS-CoV-2 RBDs exhibit more than 80% amino acid similarity and bind the same ACE2 target offer an opportunity to develop either protein Ag as a subunit vaccine. Indeed, such a subunit vaccine proof-of-concept has been successfully demonstrated with coronavirus SP/RBD Ag's of MERS and SARS infections [18, 29, 32, 33].

1.2 Akston's SP/RBD subunit vaccine rationale

As discussed above, the recombinant protein-based subunit vaccine approach has an advantage of safety and multiple-booster dosing relative to inactivated or live-attenuated virus and nucleic acid vector-based vaccine formats, in addition to allowing for the selective use of the most dominant epitopes to generate potent neutralizing Ab titers [15, 17]. However, the relatively smaller size of the recombinant proteins may pose a problem of lower immunogenicity compared to a whole virus Ag, and therefore require additional features to enhance immunogenicity. The following is a brief discussion of the immunological mechanisms that form the basis of Akston's approach for developing its *immune-enhanced* recombinant subunit vaccine, AKS-452, and emphasizes its distinguishing factors from other closely related vaccine programs.

With respect to a basic immune response, injection of any protein an Ag can, and most likely will, induce an immune response, the magnitude and type of which is highly dependent on the "status" of the respective immune system. For example, injection of a foreign Ag relative to a self Ag will induce a greater immune response in an immune system that maintains central and peripheral tolerance mechanisms, while self Ag can elicit significant immune responses in an immune system with broken tolerance mechanisms, such as an autoimmune condition. Moreover, foreign or self Ag administration to an immune system that has been primed to previous exposure to the respective Ag (e.g., a viral infection or an autoimmune disease) will lodge a more rapid and elevated immune response relative to that of an Ag-naïve system. The immunological basis of this priming is two-fold; 1) an Ag-naïve immune system has naïve B and T lymphocytes that have a much higher threshold of activation than do the Ag-primed "memory" cells of a Ag-primed immune system, such that the antigen-presenting cells (APCs) that present Ag require much less Ag to activate primed memory T cells, and 2) due to expansion of memory T cells during the Ag priming exposure, there are inherently greater numbers of such cells upon re-exposure to an injected Ag. Note that dominant APCs are dendritic cells (DCs) and macrophages that present Ag in complex with Major Histocompatibility Complex (MHC) molecules on their surface to T cell Ag receptors (**Figure 1**). It is these APCs that can influence both the "magnitude" and "type" of response to Ag; e.g., the Th1 cell response is required to clear most viral and bacterial infections, in which virus-like or bacterial-like substances (non-Ag in nature) condition APCs to express key cytokines and surface co-stimulatory molecules that, during Ag presentation, drive T cells to become the Th1 type. In fact, this APC activation is the conceptual basis of many immune enhancing substances called *adjuvants*. Some adjuvants are designed to trick the immune system into reacting to the injected vaccine Ag as if it were part of an on-going infection (i.e., infectious agents provide such natural viral or bacterial adjuvant substances). Therefore, adjuvants activate APCs for greater Ag-presentation capabilities necessary to overcome the high activation threshold of naïve T cells, in addition to shaping their development into the Th1 response to effectively clear the respective infection. Note that such T cells provide critical help to B cells that specifically bind the respective Ag to produce Ag-specific antibody (Ab) titers (**Figure 2**).

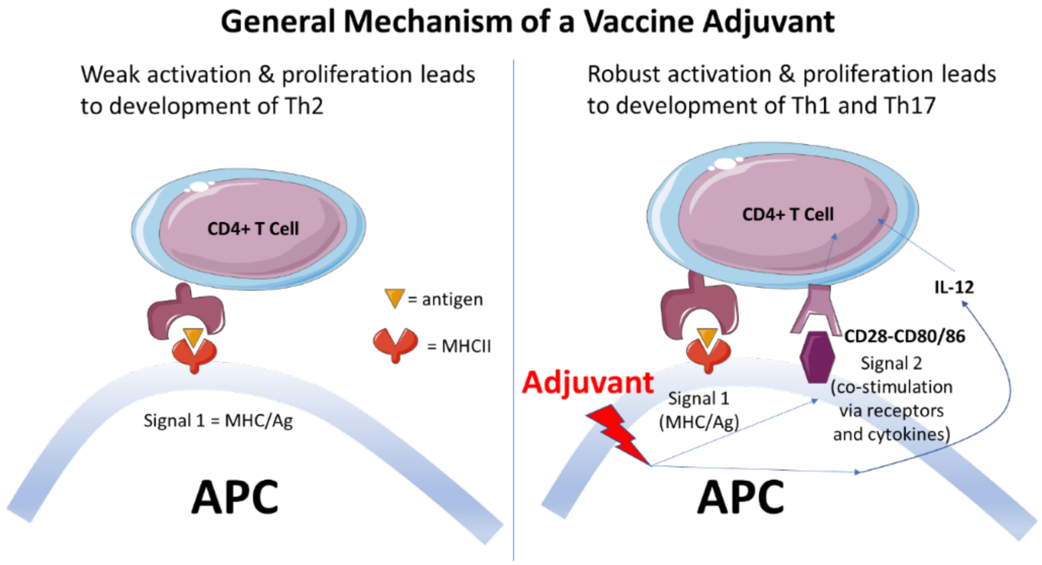

Figure 1: A general schematic of how an **adjuvant enhances T cells responses** that help B cell Ab responses. APC, Antigen-presenting cell; Th cell, T helper cell, MHC, major histocompatibility complex; Ag, antigen, IL-12, interleukin-12

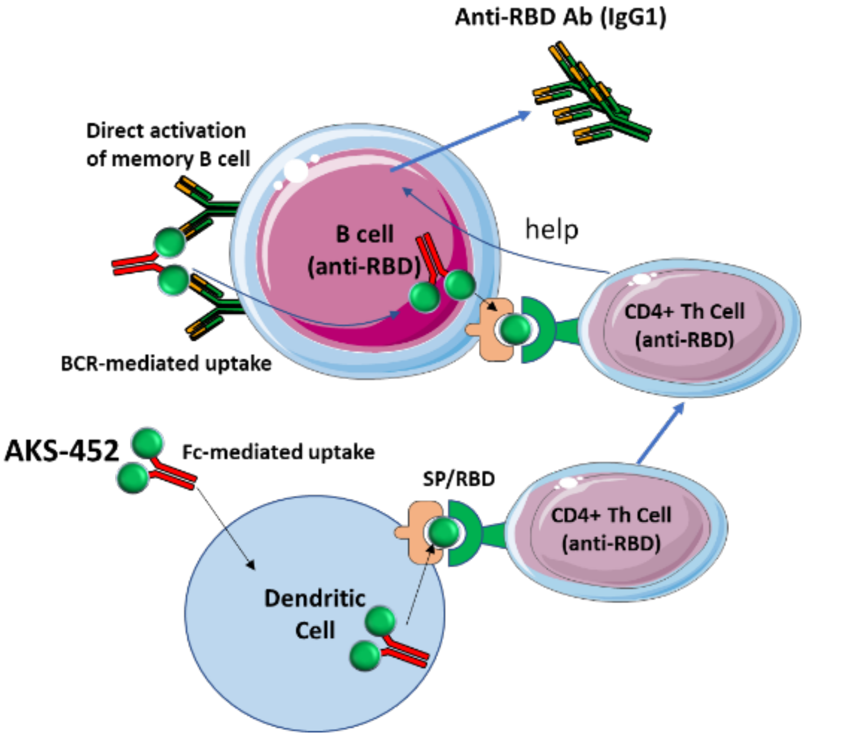

Figure 2: Mechanism of action by which AKS-452 is taken up and processed by an Ag-presenting dendritic cell that presents peptide fragments of SP/RBD to Th cells that help B cells produce anti-SP/RBS-specific Abs. AKS-452 can also directly bind existing SARS-CoV-2-specific memory B cells via their B cell receptors (BCRs) in a bivalent fashion which can crosslink BCRs enhancing proliferation, activation, and Ab production in the absence of Th cells.

Given the challenges of a recombinant SARS-Cov-2 SP subunit vaccine to induce a strong protective immune response in an immunologically naïve human population, the SP Ag must be modified and/or formulated with additional immune-enhancing features to overcome the

activation thresholds of naïve T and B cells. Akston has implemented the following such features into its COVID-19 vaccine that are major advantages over most other such vaccines in development, in which the Therapeutic Product Profile (TPP) describes details of its clinical candidate, AKS-452 (Table 1):

1. The use of the smaller focused antigenic portion of SP, the RBD
2. Recombinant fusion of RBD with human IgG1 Fc (SP/RBD-Fc)
3. Emulsification of SP/RBD-Fc in the water-in-oil adjuvant, Montanide ISA 720 for the primary vaccine in naïve individuals
4. 'Naked' SP/RBD-Fc without adjuvant (AKS-452X), as booster vaccine for previously immunized or infected individuals

The following are explanations of the above features:

1. The focused immunogenicity of the RBD Ag leads to only those Abs that bind this region on SARS-CoV-2 SP to prevent virus binding to the ACE2 target protein on host cells, thus inhibiting infection. This is in contrast to the use of a whole SP Ag vaccine that risks the generation of non-RBD-binding Abs that actually facilitate viral infection by tagging the virus for Fcγ receptor (FcγR)-mediated uptake by macrophages that act as cellular factories for viral replication (i.e., ADE). Perhaps of even greater value is that the small size of RBD provides for at least a 10-fold greater production yield relative to SP (Akston's unpublished observation).
2. However, simply injecting such a small foreign protein fragment alone as a vaccine Ag would not be expected to induce a strong enough B cell (Ab) or Th1 cell response from a naïve immune status. Therefore, Akston created the subunit vaccine, AKS-452, comprised of a bivalent analog of RBD recombinantly fused to a human IgG1 Fc moiety (**Figure 3**) that (i) facilitates the focused delivery of the RBD Ag to local APCs that internalize SP/RBD-Fc via FcγRs, and then process and present RBD fragments (**Figure 4**) [34] to $CD4^+$ Th cells that in turn promote ("help") B cell activation and anti-SARS-CoV-2 RBD IgG (i.e., Ab) production (**Figure 2**). In addition, a more direct and unique mechanism of AKS-452 is its direct binding to existing SARS-CoV-2-specific memory B cells through their Ag-specific B cell receptors (BCRs). Such binding triggers activation signals upon BCR cross-linking via the RBD bivalency feature of AKS-452 that leads to enhanced proliferation and anti-SARS-CoV-2 IgG production in the absence of $CD4^+$ Th cells (**Figure 2**). Indeed, fusion of IgG Fc with a different RBD fragment derived from the SP of the SARS virus (i.e., SARS-CoV) has been demonstrated to impart significant adjuvant activity relative to the very low immunogenicity of the SARS-RBD fragment alone [15, 17]. In fact, this human IgG Fc-fusion enhancing approach has been demonstrated with the development of a MERS vaccine containing recombinant protein of a truncated MERS SP/RBD fragment (residues 377-588) fused to human IgG Fc that increased immunogenicity via FcγR-binding on APCs, in addition to increasing the in vivo half-life and stability [18, 32]. That is, Fc enhances the systemic half-life and bioexposure of RBD to more APCs residing throughout the body due to binding the neonatal FcR (FcRn) expressed on endothelial cells that enables long serum half-lives of most monoclonal Ab (mAb) therapeutics. Another advantage of fusing RBD with Fc is the bivalency of the Ag per

Fc molecule (i.e., two RBD fragments to one Fc fragment) that improves the stoichiometric quantity of Ag delivered to APCs.

3. However, the Fc feature of Akston's vaccine Ag is likely limited for use with RBD-primed individuals who had a prior infection of SARS-CoV-2 or were previously vaccinated with a SP antigen containing the RBD. That is, FcγR binding and activation signals in APCs, while known to provide significant signals for Ag presentation, are typically not strong enough to achieve the activation threshold of naïve lymphocytes, although these signals would be expected to re-activate the low-threshold of memory T and B cells of primed individuals (**Figure 1**). In addition, the weak signaling of FcγR in naïve lymphocytes does not ensure commitment to Th1 development. To overcome these limitations for primary vaccination, Akston enhanced vaccine potency by formulating the SP/RBD-Fc Ag in the adjuvant, Montanide ISA 720, which is a water-in-oil substance containing the Th1-promoting and human-safe squalene oil. Squalene is a natural organic compound originally obtained for commercial purposes (primarily from shark liver oil), is a biochemical intermediate in plants and animals (including humans) and has been approved as an adjuvant component in several human vaccines [35]. Therefore, Montanide ISA-720 was developed for its low reactogenicity in humans and its closely related form, ISA 51, is an EU-approved adjuvant for a cancer vaccine [36-38]. Note that Montanide ISA 720 has been used in more than 200 clinical trials involving cancer, AIDS, malaria or autoimmune disease vaccines involving an accrual of more than 20,000 patients and has demonstrated an excellent clinical safety profile in addition to its strong promotion of immunogenicity and Th1 responses [37-48]. In summary, the Fc moiety on AKS-452 is designed to act as a mild adjuvant via inducing activation signaling to the APC via FcγRs and is designed to work in concert with a strong classical adjuvant, such as Montanide ISA 720, to enhance the duration of Ag exposure to APCs and perhaps direct Ag entry into lymph nodes locally and systemically where additional APCs reside. As a consequence, the Fc moiety in combination with an adjuvant is expected to create a dramatic dose-sparing potential for both the Ag and adjuvant such that the risk of reactogenicity (a safety concern) is dramatically reduced; i.e., too much adjuvant that over-activates many APCs and other innate immune cells can lead a systemic inflammatory reaction termed *reactogenicity*. Such reactogenicity is induced acutely after injection and is not mediated by T and B cells.

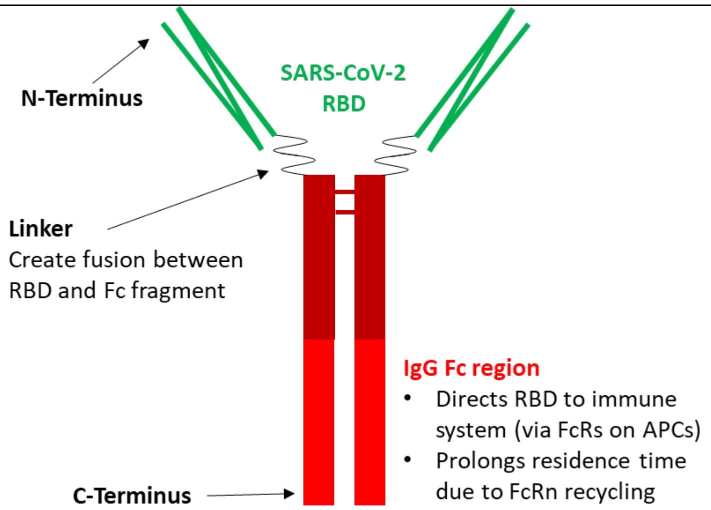

Figure 3: Schematic representation of AKS-452: 1. SARS-CoV-2 SP/RBD – enables COVID-19-specific Ag presentation to the immune system, 2. Linker – 21 amino acid sequence creating the fusion between the SP/RBD and the Fc fragment, 3. Human IgG1 Fc fragment – directs antigen presenting cells (APCs) to take up and process the SP/RBD Ag via FcγRs and enhances residence time via FcRn recycling.

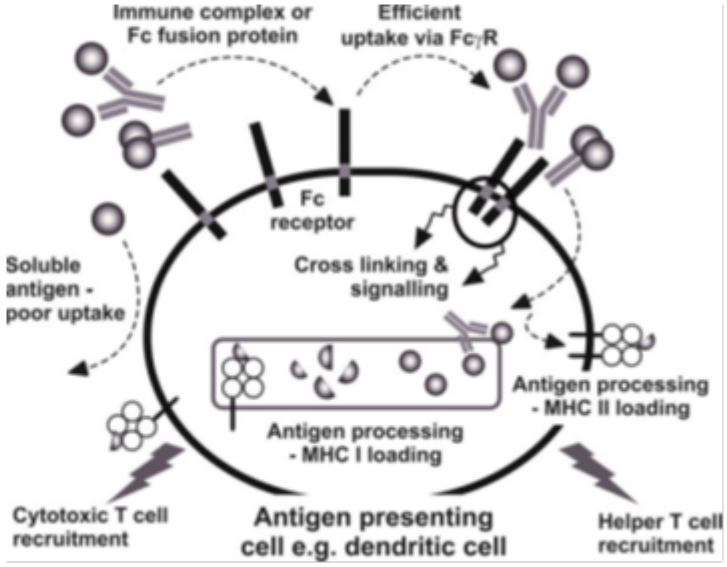

Figure 4: Fc-Ag fusion protein binds to antigen presenting cells (APCs) via the Fc$_g$Rs and is processed efficiently in the cytoplasm where Ag processing loads Major Histocompatibility Complex (MHC) class II molecules while endosomal processing loads MHC class I molecules, triggering the respective predominant cellular responses (i.e., CD4$^+$ T cell with MHCII and CD8+ T cell with MHCI) [34].

1.3 Pre-clinical experience

1.3.1 Summary of pre-clinical immunogenicity studies

Akston Biosciences' clinical development vaccine candidate, AKS-452, is a formulation of a biologically engineered SARS-CoV-2-SP/RBD-Fc fusion protein that is intended to be an injectable therapy for inducing and/or augmenting neutralizing antibody (Ab) titers against the novel SARS-CoV-2 virus in patients to address the COVID-19 pandemic. Akston's SP/RBD-Fc protein vaccine has been evaluated in mouse, NHP, and rabbit immunization studies for its capacity to safely induce high-titer neutralizing Ab responses using different dosing strategies. For primary vaccination, the SP/RBD-Fc vaccine antigen (AKS-452) has been evaluated in formulations containing a panel of adjuvants designed to amplify immunogenicity in which the water-in-oil adjuvant, Montanide™ ISA 720, performed optimally and was selected for clinical evaluation with AKS-452. AKS-452 emulsified in ISA 720 adjuvant was initially evaluated in BALB/c mice for immunogenicity and the capacity to induce production of antibodies (Abs) that bind and neutralize the Spike Protein (SP) of the SARS-CoV-2 virus. Upon binding to the Receptor Binding Domain of the SP (SP/RBD), these vaccine-induced Abs prevent the virus from attaching to the host target protein, ACE2, expressed on a variety of cell types including endothelial cells of the lung, blood vessels, and neurons. Even after a single injection of 1 to 100 µg of AKS-452 in ISA 720, substantial neutralizing Abs were induced in mice, NHPs, and rabbits that 1) bound to recombinant SP/RBD, 2) inhibited recombinant ACE2 from binding recombinant SP/RBD, and 3) prevented the SARS-CoV-2 virus from infecting live VERO-E6 cells that naturally express ACE2. Notably, the potency of the AKS-452 vaccine to induce neutralizing Abs in each of the above animal models was equal to or above the neutralization capacity of human serum obtained from convalescent COVID-19 subjects, indicating that AKS-452 in ISA 720 adjuvant should induce sufficient protection in humans. Furthermore, viral challenge studies in NHPs confirmed that AKS-452/ISA-720 protects against COVID-19 and that there is no risk of aggravation of COVID-19 disease. Additional in vitro binding studies and an in vivo viral challenge study in human ACE2 transgenic mice confirmed that AKS-452/ISA 720-induced antibodies can protect against more virulent SARS-CoV-2 strains. These assays and animal models were used to demonstrate that the optimal AKS-452 dose level in adjuvant was between 10 and 100 µg, that two doses given s.c. induced maximum immunogenic responses, and that three doses of 100 µg in ISA 720 given 14 days apart showed no toxicities or serious adverse effects in a GLP toxicology study in rabbits. As expected, mild and transient injection site reactions due to ISA 720 adjuvant were observed.

1.4. Background use of Naked Booster AKS-452 without Adjuvant Montanide (AKS-452X)

Phase I of the ACT-study has successfully been closed through the day 56 visit, and during the interim-analysis and evaluation by the DSMB, AKS-452 in all dosing groups showed a very favorable outcome in terms of safety, tolerability and immunogenicity. Based on the safety profile, seroconversion rate per dosing cohort and the statistical re-assessment, it was determined to start the subsequent randomized open-label phase II study with one cohort of 2x45 µg twice injection and 1x90 µg single-injection with each cohort consisting of 26 participants. As it becomes more apparent that the current registered vaccines may require additional boostering, especially in the face of more infectious variants (e.g. the delta mutant), the phase I study was amended with a boostering arm for all the cohorts that received a single-injection (i.e. 22.5 µg, 45 µg, and 90 µg) of the concentrated AKS-452 vaccine (AKS-452X,) which is currently underway. To maximize the benefit of the booster, the aforementioned phase I single-injection groups are receiving a single additional injection of non-adjuvanted AKS-452X at the 90 µg dose level, which was the highest and most effective dose used in the phase I adjuvanted cohorts. In the underlying Study Protocol, we propose the same booster dose for boostering subjects after vaccination with one of the four registered vaccines (i.e. Pfizer [Comirnaty], Moderna [Spikevax], Janssen [Ad26.COV2.S], AstraZeneca [Vaxzevria]). Additional justifications for implementing a 90 µg non-adjuvanted AKS-452X booster are as follows.

Pre-clinical Data

As described in the IB Section 2.2, Akston created the subunit vaccine, AKS-452, comprising a bivalent analog of RBD recombinantly fused to a human IgG1 Fc moiety to (i) facilitate the focused delivery of the RBD Ag to local APCs that internalize SP/RBD-Fc via FcγRs, and then process and present RBD fragments to CD4+ Th cells that in turn promote (“help”) B cell activation and anti-SARS-CoV-2 RBD IgG (i.e., Ab) production. In addition, a more direct and unique mechanism of AKS-452 is its direct binding to existing SARS-CoV-2-specific memory B cells through their Ag-specific B cell receptors (BCRs). Such binding triggers activation signals upon BCR cross-linking via the RBD bivalency feature of AKS-452 that leads to enhanced proliferation and anti-SARS-CoV-2 IgG production in the absence of CD4+ Th cells. However, based on existing literature and Akston’s preclinical data, the Fc feature of the vaccine on its own is likely limited to only RBD-primed individuals. That is, FcγR binding and activation signals in APCs, while known to provide significant signals for Ag presentation, are typically not strong enough to achieve the activation threshold of naïve lymphocytes, although these signals would be expected to re-activate the low threshold of memory T and B cells of primed individuals. Indeed, data presented in the IB particularly in Section 4.2.1, Figure 12, show the significant benefit in terms of antibody titers of the Montanide ISA 720-adjuvanted AKS-452 formulation in enhancing immunogenicity and Ag presentation versus non-adjuvanted AKS-452. Thus, the Montanide ISA 720-adjuvant formulation was considered for primary vaccination in naïve individuals and has indeed demonstrated the induction of high levels of anti-SP/RBD titers in the clinical setting in a dose responsive manner (see also, DSMB

report, section 5.4). Nevertheless, additional injections of non-adjuvanted AKS-452 (AKS-452X) in animal studies continue to boost titers in the non-adjuvanted AKS-452 groups as shown in IB Figures 12, 13, and 15 (mice) and 24 (rabbits). Of note, in the rabbit toxicology studies described in Section 4.2.1, the non-adjuvanted AKS-452 administered three times at 100 µg per dose was even better tolerated at the injection site than the adjuvanted formulation at the same dose. Therefore, to minimize injection site reaction, decrease the cost and supply demands of the adjuvant, and maximize the immunogenic response to AKS-452, a single Montanide ISA 720-adjuvanted AKS-452 dose followed by a non-adjuvanted booster dose of AKS-452 must be considered in the clinical setting. The data described in the IB refer to experiments in which multiple injections of either adjuvanted AKS-452 or non-adjuvanted AKS-452 are administered sequentially. To confirm the hypothesized boosting effect of non-adjuvanted AKS-452 after priming the immune system with adjuvanted AKS-452, mice from the COVID-004 experiment summarized in the IB, Table 3, and described in detail in Section 4.1.2 were followed for several weeks until their median antibody titers dropped below 500 µg/mL. The mice then received a single injection of non-adjuvanted AKS-452 at a 10 µg dose, and the resulting anti-RBD IgG (total), IgG1, IgG2a, and IgG3 antibody titers were measured at days 14, 35, and 70 following the injection. The results shown in Figs. 5 and 6 clearly demonstrate the ability of a single dose of non-adjuvanted AKS-452 to increase total IgG titers by ~3.5 fold just two weeks after the injection with levels ~4-fold higher even at 70 days after the injection. Furthermore, the Th1/Th2 response is maintained as demonstrated by a relative rise in IgG1, IgG2a, and IgG3 titers (Fig. 6).

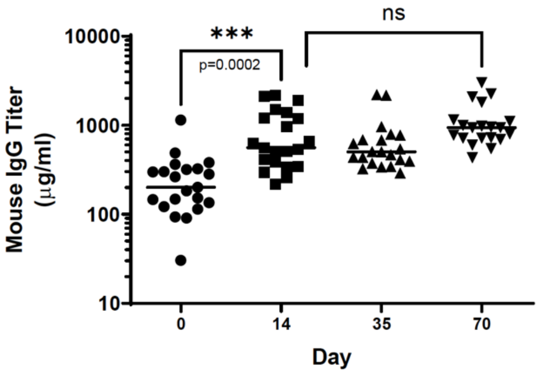

Fig. 5. Kinetics of anti-SP/RBD IgG Ab titers (Mean IgG µg/mL via ELISA reference serum standard curve) in 6-8 week-old BALB/c mice immunized with three doses of AKS-452 with Montanide™ ISA 720 adjuvant prior to the experiment and then at day 0 of this experiment with 10 µg of non-adjuvanted AKS-452.

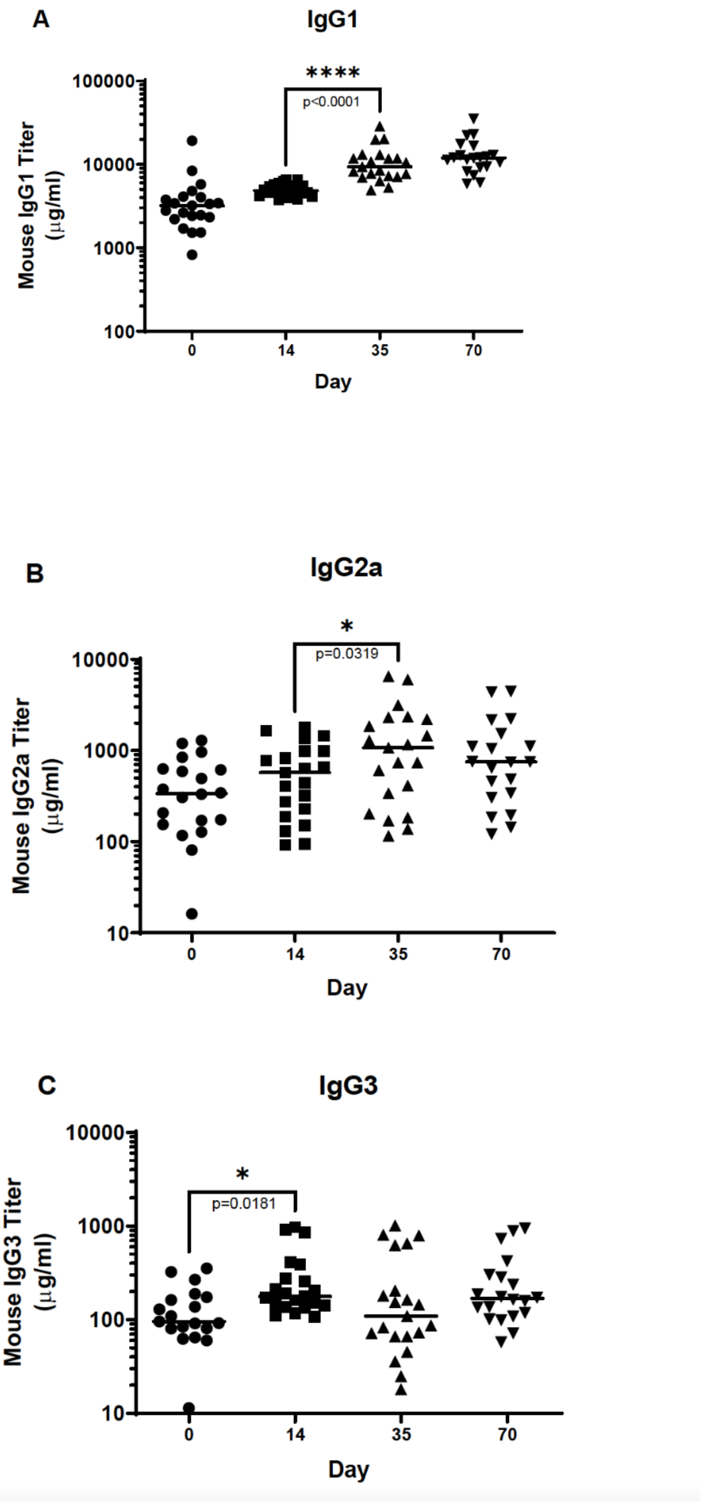

Fig. 6. Kinetics of anti-SP/RBD IgG isotype titers (Mean IgG µg/mL via ELISA reference serum standard curve) in 6-8 week-old BALB/c mice immunized with three doses of AKS-452 with Montanide™ ISA 720 adjuvant prior to the experiment and then at day 0 of this experiment with 10 µg of non-adjuvanted AKS-452. (A) IgG1 titers, (B) IgG2a titers, and (C) IgG3 titers.

1.5 Clinical Experience / DSMB Report

As AKS-452 is under investigation in a combined phase I/II clinical study, of which phase I has successfully been executed and phase II is in progress, the interim analyses and evaluation of the Data Safety Monitoring Board (DSMB) has been reported (July 8th, 2021 see also Appendix A).

'*Trial representatives informed the DSMB about the progress of the study. Furthermore, we received all necessary and relevant study material for the DSMB meeting and reviewed and discussed the safety data after the first six cohorts of patients in this trial. The trial is accomplished as described in the approved research protocol. Monitoring of the project has been carried out correctly and does not give cause for extra concern. In all dosing cohorts, mild adverse events were mainly related to the injection site and subsided after days to weeks without residual effects. ……… The DSMB concludes that the phase 1 study has not shown any safety concerns. Given this conclusion, the DSMB does not object to progressing to the phase II study with the proposed dose of 45 micrograms given twice or 90 micrograms given once subcutaneously'*.

2. OBJECTIVES

2.1 Primary Objective:

To determine the immunogenicity 4-6 weeks after subcutaneous injection of a booster dose of AKS-452X vaccine given at ≥3 months post-initial vaccination (i.e. Pfizer [Comirnaty], Moderna [Spikevax], Janssen [Ad26.COV2.S], AstraZeneca [Vaxzevria]) in human healthy volunteers.

2.2 Secondary Objectives

1. Vaccine safety and side effects –after booster vaccination. Follow-up will occur for up to 9 months post-study vaccine.
2. To evaluate the inhibitory/neutralization potency of the SP/RBD-specific IgG titers induced by AKS-452X and to estimate peak titers and duration of the response.
3. To evaluate the Th1/Th2 immune response profile.

To achieve these objectives, the following will be measured:

a. Anti-SARS-CoV-2 SP/RBD IgG titers at days 0, 28, 56, 91, 182 and 273.
b. Serum titer inhibition of recombinant ACE2-SP/RBD binding and/or neutralization of live SARS-CoV-2 virus infection of live cells (Plaque Reduction Neutralization Test, PRNT) at days 0, 28, and 182.
c. T-cell responses measured ex vivo using PBMCs to measure SP/RBD-specific T cell production of IFN-g and Th1/Th2/Th17 related cytokines via ELISpot or other Ag-specific flowcytometric-based assays on days 0, 28, and 182.

3. STUDY DESIGN

The study is designed as a single-center open-label phase II clinical study design:
Single center, open-label, safety and efficacy study on the biological activity of a SP/RBD-Fc antigen booster vaccine (AKS-452) against COVID-19.

Study population: Healthy human volunteers, 18 - 85 years, having received a registered vaccine (i.e. Pfizer [Comirnaty], Moderna [Spikevax], Janssen [Ad26.COV2.S], AstraZeneca [Vaxzevria]).

In the executed phase I study, an amendment to booster subjects with naked AKS-452 who received a single-dose injection of AKS-452 with adjuvant Montanide ISA 720 as vaccine (i.e. cohort 1: 22.5 µg, cohort 3: 45 µg, and cohort 5: 90 µg), was recently granted by the Dutch CCMO and is currently underway. The complete study will have a duration of approximately 9-12 months and will be executed by TRACER BV on behalf of Akston Biosciences in the University Medical Center Groningen (UMCG), The Netherlands, on behalf of Akston Biosciences,

Akston's clinical SP/RBD-Fc vaccine candidate is AKS-452, although Akston has experience with several protein antigen variations that have been initially evaluated in mouse immunization studies for capacity to induce high-titer neutralizing Ab responses using different dosing strategies. Such SP/RBD-Fc proteins have been evaluated as stand-alone vaccines and in formulations containing different adjuvants for screening and selection of a lead adjuvant. Key findings in mice and other species described below in the preclinical experience, Section 1.3. demonstrate the immunogenic effectiveness of the Fc moiety in addition to the strong immunogenic nature of Montanide ISA 720 that supports its use as an adjuvant.

This clinical trial strategy follows those of other organizations currently entering or having finalized Phase III trials with COVID-19 vaccines [20, 31, 49, 50] and more recently boostering with a third dose (A more detailed description of the clinical study design is illustrated in **Figure 7**).

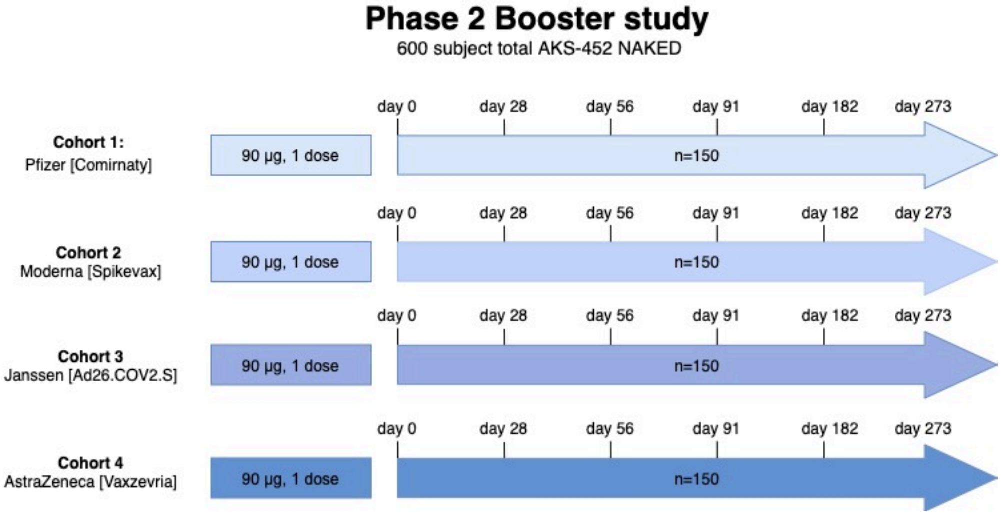

Figure 7: Study design of the Phase II ACT-BOOSTER safety and immunogenicity trial with four (4) cohort arms ((i.e. Pfizer [Comirnaty], Moderna [Spikevax], Janssen [Ad26.COV2.S], AstraZeneca [Vaxzevria]) for naked AKS-452 (90 µg subcutaneous injection) . A total of 150 subjects per cohort (taking into consideration drop-outs) will receive the booster vaccine naked, non-adjuvanted AKS- 452.

In summary, as preparatory studies prior to the booster study, in Phase I three dose-levels (22.5, 45 or 90 µg) have been administered subcutaneously (to 10 subjects per Cohort, for six Cohorts numbered 1-6). For each dose level, two dose regimens have been examined, one with a single injection of adjuvanted AKS-452 and a second cohort with a second injection of adjuvanted AKS-452 administered 28 days after the initial dose. In all cohorts, safety parameters, anti-SARS-CoV-2 spike protein RBD IgG titers and neutralizing IgG titers have been evaluated at pre-dose and also at 28, 56, 90 and 180 days after the first dose for Cohorts 1, 3 and 5. Similarly Cohorts 2, 4 and 6 have been evaluated on the same visit days, but on day 28, Cohorts 2, 4 and 6 have received a second dose of adjuvanted AKS-452.

After a safety review and immunogenicity data assessment that was conducted after the last patient in Cohort 6 completed their day 56 visit, the optimal single-dose and optimal two-dose regimens were selected for Phase 2 (i.e. 2 x 45 µg and one dosing of 90 µg).

Anti-Spike Protein Receptor Binding Domain (anti-SP-RBD) titers of phase 1 showed a dose-dependent increase at day 56, wherein for the one-dose regimen the dosing of 90 µg elicits the highest antibody response in cohort 5 with a 100% conversion rate at day 56. For the two-dose regimen, the 90 µg two-dosing leads to the highest titer-increase (cohort 6), with a 100% seroconversion rate. In addition, a comparable 100% seroconversion rate with a slightly lower titer increase at day 56 is observed for the two-dose regimen of 45 µg (Figure 8).

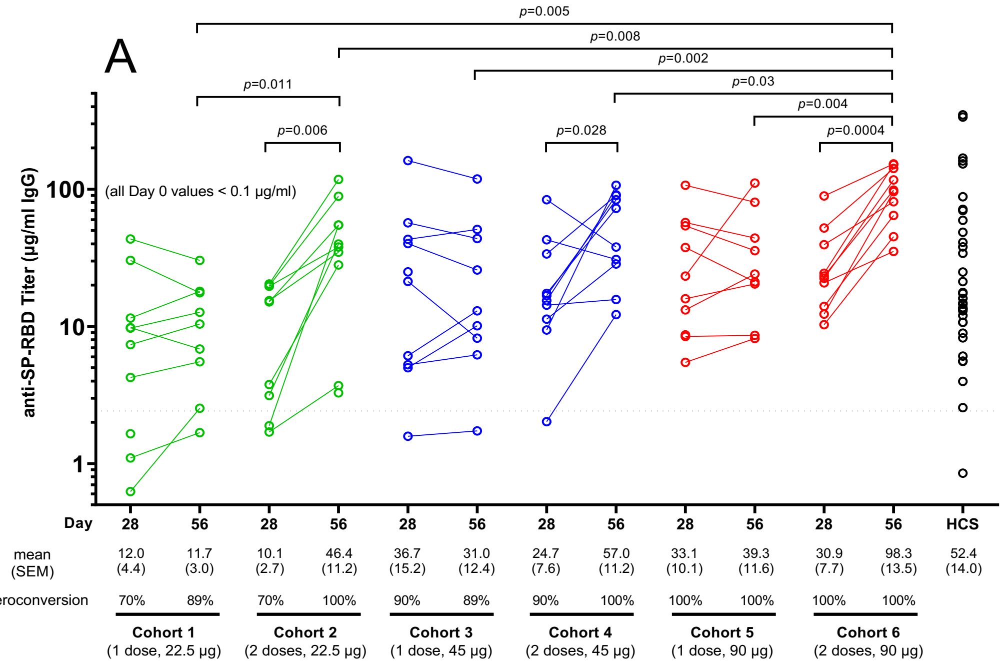

Figure 8. AKS-452 Phase 1 study immunogenicity. Serum samples were obtained at Day 0, 28, and 56 of initial vaccine dose and assessed for anti-SP/RBD IgG binding titers via ELISA (all Day 0 samples were <LLOQ; not shown). Seroconversion was defined as >2.42 µg/mL IgG (derived from validation studies with COVID-19 naïve subject samples). Human Convalescent serum (HCS) was used as a comparator for samples from vaccinated subjects.

The dose-selection for the subsequent phase II one- and two-dose regimens was based on (in addition to the primary safety data), seroconversion percentage and titer levels. Consequently, for the phase II study, the one-dose regimen of the 90 µg dosing was selected and for the two-dose regimen the 45 µg dosing. The 45 µg two-dose regimen was chosen over the 90 µg two-dose regimen to minimize the total amount of vaccine administered and the potential for AEs while ensuring an acceptable seroconversion percentage and median titer level. The DSMB as well as the CCMO confirmed and approved the dose-selection.

The Phase II AKS-452X booster study will include 150 subjects per cohort with all subjects receiving non-adjuvanted 90 µg AKS-452 vaccine with the rationale for the number of patients provided below in Section 4.4 'sample size calculation'.

Successful completion of the phase I/II trial, with a successful interim-analyses of phase I already, in addition to a sufficient scale-up of the GMP manufacturing process will enable production of a sufficiently large quantity of doses for Phase III and the future wide scale vaccine treatment of the world population with AKS-452 in ISA 720. Akston's current estimates indicate that a single 2,000 L bioreactor production train run could yield enough material for such an expected 90 µg dose of drug substance to treat approximately 70 million people receiving a single dose. A 2,000L bioreactor production train running ten times per year would therefore supply over 700 million doses of AKS-452 at 90 µg per dose. This manufacturing capacity is extremely significant and far surpasses the production throughput

and costs of the other viral-based, nucleic acid-based, and full-length recombinant SP subunit-based vaccines. The potency, manufacturability, and mechanism-of-action of the AKS-452 Fc-fusion protein formulated with / without adjuvant, resp. primary vaccination and booster vaccine, therefore, offer an opportunity to immunize billions of people globally as frequently as necessary to maintain high levels of neutralizing anti-SP/RBD Ab titers throughout the population, regardless of COVID-19 status; i.e., boosting of those with prior SARS-CoV-2 infection or of those who received a prior vaccination.

Table 1: Target product profile (TPP) of the SARS-CoV-2 vaccine, AKS-452: a recombinant human IgG Fc fusion protein with the SARS-CoV-2 spike protein receptor-binding domain (SP/RBD-Fc).

Item	Desired target
Drug structure and formulation	A subunit vaccine comprised of:<br>• a human IgG Fc moiety<br>• a linker<br>• a recombinant SP/RBD
Indications	• A primary vaccine to protect against lethal infection caused by the SARS-CoV-2 virus<br>• A boosting immunization for anti-SARS-COV-2 SP/RBD Ab positive individuals from prior infection (or immunization) to maintain immunity
Mechanism	• Induction of SP/RBD-neutralizing antibodies without causing undesired immunopotentiation such as:<br>o Th2-type immunopathology, including lung eosinophilia, and/or<br>o Antibody Dependent Enhancement (ADE) of infectivity<br>• Fc moiety focuses SP/RBD Ag to immune cells that express Fc receptors in addition to increasing Ag half-life allowing for "extended immune stimulation" to achieve single dosing
Target population	• Adults >18 years of age<br>• Suitable for adult healthcare workers<br>• At-risk, adults > 65 years old<br>• Individuals with underlying diabetes or hypertension
Route of administration	Subcutaneous
Product presentation	Multi-dose vials; 1.0 mL fill with ≥0.8 mL extractable volume of AKS-452X (concentrated)
Dosage	• 90 µg of the SP/RBD-Fc antigen.
Booster Dosage schedule	• Maximum of one booster immunization regardless of age ≥3 months after last vaccination dose<br>• Potential booster immunizations no more frequently than every 6 months
Warnings and precautions/ pregnancy and lactation	• Mild-to-moderate local injection site reactions, such as erythema, edema and pain, the character, frequency and severity of which is similar to or slightly less than licensed recombinant protein vaccines.<br>• Less than 0.01% risk of urticaria and other systemic allergic reactions.<br>• Incidence of SAEs no more than licensed comparator vaccines

Expected efficacy	Minimum of 80% efficacy in inducing protective Ab titers, potentially leading to herd immunity and a significant reduction in SARS-CoV-2 associated infections
Co-administration	All doses may be co-administered with antiviral drugs and/or other vaccines used in public health emergencies.
Storage	Refrigeration between 2-8°C. Can be out of refrigeration (at temperatures up to 25°C) for up to 2 weeks or longer, based on current stability studies.
Shelf-life	12 months at -80°C, currently one month at room temperature upon thaw or one month at 2-8°C upon thaw.
Product registration	Licensure by European regulatory agencies and FDA in the US

4. STUDY POPULATION

4.1 Population (base)

In the phase II ACT-BOOSTER study, healthy volunteers aged 18-85 years old, are recruited for this study.

The study population will be a representation of the general population in terms of characteristics as sex, ethnical background etc.

4.2 Inclusion criteria

In order to be eligible to participate in this study, a subject must meet all of the following criteria:

- Age 18-85 years (extremes included), males and females.
- Body mass index (BMI) between 19.0 and 30.0 kg/m^2, inclusive
- General good health, without significant medical illness, as determined via physical exam findings, or vital signs
- No clinically significant laboratory abnormalities as determined by the investigator
    - Note: one retest of lab tests is allowed within the screening window
- Informed Consent Form signed voluntarily before any study-related procedure is performed, indicating that the subject understands the purpose and procedures required for the study and is willing to participate in the study
- Willing to adhere to the prohibitions and restrictions specified in this protocol
- All participants have received a completed (registered) vaccine at least 3 months before inclusion in this study (i.e. Pfizer [Comirnaty], Moderna [Spikevax], Janssen [Ad26.COV2.S], AstraZeneca [Vaxzevria]).
- Negative hepatitis panel (including hepatitis B surface Ag and anti-hepatitis C virus Abs) and negative human immunodeficiency virus Ab and Ag screens at screening
- Female subjects should fulfil one of the following criteria:
    - At least 1 year post-menopausal (amenorrhea >12 months
    - Surgically sterile (bilateral oophorectomy, hysterectomy, or tubal ligation);
    - Will use adequate forms of contraceptives from screening to discharge.
- Female subjects of childbearing potential and male subjects who are sexually active with a female partner of childbearing potential must agree to the use of an effective method of birth control from screening to discharge
    - Note: medically acceptable methods of contraception that may be used by the subject and/or partner include combined oral contraceptive, contraceptive vaginal ring, contraceptive injection, intrauterine device, etonogestrel implant, double barrier, sterilization and vasectomy
- Female subject has a negative pregnancy test at screening and upon check-in at the clinical site.
    - Note: pregnancy testing will consist of a serum pregnancy test at screening and urine pregnancy tests at the dosing visit, in all women.

4.3 Exclusion criteria

A potential subject who meets any of the following criteria will be excluded from participation in this study:

- Pregnant or breast-feeding females
- Evidence of clinically significant neurologic, cardiac, pulmonary, hepatic, hematologic, rheumatologic, endocrine, autoimmune, oncologic, or renal disease
- Any laboratory test which is abnormal, and which is deemed by the Investigator(s) to be clinically significant
- Behavioral or cognitive impairment or psychiatric disease that in the opinion of the investigator affects the ability of the subject to understand and cooperate with the study protocol
- Current alcohol/illicit drug/nicotine abuse or addiction: history or evidence of current drug use or addiction (positive drug screen for amphetamines, barbiturates, benzodiazepines, cannabinoids, cocaine, or opiates) or signs of excessive use of alcohol at screening and at day 0.
- Presence of any febrile illness (T > = 38.0°C or lab confirmed viral disease (PCR)) or symptoms suggestive of a viral respiratory infection within 1 weeks prior to vaccination. Participants will be screened for SARS-Cov-2 with an EUA-approved PCR test at screening, and at day 0.
- Use of corticosteroids (excluding topical preparations for cutaneous or nasal use) or use of immunosuppressive drugs within 30 days before inoculation
- A history of anaphylaxis, history of allergic reaction to vaccine, known allergy to one of the components in AKS-452X. Mild allergies without angio-edema or treatment need can be included if deemed not to be of clinical significance (including but not limited to allergy to animals or mild seasonal hay fever)
- A history of asthma within the past 10 years, or a current diagnosis of asthma or reactive airway disease associated with exercise
- Receipt of blood or blood-derived products (including immunoglobulin) within 6 months prior to vaccination.
- Receipt of another investigational agent within 30 days or 5 times the product half-life (whichever is longest) prior to vaccination
- Deprived of freedom by an administrative or court order or in an emergency setting
- Any condition that in the opinion of the principal investigator (PI) would jeopardize the safety or rights of a person participating in the trial or would render the person unable to comply with the protocol.

4.4 Sample size calculation

The study consists of a Phase II clinical study. The following stopping-rule will be applied on a cohort-by-cohort basis: for a particular cohort, any SAE or AE ≥ 3 (according to NCI Common Terminology Criteria for Adverse Events [CTCAE]) attributable to AKS-452X.
On the basis of the phase I/II safety assessment data conducted and a minimum project enhanced immune response rate of 80% for each cohort.

Enhanced immune response rate is defined as:

i) Seroconversion based on a true positive based on the SP/RBD IgG ELISA assay positive/negative cutoff criteria using the quantitative cut-off value defined by the assay kit batch expressed in µg/mL. The positive/negative cutoff value was established as 2.42 µg/mL from the validation analysis for the current lot of assay kits, but it should be noted that for each new lot of assay kits, Akston QC performs a re-validation of the cutoff value in order to maintain clinical agreement from lot-to-lot, and/or
ii) Two times (2x) the baseline SP/RBD IgG at day 28 after boostering, as compared to the titer just before vaccination (day 0).

Based on the observed seroconversion rates in phase I/II and the statistical assessment for the phase II ACT-BOOSTER, a total study group of 150 subjects per cohort of registered vaccine (i.e. Pfizer [Comirnaty], Moderna [Spikevax], Janssen [Ad26.COV2.S], AstraZeneca [Vaxzevria]), totalling 600 subjects is regarded statistically significant to confirm the hypothesis. The statistical assessment providing the above stated numbers is based on the following argumentation (see Table 2 and Figure 9).

Fixed Scenario Elements	
Method	Normal approximation
Number of Sides	U
Null Proportion	0.7
Nominal Power	0.8
Variance Estimate	Null Variance
Alpha	0.05

Computed N Total			
Index	Proportion	Actual Power	N Total
1	0.80	0.800	119
2	0.82	0.802	81
3	0.84	0.803	58
4	0.86	0.803	43
5	0.88	0.806	33
6	0.90	0.812	26
7	0.92	0.802	20
8	0.94	0.807	16

Table 2: The POWER procedure Z test for binomial proportion

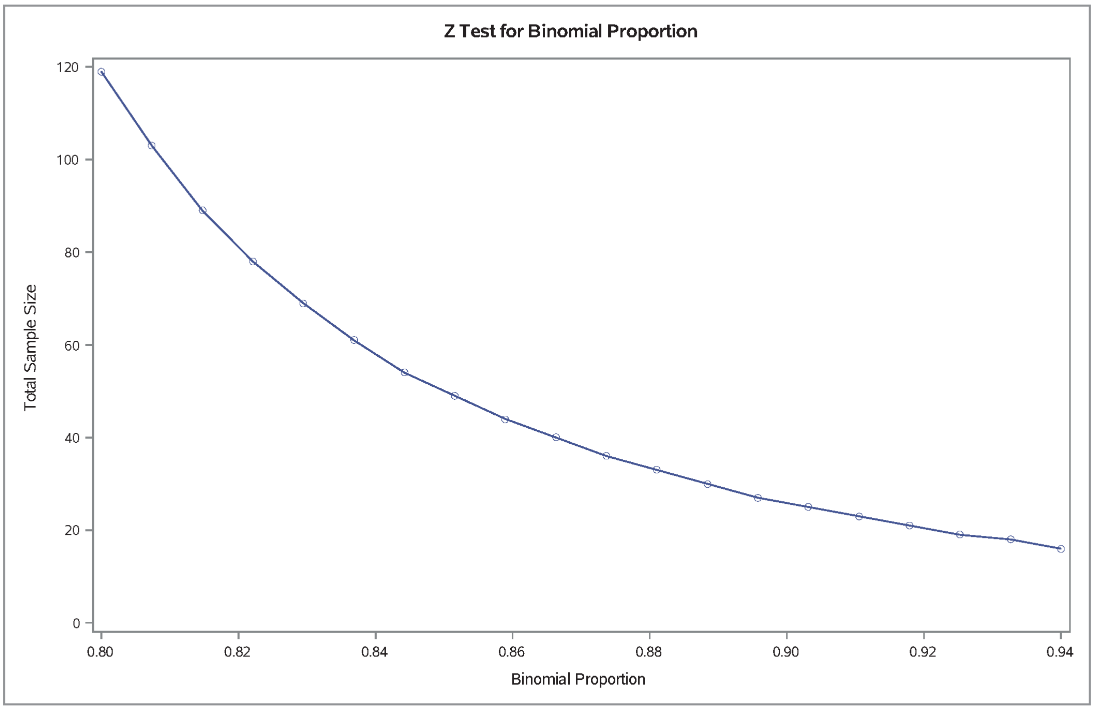

Figure 9. The POWER procedure Z test for binomial proportion

- If we consider a realistic minimum 80% conversion rate outcome in the phase II ACT-BOOSTER study, each treatment cohort will consist of 119 subjects. Considering a drop-out rate of 20%, this relates to 150 subjects per cohort (totaling 600 subjects). The minimum 80% conversion rate is considered realistic since the one single-dose injection cohort of 90 µg and the two-dose injection of 45 µg each demonstrated a 100% conversion rate in phase I/II.

Based on the above assessment of the statistical analysis, a total of 150 subjects per registered vaccine cohort will be included for the Phase II ACT-BOOSTER study, as this relates to a realistic outcome estimate based on the phase II ACT-study seroconversion rates for a single-dose injection group of 90 µg (26 subjects) and two-dose injection group of 45 µg (26 subjects), totaling 52 participants.

TREATMENT OF SUBJECTS

The subjects will receive an immunological treatment in the form of a vaccination.

4.5 Investigational product/treatment

The investigational product is a fusion-protein based vaccine.

4.6 Use of co-intervention (if applicable)

There are no-specific life-rules, diet or co-medication for the duration of the study when subjects participate.

4.7 Escape medication (if applicable)

In case of an allergic reaction, the Standard Operating Procedure 'Anaphylactic Reaction' is activated and in summary consists of the following escape medication: anti-histamine medication (e.g. clemastatine 10 mg p.o., dexamethasone (4 mg p.o.) and in case of a severe reaction epinephrine, oxygen and alarm UMCG Emergency Crash Team at the ward.

5. INVESTIGATIONAL PRODUCT

5.1 Name and description of investigational product(s)

AKS-452 is a recombinant fusion protein comprising a portion of the SARS-CoV-2 SP/RBD and an Fc fragment containing a portion of the hinge, and the full CH2, and full CH3 domains of the human IgG1 Fc fragment that are connected via a covalent peptide linker sequence (**Figure 3**). The resulting SARS-CoV-2 SP/RBD-Fc fusion protein, when made in CHO cells, is expressed as the homodimer of two identical Fc fusion protein chains that are connected via multiple disulfide bonds. The homodimer is encoded by a single nucleic acid molecule, much in the same way that a monoclonal antibody heavy chain is encoded and expressed in CHO cells. Detailed information on the production of AKS-452 and the more concentrated version AKS-452X can be found in the IMPD, section 2.1.S Drug Substance and 2.1.P Drug Product

5.2 Summary of findings from non-clinical studies

A summary of findings from non-clinical studies can be found in the Investigators Brochure (IB) and Investigational Medicinal Product Dossier (IMPD), in:

- IB AKS-452 version 3.0 (2021-09-23), section 4
- IMPD AKS-452X (concentrated) version 3.0, section 2.2

5.3 Summary of findings from clinical studies

Summary of the DSMB report (07-08-2021): Trial representatives informed the DSMB about the progress of the study. Furthermore, we received all necessary and relevant study material for the DSMB meeting and reviewed and discussed the safety data after the first six cohorts of patients in this trial. The trial is accomplished as described in the approved research protocol. Monitoring of the project has been carried out correctly and does not give cause for extra concern. In all dosing cohorts, mild adverse events were mainly related to the injection site and subsided after days to weeks without residual effects. Other AEs were likely related to the vaccine. The DSMB concludes that the phase 1 study has not shown any safety concerns. Given this conclusion, the DSMB did not object to progressing to the phase II study with the proposed dose of 45 micrograms given twice or 90 micrograms given once subcutaneously.

5.4 Summary of known and potential risks and benefits

Overall Conclusion for Phase 1 Data

The overall safety assessment of AKS-452 in the ongoing phase I/II ACT clinical study in six cohorts totaling 60 participants in phase I and 52 in phase II, showed limited side-effects over all dosing-cohorts. No SAEs attributable to the vaccine were observed. Mild AEs due to the vaccine were observed, comparable to the current registered anti-COVID-19 vaccines. Moreover, no laboratory abnormalities were observed which were attributable to the vaccine. In all dosing cohorts, a ≥70% seroconversion rate was observed, whereas the results for each of the cohorts after a single dose were comparable to values measured from confirmed COVID-19 positive convalescent serum samples. 100% seroconversion was seen in the cohorts using 2x 45 µg, 1x 90 µg and 2x 90 µg.

The repeated dose toxicity study in New Zealand White Rabbits revealed that no significant mortality or organ weight changes were observed at the main study termination. Mild effects, such as increases in fibrinogen, were most likely due to the Montanide ISA 720 adjuvant during the Dosing Phase which subsided during the recovery phase. The expected macroscopic and microscopic transient redness of the injection site were also likely due to adjuvant. These findings from the animal toxicity studies on the adjuvant are in line with clinical findings of adverse events in clinical trials. A potential risk is an allergic reaction after the injection of AKS-452X. In this trial, to guard the subjects' safety during and after AKS-452X injection, they will be closely observed until 15 minutes post injection by qualified nurses, who will be ready to promptly intervene.

5.5 Description and justification of route of administration and dosage

In the underlying RP of the phase II AKS-452X ACT-BOOSTER trial, one booster dosage will be investigated in terms of enhanced immune response and secondary safety. Based on the preclinical trials in rabbits, non-human primates and clinical data we anticipate that one dose of 90 µg will generate sufficient seroconversion and/or immune enhancement to protect against SARS-CoV-2 infection after having received a registered vaccine ≥3 months ago. The route of administration for the human clinical setting is subcutaneous injection based on (pre-)clinical studies. The trial consists of four study arms of the four registered vaccines (**Figure 7**), of 150 subjects per cohort of registered vaccine (i.e. Pfizer [Comirnaty], Moderna [Spikevax], Janssen [Ad26.COV2.S], AstraZeneca [Vaxzevria]), totalling 600 subjects

5.6 Dosages, dosage modifications and method of administration

Naked AKS-452X (5x concentrated without Montanide ISA 720 adjuvant) will be mixed with sterile saline at a 1:4 ratio (v/v), and doses of 90 µg (with a total volume of 150 µL of the diluted AKS-452X solution) will be injected subcutaneously. For more details see 6.5.

5.7 Preparation and labelling of Investigational Medicinal Product

AKS-452X has been produced according to GMP standards. The final product has been verified by an identity method based on SEC-HPLC. The product has been filled in vials under GMP conditions to a final concentration of 3000 µg/mL in phosphate buffered saline (pH 7.2). The vials are stored frozen at -80°C, but are thawed on day of use. A copy of the primary and secondary label according to GMP standards is provided in the IMPD.

5.8 Drug accountability

The investigational agents are stored at the (co-)investigators pharmacy (Department of Clinical Pharmacy, University Medical Center Groningen, The Netherlands). If investigational agents would need to be shipped (for example after creating aliquots of the study compound by an external subcontractor), they will be shipped to the Principal Investigator's authorized designee, who will check and record the amount and condition of the agents received. All shipments of investigational agent will include an enclosed packing slip; the slip must be signed by the designee and returned to the shipper to verify receipt of investigational agent. At the end of the study or as directed, all used and unused supplies, including partially used or empty containers,

will be disposed of or transferred in accordance with local written procedures, if applicable. Any disposal or transfer of investigational agent shall be noted on the investigational drug disposition log and signed-off by a second person.

6. NON-INVESTIGATIONAL PRODUCT

6.1 Name and description of non-investigational product(s)

Not applicable

6.2 Summary of findings from non-clinical studies

Not applicable

6.3 Summary of findings from clinical studies

Not applicable

6.4 Summary of known and potential risks and benefits

Not applicable

6.5 Description and justification of route of administration and dosage

Not applicable

6.6 Dosages, dosage modifications and method of administration

Not applicable

6.7 Preparation and labelling of Non-Investigational Medicinal Product

Not applicable

6.8 Drug accountability

Not applicable

7. METHODS

7.1 Study parameters/endpoints

7.1.1 Main study parameter/endpoint

Main study parameters/endpoints: Primary endpoint: The percentage of patients that i) achieve an SP/RBD-specific IgG antibody titer level of ≥ 2.42 µg/mL at the day 28 time-point post-intervention (i.e. booster vaccine) if the base-line value prior to receiving the booster vaccine was < 2.42 µg/mL or ii) where the SP/RBD-specific IgG antibody titer is at least 2x the base-line value prior to receiving the booster vaccine if the base-line value prior to receiving the booster vaccine was ≥ 2.42 µg/mL. The percentage of patients in each of the four cohorts that achieve the primary endpoint threshold at 28 days post-intervention will be calculated (n (%)).

7.1.2 Secondary study parameters/endpoints

Safety evaluation in the four cohorts for local and systemic adverse events after injection every pre-defined scheduled follow-up (post intervention. Patients will continue to be followed passively for additional safety events out to 9 months post-intervention.

To achieve these objectives, the following will be measured:

- o Anti-SARS-CoV-2 SP RBD IgG titers at days 0, 28, 56, 91, 182 and 273 post-boostering.
- o Serum titer inhibition of recombinant ACE2-SP/RBD binding and/or neutralization of live SARS-CoV-2 virus infection of live cells (Plaque Reduction Neutralization Test, PRNT) at days 0, 28, and 182
- o T-cell responses measured ex vivo using PBMCs to measure SP/RBD-specific T cell production of IFN-γ and Th1/Th2/Th17 related cytokines via ELISpot or other Ag-specific flowcytometric-based assays on days 0, 28, and 182.

7.1.3 Other study parameters (if applicable)

Not applicable.

7.2 Randomisation, blinding and treatment allocation

The study is a single-centre, open-label Phase II clinical AKS-452X booster vaccine-evaluation study. No randomisation is applicable, nor blinding. All subjects included will receive the active vaccine AKS-452 without adjuvant. The Phase II study will comprise 150 subjects per cohort (all subjects will receive the active vaccine).

7.3 Study procedures phase I (including booster with naked AKS-452)

- AKS-452X will be mixed with saline at the pharmacy and doses of 90 µg (with a total volume of 150 µL) will be injected subcutaneously.
- In the Table below, the Proposed clinical Schedule for phase II is provided.

	Screening Visit	Visit 1 (first dose)	Visit 2	Visit 3	Visit 4	Visit 5	Visit 6
	Day -28 to -2	Day 0	Day 28±2	Day 56±2	Day 91±5	Day 182±7	Day 273 ±7
**Screening/Administrative/Other Assessments**							
Informed consent	X						
Demography	X						
Eligibility criteria	X	X					
Medical and medication history	X						
Serology[a]	X						
Drug screen[b]	X	X					
COVID-19 PCR	X	X					
**Safety Assessments**							
Physical exam	X						
Vital signs	X	X					
Blood pregnancy test[c]	X						
Urine pregnancy test[c]		X					
Blood for safety[a]	X	X					
Vaccination Questionnaire			X[f]				
Concomittant therapy, AEs/SAEs[e]		X	X	X	X	X	X
**Study Agent Administration/Pharmacokinetics and Immunology Assessments**							
Fc-conjugation vaccine administration[d]		X					
Peripheral blood for humoral responses		X[g]	X	X	X	X	X
Peripheral blood for PBMCs (cellular responses)		X[g]	X			X	

a) See Laboratory assessments for list of tests.
b) Using instant urine drug test kit
c) In all women
d) After vaccination, subjects are to be kept under medical supervision for 15 min.
e) Subjects who terminate the study early will be asked to return to the clinical site within 14 days after discontinuation for safety assessments
f) To be taken on paper. Subjects will be asked to log all (non-important) AE's between visits
g) Sample to be taken pre-vaccine administration

7.4 Withdrawal of individual subjects

Subjects can leave the study at any time for any reason if they wish to do so without any consequences. The investigator can decide to withdraw a subject from the study for urgent medical reasons. In case of drop-outs of this study for any reason, these subjects are kindly asked to complete the follow up period of 273 days for reasons of safety and efficacy. It will also provide the investigators with crucial information about protection against viral infection after multiple vaccine administrations and the AKS-452X booster. The request to complete the follow up will only apply when the subjects have received the AKS-452X booster vaccine.

7.4.1 Specific criteria for withdrawal (if applicable):

Not applicable.

7.5 Replacement of individual subjects after withdrawal

Subjects who are withdrawn may be replaced until the intended number of subjects determined by the statistical assessment (i.e. a minimum of 119 subjects per cohort) is reached by this study. The SARS-CoV-2 IgG titer of day 28 can be used for the interim analysis of day 56.

7.6 Follow-up of subjects withdrawn from treatment

After administration of the active vaccine, (serious) adverse events that occur in subjects that are withdrawn from the study procedure and preferably antibody titers, will still be recorded if possible.

7.7 . Premature termination of the study

Termination based on safety aspects: A multidisciplinary team with study investigators ([co]PI’s and dedicated research physicians) together with the sponsor will discuss safety aspects during the study procedures.

The CTCAE stopping rules for first in human studies will be leading to grade a possible SAE and how to proceed if an SAE occurs.

The termination rules are as follows: When an SAE and/or AE ≥ grade 3 NCI CTCAE (attributable to AKS-452X) occurs after the s.c. injection: terminate the study for all cohorts (e.g. if an SAE occurs in cohort 1, 2, 3, or 4, terminate the entire study).

Termination based on other aspects: The study will be suspended based on urgent medical or ethical considerations as decided by the principal investigators (additional to section 8.7). In case of termination of the study, the sponsor, the institution, regulatory authorities (CCMO will be informed. Moreover, sponsor’s medical monitor (or designee) may stop or suspend the trial due to safety concerns from the sponsor. Furthermore, sponsor may stop the trial due to other concerns.

Mandatory: A cohort must demonstrate a minimum of 80% seroconversion (in case a subject dropouts before day 28, he/she will be replaced with another subject. A cohort must include a

minimum of 119 subjects for final analysis), that have an enhanced immune response as defined as following:

Enhanced immune response rate is defined as:

i) Seroconversion based on a true positive based on the SP/RBD IgG ELISA assay positive/negative cutoff criteria using the quantitative cut-off value defined by the assay kit batch expressed in µg/mL. The positive/negative cutoff value was established as 2.42 µg/mL from the validation analysis for the current lot of assay kits, but it should be noted that for each new lot of assay kits, Akston QC performs a re-validation of the cutoff value in order to maintain clinical agreement from lot-to-lot, and/or

ii) Two times (2x) the baseline SP/RBD IgG at day 56 after a boostering, as compared to the titer at the time of screening (day 0).

8. SAFETY REPORTING

8.1 Temporary halt for reasons of subject safety

In accordance with section 10, subsection 4, of the WMO, the sponsor will suspend the study if there are sufficient grounds that continuation of the study will jeopardise the subject's health or safety. The sponsor will notify the CCMO without undue delay of a temporary halt, including the reason for such an action. The study will be suspended pending a further positive decisions by the CCMO. The investigator will take care that all subjects are kept informed.

8.2 AEs, SAEs and SUSARs

8.2.1 Adverse events (AEs)

Adverse events are defined as any undesirable experience occurring to a subject during the study, whether or not considered related to the investigational product / trial procedure / the experimental intervention. All adverse events reported spontaneously by the subject or observed by the investigator or his staff will be recorded. Subjects will be monitored throughout the study for AEs, from the time of first dose on Day 0 up to the assessments of Day 273. Untoward events that occur prior to the booster dose of the study vaccine will not be recorded as an AE but should be recorded as medical history. Adverse events that are identified at the last assessment visit (or the early termination visit) as specified in the protocol must be recorded on the AE CRF with the status of the AE noted, and the AE must be followed until resolution. AEs that are resolved at the final study visit should be recorded with a stop date. All AEs should be followed to resolution whenever possible. Action taken will be categorized as none, study drug discontinued, dose modified, required concomitant medication, required procedure, or other. Event outcome at resolution or time of last evaluation will be recorded as event resolved, resolved with sequelae, ongoing, or death.

Severity. For evaluating severity and relatedness to AKS-452X, CTCAE will be used (CTCAE V5.0). Adverse events will be graded by a numerical score according to the defined NCI Common Terminology Criteria for Adverse Events (NCI CTCAE) and version number specified in the protocol. Adverse events not specifically defined in the NCI CTCAE will be scored on the Adverse Event log according to the general guidelines provided by the NCI CTCAE and as outlined below:

- Grade 1: Mild
- Grade 2: Moderate
- Grade 3: Severe or medically significant but not immediately life threatening
- Grade 4: Life threatening consequences
- Grade 5: Death related to the adverse event

Relationship to treatment

The relationship of the event to the study drug should be determined by the Investigator according to the following criteria:

- Not related: The event is most likely produced by other factors such as the subject's clinical condition, intercurrent illness, or concomitant drugs, and does not follow a

known response pattern to the study drug, or the temporal relationship of the event to study drug administration makes a causal relationship unlikely.

- Possibly related: The event follows a reasonable temporal sequence from the time of drug administration, and/or follows a known response pattern to the study drug, but is more likely produced by other factors such as the subject's clinical condition, intercurrent illness, or concomitant drugs.
- Probably related: The event follows a reasonable temporal sequence from the time of drug administration, and/or follows a known response pattern to the study drug but could be explained by other factors such as the subject's clinical condition, intercurrent illness, or concomitant drugs.
- Definitely related: The event follows a reasonable temporal sequence from the time of drug administration, and/or follows a known response pattern to the study drug and cannot be reasonably explained by other factors such as the subject's clinical condition, intercurrent illness, or concomitant drugs.

8.2.2 Serious adverse events (SAEs)

A serious adverse event is any untoward medical occurrence or effect that

- results in death;
- is life threatening (at the time of the event);
- requires hospitalisation or prolongation of existing in subjects' hospitalization;
- results in persistent or significant disability or incapacity;
- is a congenital anomaly or birth defect; or
- any other important medical event that did not result in any of the outcomes listed above due to medical or surgical intervention but could have been based upon appropriate judgement by the investigator.
- An elective hospital admission will not be considered as a serious adverse event.

The sponsor will report the SAEs through the web portal *ToetsingOnline* to the accredited CCMO that approved the protocol, within 7 days of first knowledge for SAEs that result in death or are life threatening followed by a period of maximum of 8 days to complete the initial preliminary report. All other SAEs will be reported within a period of maximum 15 days after the sponsor has first knowledge of the serious adverse events.

8.2.3 Suspected unexpected serious adverse reactions (SUSARs)

Adverse reactions are all untoward and unintended responses to an investigational product related to any dose administered.

Unexpected adverse reactions are SUSARs if the following three conditions are met:

1. the event must be serious (see chapter 9.2.2);
2. there must be a certain degree of probability that the event is a harmful and an undesirable reaction to the medicinal product under investigation, regardless of the administered dose;

3. the adverse reaction must be unexpected, that is to say, the nature and severity of the adverse reaction are not in agreement with the product information as recorded in:
   - Summary of Product Characteristics (SPC) for an authorised medicinal product;
   - Investigator's Brochure for an unauthorised medicinal product.

The sponsor will report expedited the following SUSARs through the web portal *ToetsingOnline* to the CCMO:

- SUSARs that have arisen in the clinical trial that was assessed by the CCMO;
- SUSARs that have arisen in other clinical trials of the same sponsor and with the same medicinal product, and that could have consequences for the safety of the subjects involved in the clinical trial that was assessed by the CCMO.

The remaining SUSARs are recorded in an overview list (line-listing) that will be submitted once every half year to the CCMO. This line-listing provides an overview of all SUSARs from the study medicine, accompanied by a brief report highlighting the main points of concern.

The expedited reporting of SUSARs through the web portal Eudravigilance or ToetsingOnline is sufficient as notification to the competent authority.

The sponsor will report expedited all SUSARs to the competent authorities in other Member States, according to the requirements of the Member States.

The expedited reporting will occur not later than 15 days after the sponsor has first knowledge of the adverse reactions. For fatal or life-threatening cases the term will be maximal 7 days for a preliminary report with another 8 days for completion of the report.

8.3 Annual safety report

In addition to the expedited reporting of SUSARs, the sponsor will submit, once a year throughout the clinical trial, a safety report to the CCMO, and competent authorities of the concerned Member States.

This safety report consists of:

- a list of all suspected (unexpected or expected) serious adverse reactions, along with an aggregated summary table of all reported serious adverse reactions, ordered by organ system, per study;
- a report concerning the safety of the subjects, consisting of a complete safety analysis and an evaluation of the balance between the efficacy and the harmfulness of the medicine under investigation.

8.4 Follow-up of adverse events

All AEs will be followed until they have abated, or until a stable situation has been reached. Depending on the event, follow up may require additional tests or medical procedures as indicated, and/or referral to the general physician or a medical specialist.
SAEs need to be reported till end of study within the Netherlands, as defined in the protocol

8.5 Data Safety Monitoring Board (DSMB) / Safety Committee

No DSMB will be installed for the phase II booster study.

9. STATISTICAL ANALYSIS

9.1 Analysis Populations

Safety Population: The Safety Population will consist of all subjects who received a booster dose of AKS-452X of 90 µg s.c..

9.2 Descriptive statistics

Patient demographic characteristics (such as age, sex, race, ethnicity, BMI, medical history and morbidity) will be displayed as: (geometric) means with standard deviations, medians with range and frequencies.

Continuous variables will be inspected for normal distribution by histograms and if non-normally distributed, attempts will be made to transform the data to obtain a normal distribution.

9.3 Primary study parameter(s)

The primary aim of this booster vaccination study is to investigate if a subcutaneous (s.c.) booster dose of 90 µg of the naked Akston AKS-452X vaccine at ≥ 3 months post initial vaccination, with any of the four registered vaccines, will boost the antibody titer and immune response in human healthy volunteers 4weeks after s.c. injection.

Safety: Adverse events are defined as any undesirable experience occurring to a subject during the study, whether or not considered related to the investigational product / trial procedure/ the experimental intervention. All adverse events reported spontaneously by the subject or observed by the investigator or his staff will be recorded.

Evaluation of Safety and Tolerability:
Safety and tolerability will be assessed through AEs and clinical laboratory, and any other parameter that is relevant for safety assessment.
Adverse Events: a listing of all individual AEs will be provided. Summary tables of TEAEs will be presented by system organ class based on the MedDRA terminology list (preferred terms): containing the number of TEAEs (frequency of occurrence, number of subjects experiencing the event) by treatment and containing the number of drug-related TEAEs (frequency of occurrence, number of subjects experiencing the event) per treatment. Additional tables of total counts by treatment and relationship and by treatment and severity will be given.

Laboratory data: clinical laboratory data will be listed accompanied by an indication if the parameter is outside the reference range. A summary of all data outside the reference range of the clinical laboratory will be provided. Clinical laboratory data will be presented descriptively, where applicable.

9.4 Secondary study parameter(s)

- To evaluate the inhibitory/neutralization potency of the SP/RBD-specific IgG titers induced AKS-452X and to estimate peak titers and duration of the response.
- To evaluate the Th1/Th2 immune response profile.

To achieve these objectives, the following will be measured:

- o Anti-SARS-CoV-2 SP RBD IgG titers at days 0, 28, 56, 91, 182 and 273.
- o Serum titer inhibition of recombinant ACE2-SP/RBD binding and/or neutralization of live SARS-CoV-2 virus infection of live cells (Plaque Reduction Neutralization Test, PRNT) at days 0, 28, and 182
- o T-cell responses measured ex vivo using PBMCs to measure SP/RBD-specific T cell production of IFN-γ and Th1/Th2/Th17 related cytokines via ELISpot or other Ag-specific flowcytometric-based assays on days 0, 28, and 182.

9.5 Other study parameters

Not applicable

9.6 Interim analysis (if applicable)

n.a.

Overall Conclusion for Phase I/II Data

The overall safety assessment of AKS-452X in the current ongoing phase I/II ACT clinical study in six cohorts totaling 60 participants for phase I and 52 for phase II, showed limited side-effects over all dosing-cohorts. No SAEs attributable to the vaccine were observed. Mild AEs due to the vaccine were observed, comparable to or slightly less than the current registered anti-COVID-19 vaccines. Moreover, no laboratory abnormalities were observed which were attributable to the vaccine. In all dosing cohorts, a ≥70% seroconversion rate was observed, whereas the results for each of the cohorts after a single dose were comparable to values measured from confirmed COVID-19 positive convalescent serum samples.

10. ETHICAL CONSIDERATIONS

10.1 Regulation statement

The study will be conducted according to the principles of the Declaration of Helsinki (Fortaleza, Brazil, 2013 amendment) and in accordance with the medical Research Involving Human Subjects Act (WMO) and other guidelines, regulations and Acts. The protocol has been written and the study will be conducted according to the ICH Harmonized Tripartite Guideline for Good Clinical Practice (ICH E6). The protocol will be approved by the National Ethics Committee (CCMO) as part of a fast trajectory process for COVID-19 studies.

10.2 Recruitment and consent

Potential eligible subjects are recruited by posting announcements on social media and through local or regional newspapers. A potential subject can express interest in the study in an online application form, after which the subject will receive the participant information folder by email.

In the emailed information (after initial application) the subjects will be informed about the aims of the study, the possible adverse events, the procedures and possible hazards to which they will be exposed before enrolment into the study. They will be informed as to the maintenance of confidentiality of their patient data.

Each patient will be given the opportunity to ask questions and will be informed about the right to withdraw from the study at any time without prejudice. See the patient information sheet and patient informed consent statement. When subjects affirm their intent to participate, a pre-treatment consultation is planned and subjects will be asked to bring the signed informed consent form, which will be received by the research physician.

For the phase II ACT-BOOSTER study an online survey (pre-screening instrument) will be used to check the eligibility of potential subjects. This survey focusses on checking in- and exclusion criteria. When a subject is deemed eligible for participating in the study, he/she will be planned for a screening consultation visit. Each patient will be given the opportunity to ask questions and will be informed about the right to withdraw from the study at any time without prejudice.

Informed consent

Documented informed consent must be obtained for all subjects included in the study before they are registered in the study. Subjects must be given adequate opportunity to read the information and enquire about details of the study before consent is given. The informed consent procedure conforms to the ICH guidelines on Good Clinical Practice. This implies that the written informed consent form will be signed and personally dated by the patient or by the patient's legally acceptable representative. The informed consent statement will be signed and dated by the research physician afterwards and the patient will receive a copy. The general physician of each patient will be informed about the enrolment of the patient to the study.

10.3 Objection by minors or incapacitated subjects (if applicable)

Not applicable

10.4 Benefits and risks assessment, group relatedness

For the participating subjects, there is formally no objective diagnostic, preventive or treatment benefit related to the study, as this is a phase II booster vaccine clinical study. Since all subjects receive the active vaccine, there is the anticipated effect of protection against a COVID-19 infection. Participation may possibly produce useful scientific data for the future and the impact for fighting the COVID-19 pandemic. The risks related to AKS-452X administration are described in section 6.1, the IB and IMPD.

The risk associated with exposure to a novel vaccine for a phase II clinical booster study are pain/swelling/redness/bleeding/infection/granuloma formation at SC injection site, hematoma due to the venepuncture for blood sampling, mild fever, chills, feeling tired, headache, muscle and joint aches, syncope, and an allergic reaction related to AKS-452X, which are all well within the tolerable range for a novel vaccine like AKS-452X as based on the animal toxicology data and the phase I/II ACT-study clinical data. The benefit, in case of a safe and sufficient enhanced immunogenicity provoking vaccine, is for protecting health care workers, future vulnerable patients and frail elderly, and patients undergoing large surgical procedures for instance oncology, transplantation etc. Moreover, providing protection in co-morbid citizens (i.e., diabetes, overweight, cardiovascular disease etc) and ultimately, creating another leverage to returning societies back to their previous health care system capacities and economic growth world-wide.

10.5 Compensation for injury

As this study is a sponsor-initiated study, the investigators employer has liability insurance which is in accordance with article 7 of the WMO. This insurance provides coverage for damage to research subjects through injury or death caused by the study. The insurance applies to the damage that becomes apparent during the study or within 4 years after the end of the study.

10.6 Incentives (if applicable)

For each day of patient related study procedures, the subjects will receive compensation for travelling expenses (€ 0.19/km) and a ticket for free parking. Furthermore, a participation fee of € 500 euros will be given to every participant taking part in this study.

11. ADMINISTRATIVE ASPECTS, MONITORING AND PUBLICATION

11.1 Handling and storage of data and documents

Case Record Forms (eCRF) - REDCap will be used for clinical data management. eCRFs are designed within REDCap to collect and store study data. Most data will be entered directly in REDCap. Data like medical history, ECG, AE's and instant urine drug tests will also be stored in the source documents and/or electronic patient dossier (EPD). The investigators are responsible for the legibility, completeness and correctness of the CRF. The Principal Investigator (PI) can track subjects and lock patient data when all study duties are fulfilled. Errors, changes and/or additions to the CRF are tracked by the program.
The PI and main investigators will have all access to data, all other investigators who help during measurements will only be allowed to add data to the CRF.

Data storage - Data of subjects will be handled confidentially and a coded identification number (study protocol name 'ACT') followed by the patient number of inclusion (for example '01') will be used to link the data to the specific patient. A decoding file of the data will be stored by the PI and is only accessible by the PI. The handling of the personal data complies with the EU law: General Data Protection Regulation. These data will be stored at the specific site for at least 25 years. Coded study data will be made available to relevant partners within the project.

Data sharing - Medically relevant information will be coded and shared with Akston Biosciences under the terms of a clinical trial agreement (CTA) between the Sponsor (Akston Biosciences) and the UMCG. Akston Biosciences will be the owner of the data under the agreement. Akston Biosciences will not receive the key that safeguards the data, as this will remain in the possession of the coordinating investigator of the CRO. In this way no privacy sensitive information will be shared with Akston Biosciences. This is also stated in the patient information provided for this study.

11.2 Monitoring and Quality Assurance

On-site monitoring will take place conform the NFU (Nederlandse Federatie van Universitair Medische Centra)-guideline "Kwaliteitsborging van mensgebonden onderzoek 2020" by the independent and qualified monitor. For this study, the risk classification is considered "negligible", which implies intensive independent monitoring of at least 3 visits per year, dependent on the patient inclusion speed. This study will be monitored by independent certified monitors, employed at the UMCG (i.e. IMO Research Office, UMCG). The monitors will perform source data verification on the research data by comparing the data entered into the CRF with the available source documentation and other available documents. Source documents are defined as the patient's hospital medical records, clinician notes, laboratory print outs, digital and hard copies of imaging, memos, electronic data etc.

11.3 Amendments

Amendments are changes made to the research after a favorable opinion by the CCMO has been given. All amendments will be notified to the CCMO that gave a favorable opinion. A 'substantial amendment' is defined as an amendment to the terms of the CCMO application, or

to the protocol or any other supporting documentation, that is likely to affect to a significant degree:

- the safety or physical or mental integrity of the subjects of the trial;
- the scientific value of the trial;
- the conduct or management of the trial; or
- the quality or safety of any intervention used in the trial.

All substantial amendments will be notified to the CCMO and to the competent authority. Non-substantial amendments will not be notified to the CCMO as competent authority but will be recorded and filed by the sponsor.

11.4 Annual progress report

The sponsor/investigator will submit a summary of the progress of the trial to the CCMO once a year. Information will be provided on the date of inclusion of the first subject, numbers of subjects included and numbers of subjects that have completed the trial, serious adverse events/ serious adverse reactions, other problems, and amendments.

11.5 Temporary halt and (prematurely) end of study report

The sponsor will notify the CCMO as the competent authority of the end of the study within a period of 90 days. The end of the study is defined as the last patient's last visit. The sponsor will notify the CCMO immediately of a temporary halt of the study, including the reason of such an action. In case the study is ended prematurely, the sponsor will notify the CCMO and the competent authority within 15 days, including the reasons for the premature termination. Within one year after the end of the study, the investigator/sponsor will submit a final study report with the results of the study, including any publications/abstracts of the study, to the CCMO as the Competent Authority

11.6 Public disclosure and publication policy

The study will be registered in a public trial registry (www.clinicaltrials.gov). The sponsor of the study is Akston Biosciences Corporation, where TRACER BV acts as its legal representative in the Netherlands. All research contracts as required by the CRO TRACER BV are signed and stored. The study results will be published in academic journals.

12. STRUCTURED RISK ANALYSIS

Nature and extent of the burden and risks associated with participation, benefit and group relatedness:

The burden of participating in the study will be the number of site visits and thus travelling for subjects in phase II, blood samples for measurement of immunogenicity, physical examination prior to inclusion / exclusion, physical discomfort related to the subcutaneous injection of naked AKS-452X. The risk associated with exposure to the vaccine for a phase II clinical study are pain/swelling/redness/bleeding/infection/granuloma formation at SC injection site, mild fever, chills, feeling tired, headache, muscle and joint aches, syncope, allergic reaction.

12.1 Potential issues of concern

Level of knowledge about mechanism of action

The recombinant protein-based subunit vaccine approach has an advantage of safety and multiple-booster dosing relative to inactivated or live-attenuated virus and nucleic acid vector-based vaccine formats, in addition to allowing for the selective use of the most dominant epitopes to generate potent neutralizing Ab titers [15, 17]. However, the relatively smaller size of the recombinant proteins may pose a problem of lower immunogenicity compared to a whole virus Ag, and therefore require additional features to enhance immunogenicity. The following is a brief discussion of the immunological mechanisms that form the basis of Akston's approach for developing its *immune-enhanced* recombinant subunit vaccine, AKS-452, and emphasizes its distinguishing factors from other closely related vaccine programs.

With respect to a basic immune response, injection of any protein Ag can, and most likely will, induce an immune response, the magnitude and type of which is highly dependent on the "status" of the respective immune system. For example, injection of a foreign Ag relative to a self Ag will induce a greater immune response in an immune system that maintains central and peripheral tolerance mechanisms, while self Ag can elicit significant immune responses in an immune system with broken tolerance mechanisms, such as an autoimmune condition. Moreover, foreign or self Ag administration to an immune system that has been primed to previous exposure to the respective Ag (e.g., a viral infection or an autoimmune disease) will lodge a more rapid and elevated immune response relative to that of an Ag-naïve system. The immunological basis of this priming is two-fold; 1) an Ag-naïve immune system has naïve B and T lymphocytes that have a much higher threshold of activation than do the Ag-primed "memory" cells of a Ag-primed immune system, such that the antigen-presenting cells (APCs) that present Ag require much less Ag to activate primed memory T cells, and 2) due to expansion of memory T cells during the Ag priming exposure, there are inherently greater numbers of such cells upon re-exposure to an injected Ag. Note that dominant APCs are dendritic cells (DCs) and macrophages that present Ag in complex with Major Histocompatibility Complex (MHC) molecules on their surface to T cell Ag receptors (**Figure 1**). It is these APCs that can influence both the "magnitude" and "type" of response to Ag; e.g., the Th1 cell response is required to clear most viral and bacterial infections, in which virus-like or bacterial-like substances (non-Ag in nature) condition APCs to express key cytokines and

surface co-stimulatory molecules that, during Ag presentation, drive T cells to become the Th1 type. In fact, this APC activation is the conceptual basis of many immune enhancing substances called *adjuvants*. Some adjuvants are designed to trick the immune system into reacting to the injected vaccine Ag as if it were part of an on-going infection (i.e., infectious agents provide such natural viral or bacterial adjuvant substances). Therefore, adjuvants activate APCs for greater Ag-presentation capabilities necessary to overcome the high activation threshold of naïve T cells, in addition to shaping their development into the Th1 response to effectively clear the respective infection. Note that such T cells provide critical help to B cells that specifically bind the respective Ag to produce Ag-specific antibody (Ab) titers (**Figure 2**).

Given the challenges of a recombinant SARS-Cov-2 SP subunit vaccine to induce a strong protective immune response in an immunologically naïve human population, the SP Ag must be modified and/or formulated with additional immune-enhancing features to overcome the activation thresholds of naïve T and B cells. Akston has implemented the following such features into its COVID-19 vaccine that are major advantages over most other such vaccines in development, in which the Therapeutic Product Profile (TPP) describes details of its clinical candidate, AKS-452 (Table 1):

- The use of the smaller focused antigenic portion of SP, the RBD
- Recombinant fusion of RBD with human IgG1 Fc (SP/RBD-Fc)

The following are explanations of the above features:

The focused immunogenicity of the RBD Ag leads to only those Abs that bind this region on SASRS-Cov-2 SP to prevent virus binding to the ACE2 target protein on host cells, thus inhibiting infection. This is in contrast to the use of a whole SP Ag vaccine that risks the generation of non-RBD-binding Abs that actually facilitate viral infection by tagging the virus for Fcg receptor (FcgR)-mediated uptake by macrophages that act as cellular factories for viral replication (i.e., ADE). Perhaps of even greater value is that the small size of RBD provides for at least a 10-fold greater production yield relative to SP (Akston's unpublished observation).

However, simply injecting such a small foreign protein fragment alone as a vaccine Ag would not be expected to induce a strong enough B cell (Ab) or Th1 cell response from a naïve immune status. Therefore, Akston created the subunit vaccine, AKS-452, comprised of a bivalent analog of RBD recombinantly fused to a human IgG1 Fc moiety (**Figure 3**) that (i) facilitates the focused delivery of the RBD Ag to local APCs that internalize SP/RBD-Fc via FcγRs, and then process and present RBD fragments (**Figure 4**) [34] to CD4+ Th cells that in turn promote ("help") B cell activation and anti-SARS-CoV-2 RBD IgG (i.e., Ab) production (**Figure 2**). In addition, a more direct and unique mechanism of AKS-452 is its direct binding to existing SARS-CoV-2-specific memory B cells through their Ag-specific B cell receptors (BCRs). Such binding triggers activation signals upon BCR cross-linking via the RBD bivalency feature of AKS-452 that leads to enhanced proliferation and anti-SARS-CoV-2 IgG production in the absence of CD4+ Th cells (**Figure 2**). Indeed, fusion of IgG Fc with a different RBD fragment derived from the SP of the SARS virus (i.e., SARS-CoV) has been demonstrated to impart significant adjuvant activity relative to the very low immunogenicity of the SARS-RBD fragment alone [15, 17]. In fact, this human IgG Fc-fusion enhancing approach has been

demonstrated with the development of a MERS vaccine containing recombinant protein of a truncated MERS SP/RBD fragment (residues 377-588) fused to human IgG Fc that increased immunogenicity via FcgR-binding on APCs, in addition to increasing the in vivo half-life and stability [18, 32]. That is, Fc enhances the systemic half-life and bioexposure of RBD to more APCs residing throughout the body due to binding the neonatal FcR (FcRn) expressed on endothelial cells that enables long serum half-lives of most monoclonal Ab (mAb) therapeutics. Another advantage of fusing RBD with Fc is the bivalency of the Ag per Fc molecule (i.e., two RBD fragments to one Fc fragment) that improves the stoichiometric quantity of Ag delivered to APCs.

However, the Fc feature of Akston's vaccine Ag may have limited use to only RBD-primed individuals who had a prior infection of SARS-Cov-2, both asymptomatic and symptomatic for COVID-19. That is, FcgR binding and activation signals in APCs, while known to provide significant signals for Ag presentation, are typically not strong enough to achieve the activation threshold of naïve lymphocytes, although these signals would be expected to re-activate the low-threshold of memory T and B cells of primed individuals (**Figure 1**). In addition, the weak signaling of FcgR in naïve lymphocytes does not ensure commitment to Th1 development. In summary, the Fc moiety on AKS-452 is designed to act as a mild adjuvant via inducing activation signalling to the APC via FcgRs and is designed to enhance the duration of Ag exposure to APCs and perhaps direct Ag entry into lymph nodes locally and systemically where additional APCs reside. As a consequence, the Fc moiety is expected to create a dramatic dose-sparing potential for both the Ag and adjuvant such that the risk of reactogenicity (a safety concern) is dramatically reduced; i.e., too much adjuvant that over-activates many APCs and other innate immune cells can lead a systemic inflammatory reaction termed reactogenicity. Such reactogenicity is induced acutely after injection and is not mediated by T and B cells.

<u>b. Previous exposure of human beings with the test product(s) and/or products with a similar biological mechanism</u>

The AKS-452 vaccine has been evaluated in a combined phase I/II clinical study (https://clinicaltrials.gov/show/NCT04681092). The initial data of the phase I study have been evaluated by the DSMB (see Appendix A). Phase II is currently ongoing and as part of an ongoing combined phase I/II clinical study, expected to be finalized February 2022, last follow-up.

<u>c. Can the primary or secondary mechanism be induced in animals and/or in *ex-vivo* human cell material?</u>

Yes, the vaccine is potent at inducing high titers of neutralizing Abs after single and double low-dose administrations via s.c. routes in mice, rabbits, non-human primates and humans (phase I/II).

<u>d. Selectivity of the mechanism to target tissue in animals and/or human beings</u>

The selectivity has been extensively tested and evaluated in human cells and animals (see IB / IMPD), and in human beings through a phase I/II study.

e. Analysis of potential effect

The vaccine is composed of one main component: the SP/RBD-Fc fusion protein antigen. The SP/RBD-Fc antigen is given at extremely low dose levels because of its design to function locally in the proximity of the injection site. Therefore, this component does have any associated serious practical risks.

f. Pharmacokinetic considerations

Because such low doses of the vaccine are administered, the vaccine does not achieve any detectable systemic levels.

g. Study population

Healthy volunteers

h. Interaction with other products

Not applicable

i. Predictability of effect

The levels of neutralizing Abs measured in the ACE2-SP/RBD binding ELISA that are as high as those in human convalescent serum are a good predictability marker of efficacy, which is the case with other vaccines already evaluated in Phase III COVID-19 efficacy trials. In addition, the induction of SP/RBD-specific Th1 T cells, measured via IFN- γ ELISAPOT assay, are confirmation that the vaccine induces the SARS-Cov-2 protective T cell response.

j. Can effects be managed?

For the following burden / side-effects the effects can be managed, although the effects are estimated to be low <1%.

- Pain / swelling / redness, at SC injection site: painkiller and cold packages
- Bleeding / inflammation / infection /granuloma formation, at SC injection site: bandage, observation / anti-inflammatory drug – antibiotics. Granuloma formation in general can be observed for regression – ultimately in case of discomfort after 3 –6 months a surgical removal of the granuloma
- Mild fever / chills / feeling tired / headache / muscle and joint aches during/after SC or IM injection: anti-inflammatory drugs (acetaminophen 500 or 1000 gram p.o., max 4000 gram per day)
- Syncope: observation / supine bed positioning
- Allergic reaction: In case of an allergic reaction, the Standard Operating Procedure 'Anaphylactic Reaction' is activated and in summary consists of the following escape medication: anti-histamine medication (e.g. cetirizine 10 mg p.o., dexamethasone (4 mg p.o.) and in case of a severe reaction epinephrine, oxygen and alarm UMCG Emergency Crash Team at the ward.

12.2 Synthesis

The vaccine AKS-452 has been extensively tested in a broad range of cells and animals (including non-human primates) without any significant toxicity. It can be concluded from the data presented in the IB / IMPD that the pharmacological profile of AKS-452 is fully within the range of clinical translation towards the phase I/II clinical study and the underlying phase II booster study.

The risks associated with exposure are pain/swelling/redness/bleeding/infection/granuloma formation at the SC injection site, mild fever, chills, feeling tired, headache, muscle and joint aches, syncope, and an allergic reaction which are all well within the tolerable range for a novel vaccine like AKS-452. The benefit, in case of a safe and sufficient immunogenicity provoking vaccine, is for protecting health care workers, future vulnerable and frail elderly, and patients undergoing large surgical procedures for instance oncology, transplantation etc. Moreover, providing protection in co-morbid citizens (i.e., diabetes, overweight, cardiovascular disease etc) and ultimately, creating another leverage to returning societies back to their previous health care system capacities and economic growth world-wide.

13. REFERENCES

1.Cascella, M., et al., *Features, Evaluation and Treatment Coronavirus (COVID-19)*, in *StatPearls*. 2020: Treasure Island (FL).

2.DailyMail, *A FIFTH New York City residents tested positive coronavirus antibodies.* https://www.dailymail.co.uk/news/article-8250385/A-FIFTH-New-York-City-residents-tested-positive-coronavirus-antibodies-Cuomos-study.html, 2020.

3.ABCNews, *When will we know if COVID-19 antibodies prevent reinfection?* https://abcnews.go.com/Health/covid-19-antibodies-prevent-reinfection/story?id=70288019, 2020.

4.Ward, H., et al., *Declining prevalence of antibody positivity to SARS-CoV-2: a community study of 365,000 adults.* medRxiv, 2020.

5.Reuters, *Explainer: Why are some South Koreans who recovered from the coronavirus testing positive again?* https://www.reuters.com/article/us-health-coronavirus-southkorea-explain-idUSKCN21Y0UE, 2020.

6.Bloomberg.com, *Coronavirus Survivors Hope for Immunity — The Reality Is More Complicated.* https://www.bloomberg.com/news/articles/2020-04-14/do-coronavirus-survivors-have-immunity-from-reinfection-maybe?utm_campaign=news&utm_medium=bd&utm_source=applenews, 2020.

7.Chen, Y., et al., *A comprehensive, longitudinal analysis of humoral responses specific to four recombinant antigens of SARS-CoV-2 in severe and non-severe COVID-19 patients.* PLoS Pathog, 2020. 16(9): p. e1008796.

8.Perreault, J., et al., *Waning of SARS-CoV-2 RBD antibodies in longitudinal convalescent plasma samples within four months after symptom onset.* Blood, 2020.

9.Wang, K., et al., *Longitudinal dynamics of the neutralizing antibody response to SARS-CoV-2 infection.* Clin Infect Dis, 2020.

10.Yin, S., et al., *Longitudinal anti-SARS-CoV-2 antibody profile and neutralization activity of a COVID-19 patient.* J Infect, 2020. 81(3): p. e31-e32.

11.Coutard, B., et al., *The spike glycoprotein of the new coronavirus 2019-nCoV contains a furin-like cleavage site absent in CoV of the same clade.* Antiviral Res, 2020. 176: p. 104742.

12.Zhou, P., et al., *A pneumonia outbreak associated with a new coronavirus of probable bat origin.* Nature, 2020. 579(7798): p. 270-273.

13.Lu, R., et al., *Genomic characterisation and epidemiology of 2019 novel coronavirus: implications for virus origins and receptor binding.* Lancet, 2020. 395(10224): p. 565-574.

14.Ahn, D.G., et al., *Current Status of Epidemiology, Diagnosis, Therapeutics, and Vaccines for Novel Coronavirus Disease 2019 (COVID-19).* J Microbiol Biotechnol, 2020. 30(3): p. 313-324.

15.Chen, W.H., et al., *The SARS-CoV-2 Vaccine Pipeline: an Overview.* Curr Trop Med Rep, 2020: p. 1-4.

16.Jiang, S., et al., *Roadmap to developing a recombinant coronavirus S protein receptor-binding domain vaccine for severe acute respiratory syndrome.* Expert Rev Vaccines, 2012. 11(12): p. 1405-13.

17.Lurie, N., et al., *Developing Covid-19 Vaccines at Pandemic Speed.* N Engl J Med, 2020.

18.Prompetchara, E., C. Ketloy, and T. Palaga, *Immune responses in COVID-19 and potential vaccines: Lessons learned from SARS and MERS epidemic.* Asian Pac J Allergy Immunol, 2020. 38(1): p. 1-9.

19.Bos, R., et al., *Ad26 vector-based COVID-19 vaccine encoding a prefusion-stabilized SARS-CoV-2 Spike immunogen induces potent humoral and cellular immune responses.* NPJ Vaccines, 2020. 5: p. 91.

20.Folegatti, P.M., et al., *Safety and immunogenicity of the ChAdOx1 nCoV-19 vaccine against SARS-CoV-2: a preliminary report of a phase 1/2, single-blind, randomised controlled trial.* Lancet, 2020. 396(10249): p. 467-478.

21.Mahase, E., *Covid-19: Johnson and Johnson vaccine trial is paused because of unexplained illness in participant.* BMJ, 2020. 371: p. m3967.

22.Ewer, K., et al., *Chimpanzee adenoviral vectors as vaccines for outbreak pathogens.* Hum Vaccin Immunother, 2017. 13(12): p. 3020-3032.

23.Mulligan, M.J., et al., *Phase I/II study of COVID-19 RNA vaccine BNT162b1 in adults.* Nature, 2020. 586(7830): p. 589-593.

24.Walsh, E.E., et al., *Safety and Immunogenicity of Two RNA-Based Covid-19 Vaccine Candidates.* N Engl J Med, 2020.

25.Anderson, E.J., et al., *Safety and Immunogenicity of SARS-CoV-2 mRNA-1273 Vaccine in Older Adults.* N Engl J Med, 2020.

26.Jackson, L.A., et al., *An mRNA Vaccine against SARS-CoV-2 - Preliminary Report.* N Engl J Med, 2020.

27.PFIZER AND BIONTECH CONCLUDE PHASE 3 STUDY OF COVID-19 VACCINE CANDIDATE, MEETING ALL PRIMARY EFFICACY ENDPOINTS. https://www.pfizer.com/news/press-release/press-release-detail/pfizer-and-biontech-conclude-phase-3-study-covid-19-vaccine, 2020.

28.European Medicines Agency Begins Rolling Review of Moderna's mRNA Vaccine Candidate Against COVID-19 (mRNA-1273). https://investors.modernatx.com/news-releases/news-release-details/european-medicines-agency-begins-rolling-review-modernas-mrna, 2020.

29.Du, L., et al., *Antigenicity and immunogenicity of SARS-CoV S protein receptor-binding domain stably expressed in CHO cells.* Biochem Biophys Res Commun, 2009. 384(4): p. 486-90.

30.Wong, S.K., et al., *A 193-amino acid fragment of the SARS coronavirus S protein efficiently binds angiotensin-converting enzyme 2.* J Biol Chem, 2004. 279(5): p. 3197-201.

31.Keech, C., et al., *Phase 1-2 Trial of a SARS-CoV-2 Recombinant Spike Protein Nanoparticle Vaccine.* N Engl J Med, 2020.

32.Zhang, N., et al., *Identification of an ideal adjuvant for receptor-binding domain-based subunit vaccines against Middle East respiratory syndrome coronavirus.* Cell Mol Immunol, 2016. 13(2): p. 180-90.

33.Du, L., et al., *The spike protein of SARS-CoV--a target for vaccine and therapeutic development.* Nat Rev Microbiol, 2009. 7(3): p. 226-36.

34.Loureiro, S., et al., *Virus Glycoproteins Tagged with the Human Fc Domain as Second Generation Vaccine Candidates.* Innovation in Vaccinology: From Design, Through to Delivery and Testing, 2012. Chapter 3.

35.Ko, E.J. and S.M. Kang, *Immunology and efficacy of MF59-adjuvanted vaccines.* Hum Vaccin Immunother, 2018. 14(12): p. 3041-3045.

36.Arevalo-Herrera, M., et al., *Preclinical vaccine study of Plasmodium vivax circumsporozoite protein derived-synthetic polypeptides formulated in montanide ISA 720 and montanide ISA 51 adjuvants.* Am J Trop Med Hyg, 2011. 84(2 Suppl): p. 21-7.

37.Aucouturier, J., et al., *Montanide ISA 720 and 51: a new generation of water in oil emulsions as adjuvants for human vaccines.* Expert Rev Vaccines, 2002. 1(1): p. 111-8.

38.Herrera, S., et al., *Phase I safety and immunogenicity trial of Plasmodium vivax CS derived long synthetic peptides adjuvanted with montanide ISA 720 or montanide ISA 51.* Am J Trop Med Hyg, 2011. 84(2 Suppl): p. 12-20.
39.Tifrea, D.F., et al., *Improved protection against Chlamydia muridarum using the native major outer membrane protein trapped in Resiquimod-carrying amphipols and effects in protection with addition of a Th1 (CpG-1826) and a Th2 (Montanide ISA 720) adjuvant.* Vaccine, 2020. 38(28): p. 4412-4422.
40.Motavalli Khiavi, F., et al., *A Dual-Type L2 11-88 Peptide from HPV Types 16/18 Formulated in Montanide ISA 720 Induced Strong and Balanced Th1/Th2 Immune Responses, Associated with High Titers of Broad Spectrum Cross-Reactive Antibodies in Vaccinated Mice.* J Immunol Res, 2018. 2018: p. 9464186.
41.Shokri, M., et al., *Comparing Montanide ISA 720 and 50-V2 adjuvants formulated with LmSTI1 protein of Leishmania major indicated the potential cytokine patterns for induction of protective immune responses in BALB/c mice.* Mol Immunol, 2016. 76: p. 108-15.
42.Qiu, Q., et al., *Induction of multispecific Th-1 type immune response against HCV in mice by protein immunization using CpG and Montanide ISA 720 as adjuvants.* Vaccine, 2008. 26(43): p. 5527-5534.
43.Langermans, J.A., et al., *Preclinical evaluation of a chimeric malaria vaccine candidate in Montanide ISA 720: immunogenicity and safety in rhesus macaques.* Hum Vaccin, 2006. 2(5): p. 222-6.
44.Oliveira, G.A., et al., *Safety and enhanced immunogenicity of a hepatitis B core particle Plasmodium falciparum malaria vaccine formulated in adjuvant Montanide ISA 720 in a phase I trial.* Infect Immun, 2005. 73(6): p. 3587-97.
45.Miles, A.P., et al., *Montanide ISA 720 vaccines: quality control of emulsions, stability of formulated antigens, and comparative immunogenicity of vaccine formulations.* Vaccine, 2005. 23(19): p. 2530-9.
46.Toledo, H., et al., *A phase I clinical trial of a multi-epitope polypeptide TAB9 combined with Montanide ISA 720 adjuvant in non-HIV-1 infected human volunteers.* Vaccine, 2001. 19(30): p. 4328-36.
47.Lawrence, G.W., et al., *Phase I trial in humans of an oil-based adjuvant SEPPIC MONTANIDE ISA 720.* Vaccine, 1997. 15(2): p. 176-8.
48.Ascarateil, S., A. Puget, and M.-E. Koziol, *Safety data of Montanide ISA 51 VG and Montade ISA 720 VG, two adjuvants dedicated to human therapeutic vaccines*
49. Krause, P., Fleming, T.R., Longini, I. et al., *World Health Organization Solidarity Vaccines Trial Expert Group. COVID-19 vaccine trials should seek worthwhile efficacy.* Lancet, 2020. 396: p741-743.
50. Ascarateil, S., A. Puget, and M.-E. Koziol, Safety data of Montanide ISA 51 VG and Montade ISA 720 VG, two adjuvants dedicated to human therapeutic vaccines. J Immunotherapy Cancer, 2015. 3(Suppl 2): p. 428.
51. Aucouturier, J., et al., Montanide ISA 720 and 51: a new generation of water in oil emulsions as adjuvants for human vaccines. Expert Rev Vaccines, 2002. 1(1): p. 111-8.
52. Miles, A.P., et al., Montanide ISA 720 vaccines: quality control of emulsions, stability of formulated antigens, and comparative immunogenicity of vaccine formulations. Vaccine, 2005. 23(19): p. 2530-9.
53. Mauri, L., D'agostino, R.B., Challenges in the design and interpretation of non-inferiority studies. N Engl J Med, 2017. 377: p.1357-1367.
54. Dal-Ré, R., et al., Ethical and scientific considerations regarding the early approval and deployment of a COVID-19 vaccine. Ann Intern Med, 2020. M20-7357.
